# Anther dehiscence is regulated by gibberellic acid in yellow lupine (*Lupinus luteus* L.)

**DOI:** 10.1186/s12870-021-03085-4

**Published:** 2021-07-02

**Authors:** Katarzyna Marciniak, Krzysztof Przedniczek

**Affiliations:** Faculty of Biological and Veterinary Sciences, Department of Plant Physiology and Biotechnology, Nicolaus Copernicus University, Lwowska 1 St, 87-100 Toruń, Poland

**Keywords:** Anther dehiscence, Gibberellins, Endothecium, Secondary thickening, Septum/stomium rupture, Degeneration via PCD, Yellow lupine, Legumes

## Abstract

**Background:**

Anther dehiscence resulting in the release of pollen grains is tightly regulated in a spatiotemporal manner by various factors. In yellow lupine (*Lupinus luteus* L.), a species that shows cleistogamy, the anthers split before the flowers open, but the course and regulation of this process are unknown. The specific control of anther development takes place via hormonal pathways, the wide action of which ensures reproductive success. In our previous research concerning flower and early pod development in yellow lupine, we showed that the lowest transcript level of *LlDELLA1*, a main repressor of gibberellin (GA) signalling, occurs approximately at the time of anther opening; therefore, the main purpose of this study was to precisely investigate the gibberellic acid (GA_3_)-dependent regulation of the anther dehiscence in this species.

**Results:**

In this paper, we showed the specific changes in the yellow lupine anther structure during dehiscence, including secondary thickening in the endothecium by lignocellulosic deposition, enzymatic cell wall breakdown at the septum/stomium and cell degeneration via programmed cell death (PCD), and identified several genes widely associated with this process. The expression profile of genes varied over time, with the most intense mRNA accumulation in the phases prior to or at the time of anther opening. The transcriptional activity also revealed that these genes are highly coexpressed and regulated in a GA-dependent manner. The cellular and tissue localization of GA_3_ showed that these molecules are present before anther opening, mainly in septum cells, near the vascular bundle and in the endothecium, and that they are subsequently undetectable. GA_3_ localization strongly correlates with the transcriptional activity of genes related to GA biosynthesis and deactivation. The results also suggest that GA_3_ controls *LlGAMYB* expression via an *LlMIR159*-dependent pathway.

**Conclusions:**

The presented results show a clear contribution of GA_3_ in the control of the extensive anther dehiscence process in yellow lupine. Understanding the processes underlying pollen release at the hormonal and molecular levels is a significant aspect of controlling fertility in this economically important legume crop species and is of increasing interest to breeders.

**Supplementary Information:**

The online version contains supplementary material available at 10.1186/s12870-021-03085-4.

## Background

The critical factors in plant reproduction are the proper development of stamens and the release of viable pollen grains, followed by pollination, fertilization and fruit/pod setting and development. This is particularly important in crop plants, including legumes, due to the impact on yield. Knowledge of stamen development is increasing, but most of the information comes from analyses of male-sterile mutants in *Arabidopsis thaliana* and rice (*Oryza sativa*) [1–5]. Fourteen early and late phases of stamen development have been identified in the model plant *A. thaliana* [1, 6, 7]. The fundamental and extensive processes that occur in maturing stamens is anther dehiscence, which generally consists of the following stages: (I) expansion of the endothecium and deposition of materials responsible for wall thickening in endothecial and connective cells; (II) degeneration of the tapetum and middle layer (stages 7–11); (III) enzymatic opening of the septum between two locules and its progressive degeneration (stages 11–12); (IV) breakage of the stomium formed from modified epidermal cells (stage 12); and (V) the release of pollen grains from the locules during stage 13. Finally, the anthers senesce and are separated from flowers (stages 14a-14c) [1, 2]. Many species, such as *A. thaliana*, tobacco (*Nicotiana tabacum*) and rice, exhibit comparable courses of anther dehiscence [1, 8–10]. The differentiation and degeneration of the septum (circular cell cluster in *N. tabacum*) and stomium are conserved [8]; however, developmental differences exist, e.g., in the anther structure or type of endothecial thickening [11, 12]. Much evidence has demonstrated the crucial role of secondary cell wall thickening in pollen release. It has been suggested that this is closely related to the delivery of force to disrupt stomium cells [2, 4, 11]. The key components of secondary cell walls are lignin, cellulose and hemicelluloses (xylan and glucomannan). Lignin consists of three subunits, namely, p-hydroxyphenyl (H), guaiacyl (G) and syringyl (S), and the most important enzyme catalysing the last step of lignin biosynthesis is cinnamyl alcohol dehydrogenase (CAD) [13]. In *A. thaliana*, two *CAD* genes (*CAD C*, *CAD D*) have been identified, and mutants of these genes show the absence of lignin in the endothecium, leading to anther indehiscence [14]. Among the genes involved in secondary cell wall formation, *IRREGULAR XYLEM1/6/8* (*IRX1/6/8*) can also be distinguished [15–17]. *IRX1*, with the alternative name *cellulose synthase A catalytic subunit 8* (*CesA8*), is required for the synthesis of cellulose [16]; *IRX6* (synonym *COBRA-like4*, *COBL4*) encodes a member of the COBRA family that is needed for the deposition of cellulose whereas *IRX8* (synonym *galacturonosyltransferase 12*, *GAUT12*) belongs to the CAZy family of glycosyl transferase (GT8), which is involved in the deposition of xylan and lignin in secondary cell walls [15, 18]. One of the major events in anther dehiscence is pectin degradation leading to cell separation, which involves enzymes such as polygalacturonases (PGs). QUARTET2 (QRT2), part of a small family of endo-PGs, is involved in the anther dehiscence process [4, 19]. Many papers have shown that the septum and stomium undergo a process of degeneration and cell death via a developmental programmed cell death (PCD)-related process, as part of dehiscence. Furthermore, plant cells usually undergo PCD in response to hormone-mediated signalling pathways [8, 20]. Among the genes associated with PCD, in *A. thaliana*, *PROMOTION OF CELL SURVIVAL1* (*PCS1*) has been recognized as an important factor encoding an aspartic protease that acts during reproduction and embryogenesis. Using transgenic plants that overexpressed *PCS1*, Ge et al. showed that this gene inhibits the individual elements of the PCD pathway in anthers [21].

Examination of late-dehiscence mutants revealed that the time of anther opening is strictly controlled by various factors, including phytohormones. Usually, *A. thaliana* mutants that are defective in phytohormone biosynthesis or perception are male sterile and have anthers that split too late for effective pollination and fertilization [8, 9, 22, 23]. Gibberellins (GAs) play an important role in the control of pollen formation and viability, filament elongation and anther dehiscence [5, 24–28]. Late GA biosynthesis stages are catalysed by gibberellin 3-oxidases (GA3oxs) responsible for the production of active phytohormone molecules and gibberellin 2-oxidases (GA2oxs) that deactivate GAs. The central factor in the GA signalling pathway is *GAMYB*, which belongs to the R2R3-MYB subfamily. The results of research on barley (*Hordeum vulgare*) show that overexpressing *HvGAMYB* results in a phenotype with indehiscent anthers [29]. Another study revealed that *GAMYB* acts in cooperation with microRNAs, especially miR159 [30]. These facts indicate that a complex set of interactions is required to coordinate events within the flower that lead to anther dehiscence.

The developmental timing of anther opening influences the type of pollination and degree of self-pollination in comparison with cross-pollination. This is important for seed production in breeding programs and agriculture. In general, pollen release occurs when flowers are fully open, in *A. thaliana* at the flower stage 13 [31]; however, in some plants, including yellow lupine (*Lupinus luteus* L.), a special type of self-pollination in a closed flower (cleistogamy) was found. Yellow lupine is also characterized by the ability to fix atmospheric nitrogen, and high protein content in seeds; therefore, it is used worldwid as a food and feed source. Nevertheless, this legume plant has the problem of premature flower abscission [32–35]. For years, there has been a hypothesis that the reason for excessive flower abscission may be incorrect stamen development and, as a consequence, insufficient pollination, fertilization, and pod setting, which reduce yield. Our earlier study showed that changing the level of *LlDELLA1* mRNA, encoding major repressors of GA signalling, supports correct flower and pod development [34]. Interestingly, the expression of *LlDELLA1* in the flower bud phase was relatively high and then decreased slightly until anther opening; during pollination, fertilization and early pod development, a gradual increase in transcript levels was observed. This indirectly indicates the involvement of GAs in flower development. In different plant species, research on the role of GAs in stamen development has focused on the early stages, and there are few or no available data on their effects in late stages, including anther dehiscence. Therefore, the main aim of this study was to understand the unexplored process of GA-modulated late anther development in yellow lupine, which may lead to the control of male fertility in the future and aid in eliminating the problem of low yield. We focused on genes and proteins that could be potential markers of developmental changes taking place in yellow lupine anthers. Examination of the expression profile of genes widely associated with dehiscence after the application of various compounds (gibberellic acid, GA_3_ and paclobutrazol, PAC) in conjunction with GA_3_ immunolocalization provides broad insight into the regulation of the anther development process in yellow lupine. The function of predicted proteins was defined based on the presence of conserved domains, motifs and specific amino acids. The research is complemented by histological analyses to determine the changes in the structure of the yellow lupine anther during the process of extensive dehiscence.

## Results

### Specific changes in structure of yellow lupine anthers in different stages of late development

**Fig. 1:**
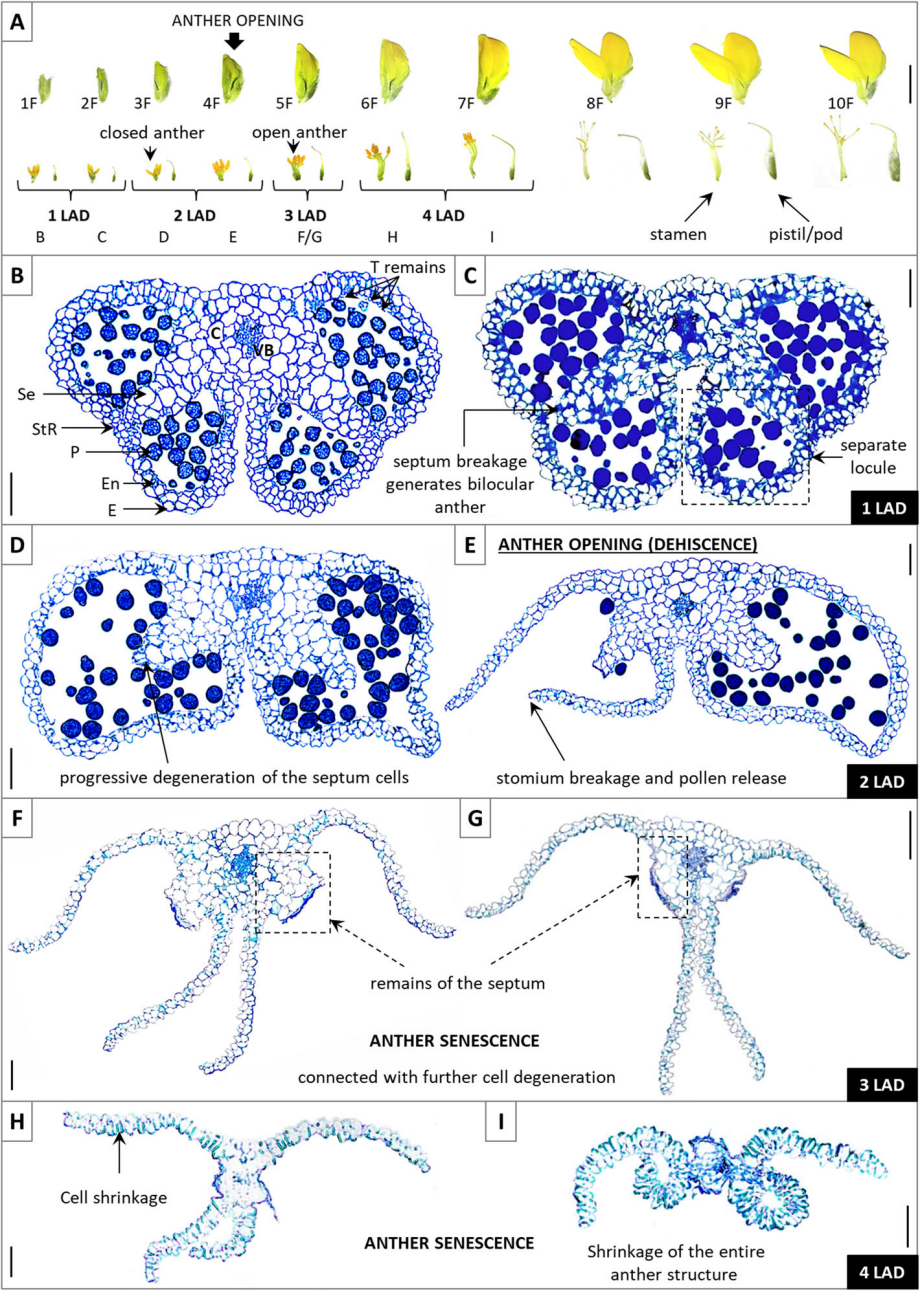
**A** Individual stages of flower (1F–10F) development in yellow lupine. After completing the dehiscence (1F–3F), anther opening occurred at approximately the fourth phase of flower development (4F) when the flower was completely closed. Then, pollination, fertilization, and pod setting and development occurred (5F–10F). The first and second stages of flower development correspond to the first stage of late anther development (LAD); the third and fourth stages of flower development correspond to the second stage of LAD; the fifth stage of flower development is parallel to the third stage of LAD; and the sixth and seventh stages of flower development correspond to the fourth stage of LAD. **B-I** The anatomical structure of yellow lupine anthers in different stages of late development. Cross-sections were stained with toluidine blue, and anthers were photographed by light microscopy. C – connective, VB – vascular bundle, Se – septum, StR – stomium region, P – pollen grain, En – endothecium, E – epidermis, T – tapetum. Scale bars = 1 cm (A); 50 μm (B-I)

The individual stages of yellow lupine late anther development (LAD), when dehiscence takes places, were selected (Fig. 1A). In the first phase of LAD (1 LAD), which corresponds to *A. thaliana* phase 11, four separated locules with pollen were distinguished (Fig. 1B). Therefore, the anther is bithecal and tetrasporangiate. In the centre of the yellow lupine anther, connective tissue containing a vascular bundle with a septum on both sides (which separate the locules) was found. Moreover, septum cells are characterized by different sizes depending on their location, with a tendency that cells closer to the anther centre are much larger. The wall surrounding the anther locules consists of the following cell layers: epidermis, endothecium, middle layer, and tapetum. The externally located epidermis is composed of one layer of longitudinally elongated cells. The differentiated epidermal cells form the stomium region adjacent to septum cells. The next layer consists of transversely elongated endothecial cells with visible secondary cell wall thickening. Additionally, the remains of the tapetum were visible in locules (Fig. 1B). In the 1 LAD phase, breakage of the septum from the stomium was also noticed (Fig. 1C). This generates a bilocular anther, and further degeneration of the septum cells was evident in the second LAD phase (2 LAD) (corresponding to *A. thaliana* phase 12) (Fig. 1D). The stomium forms a single-cell region, the location of which determines the position of anther opening. Late septum shrinkage and disruption seem to contribute to breaking the stomium, which leads to pollen release (these processes occur simultaneously). The dehiscence process does not affect the central connective cells, which remain joined. Yellow lupine anther opening occurs in 2 LAD, which corresponds to phase 13 in *A. thaliana* (Fig. 1E). Following dehiscence, anther senescence occurs (3 LAD, Fig. 1F/G), characterized by further degeneration and shrinkage of cells, and the entire anther structure (4 LAD, Fig. 1H/I).

### Secondary thickening of endothecial cell walls

**Fig. 2:**
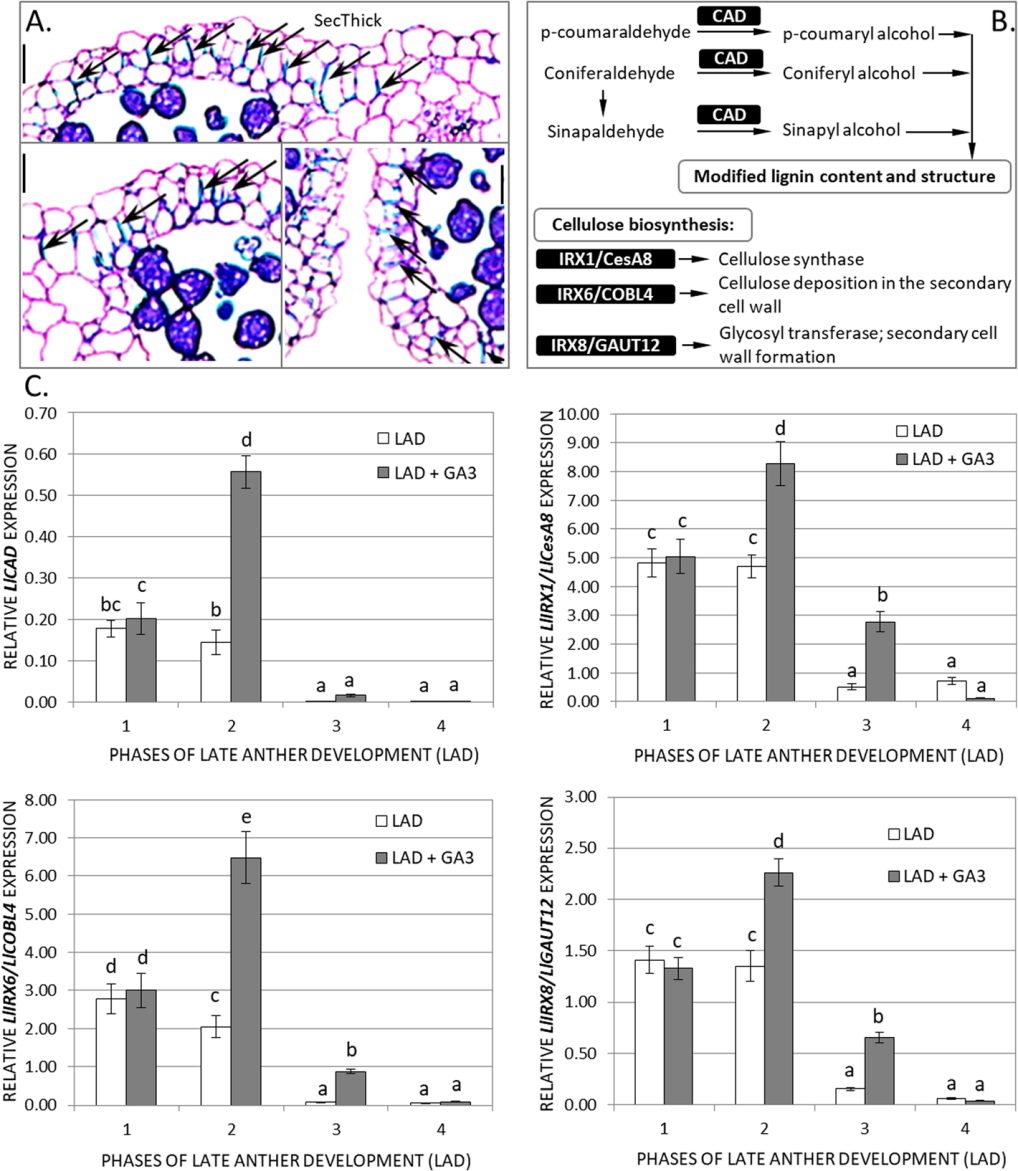
**A** Localization of secondary thickening (SecThick) within endothecial cell walls of yellow lupine anthers. Transverse sections were stained with toluidine blue. Anthers were photographed by light microscopy. **B** The function of typical lignin (*CAD*, *CINNAMYL ALCOHOL DEHYDROGENASE*) and cellulose (*IRX1/CesA8, IRREGULAR XYLEM1*/*Cellulose synthase A catalytic subunit 8*; *IRX6*/*COBL4*, *COBRA-like4*; *IRX8*/*GAUT12*, *GAlactUronosylTransferase12*) biosynthesis-related genes [15, 18, 36, 37]. **C** Transcriptional activity of investigated genes (related to *LlACT*) during late anther development (LAD) and after gibberellic acid treatment (LAD + GA_3_, 100 μM). Data are the mean ± SE of three biological replicates, each with two technical replicates. Letters represent statistically significant differences at *p* < 0.05 (one-way ANOVA followed by Tukey’s honest significant difference test). Scale bars = 25 μm

In the first stage of yellow lupine anther dehiscence, the endothecium, which partially surrounds the locule, undergoes the formation and deposition of secondary thickening (Fig. 2A). The endothecial cell walls show U-shaped thickening. The same occurs in the connective cell walls but to a much lesser extent. Other anther cells, which create, for example, epidermis, septum or stomium region, do not undergo thickening, which suggests that areas of secondary thickening are strictly localized in the endothecium.

The expression profiles of secondary wall-associated genes (Fig. 2B) with and without GA_3_ application in all LAD phases in yellow lupine were established (Fig. 2C). In the first and second LAD phases, significantly higher expression levels of *LlCAD* and all *LlIRX* genes were observed compared to those in the third and fourth LAD phases. In addition, GA_3_ treatment increased the number of lignin/cellulose/xylan biosynthesis gene transcripts, especially in the second LAD phase. It can be concluded that the genes related to secondary thickening of the endothecial cell walls show co-upregulated expression, and their transcriptional activity is mainly associated with changes in the first and second LAD stages of yellow lupine. Additionally, it appears that the levels of transcripts of all identified genes are GA dependent.

### Breakdown of the septum/stomium cells

**Fig. 3:**
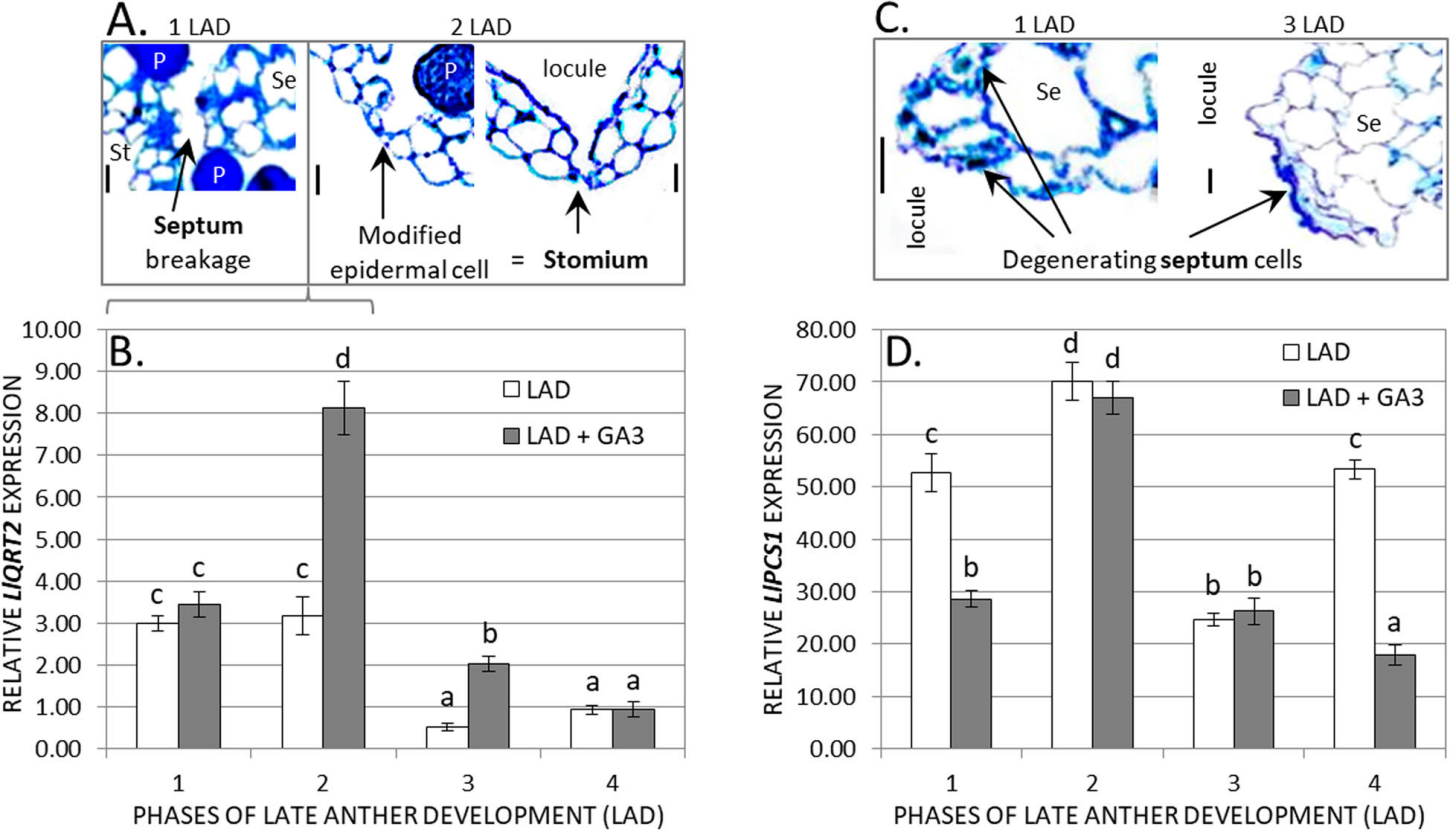
**A** Rupture of the septum (Se) and stomium (St) cells in the first and second late anther developmental (LAD) stages of yellow lupine; **C** Progressive degeneration of septum cells in the first and third LAD stages. All transverse sections were stained with toluidine blue. Anthers were photographed by light microscopy. **B**, **D** Expression profile of *LlQRT2* (*QUARTET2*) and *LlPCS1* (*PROMOTION OF CELL SURVIVAL1*) in relation to *LlACT* (*ACTIN*) during different stages of LAD, as well as after GA_3_ application (LAD + GA_3_, 100 μM). P – pollen grain. Data are the mean ± SE of three biological replicates, each with two technical replicates. Letters represent statistically significant differences at *p* < 0.05 (one-way ANOVA followed by Tukey’s HSD test). Scale bars = 10 μm

Cell separation is an important event that takes place in yellow lupine anthers. Dehiscence involves interruption of the cell wall material between adjacent cells (Fig. 3A). It is likely that endo-PGs and phytohormones are involved in this process. In selected stages of LAD, we examined the transcriptional activity of *LlQRT2*, encoding the marker enzyme involved in breakdown of pectin between cells (Fig. 3B). The results indicate that the amount of *LlQRT2* mRNA is high in the first and second LAD phases, when the breakdown of the septum/stomium takes place. Lower levels of transcripts were detected in the third and fourth LAD phases. Interestingly, *LlQRT2* expression was stimulated by GA_3_, especially in the second LAD stage.

### Degeneration of septum/stomium cells via PCD-related processes

The yellow lupine anther septum and stomium undergo degeneration and cell death to facilitate pollen release via GA-dependent PCD-related processes (Fig. 3C). To prove this, we determined the expression profile of *LlPCS1*, which encodes aspartic protease and is an anti-cell death component. As expected, GA_3_ application decreased the transcriptional activity of *LlPCS1*, especially in the first and fourth stages of LAD (Fig. 3D). This indirectly shows that GA_3_ favours this process in yellow lupine anthers by inhibiting the anti-PCD factor.

### Cellular and tissue localization of gibberellic acid during yellow lupine anther development

**Fig. 4:**
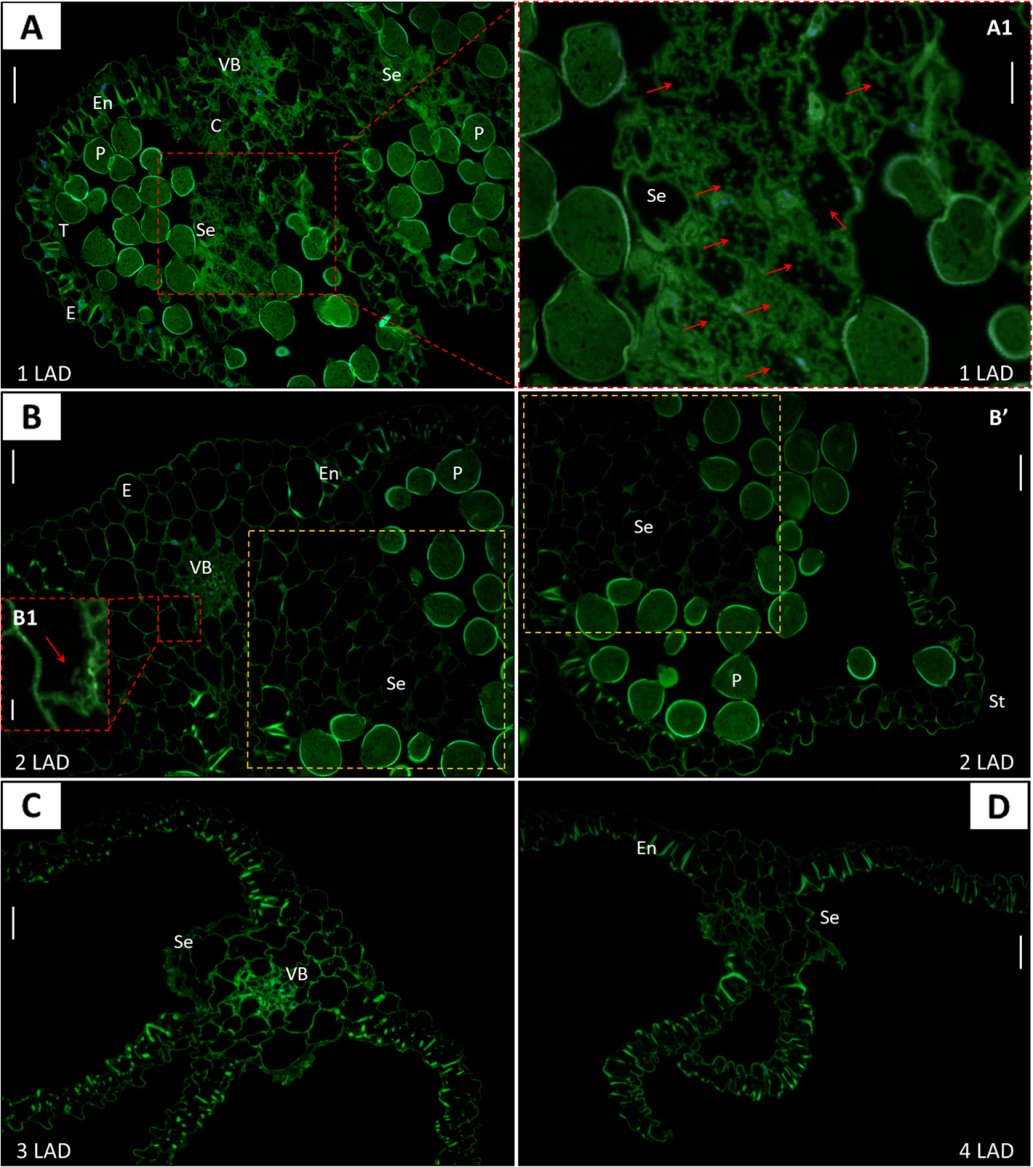
Gibberellic acid (GA_3_) immunolocalization in selected stages (1–4) of late anther development (LAD) in yellow lupine. Green fluorescence corresponds to GA_3_ accumulation, and blue fluorescence indicates cell nuclei stained with DAPI. Subfigures A1/B1 are an enlargement of subfigures A/B, respectively, in the places marked with the red squares. The red arrows indicate GA_3_ signal in the selected magnified cells. The yellow squares marked with a dashed line in subfigures B and B′ indicate the same area of cells. Autofluorescence of the cell walls and pollen grains was visible. VB – vascular bundle, P – pollen grain, E – epidermis, En – endothecium, T – tapetum, C – connective, Se – septum, St – stomium. Scale bars: 25 μm (A, B, B′, C, D), 10 μm (A1) and 5 μm (B1)

We performed cellular and tissue immunolocalization of GA_3_ in all selected stages of anther maturation in yellow lupine (Fig. 4). The results show that the highest accumulation of GA_3_ occurred in the first LAD phase before anther opening (Fig. 4A). At the cellular level, the strongest fluorescence signal was noted in the entire cytoplasm of degenerating septum cells at the time of bilocular anther formation (the cells were filled with GA_3_; Fig. 4A1). A high level of phytohormone molecules was also observed near the vascular bundle, in the cells of the middle layer and endothecium, and in the epidermis to a lesser extent. In the second LAD phase, when the stomium cells were disrupted and the pollen chambers opened, the fluorescence signal indicating GA_3_ content was almost imperceptible (Fig. 4B). GA_3_ was found in the cells of the progressively degenerating septum, middle layer, and near the vascular bundle (Fig. 4B1). After the anther was opened and mature pollen grains were released (3 LAD stage), no fluorescence signal indicating the presence of GA_3_ was observed (Fig. 4C). GA_3_ signal was also not detected when complete cell degeneration and ageing of the anther occurred (4 LAD stage). The lack of cell nuclei was also noticeable, which indicates the rapidly progressing processes of degeneration and death of entire cells of the anther (Fig. 4D).

### GA_3_ localization correlates with GA metabolism

**Fig. 5:**
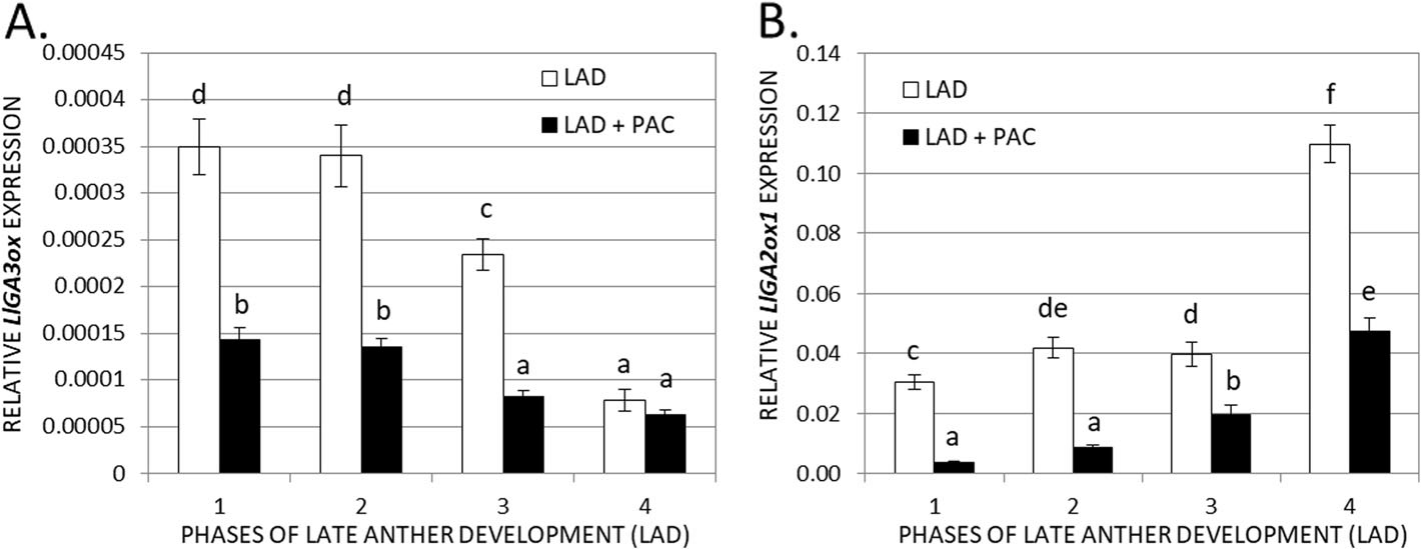
The relative transcript levels of *LlGA3ox* (*gibberellin 3-oxidase*) connected with GA biosynthesis **(A)** and *LlGA2ox1* (*gibberellin 2-oxidase 1*) involved in GA deactivation (**B**) were investigated during late anther development (LAD) of yellow lupine. Some anthers were treated with a solution of paclobutrazol (LAD + PAC, 100 μM) in 0.05% Tween 20, and other anthers were treated with only 0.05% Tween 20 (LAD). *LlACT* (*ACTIN*) was used as an internal control. Data are the mean ± SE of three biological replicates, each with two technical replicates. Letters represent significant differences at *p* < 0.05 (one-way ANOVA followed by Tukey’s HSD test)

The expression profiles of *LlGA3ox* involved in the formation of active phytohormone molecules and *LlGA2ox1* responsible for GA inactivation were determined (Fig. 5) to verify whether they are correlated with the endogenous GA_3_ level. In subsequent stages of LAD, a decrease in *LlGA3ox* mRNA levels was observed (Fig. 5A). In the case of *LlGA2ox1*, the opposite situation was found (Fig. 5B). Furthermore, the use of PAC, which inhibits the early stage of GA biosynthesis, significantly reduced the expression of both studied genes.

### GA_3_ controls LlGAMYB expression via a miR159-dependent pathway during late anther development

**Fig. 6:**
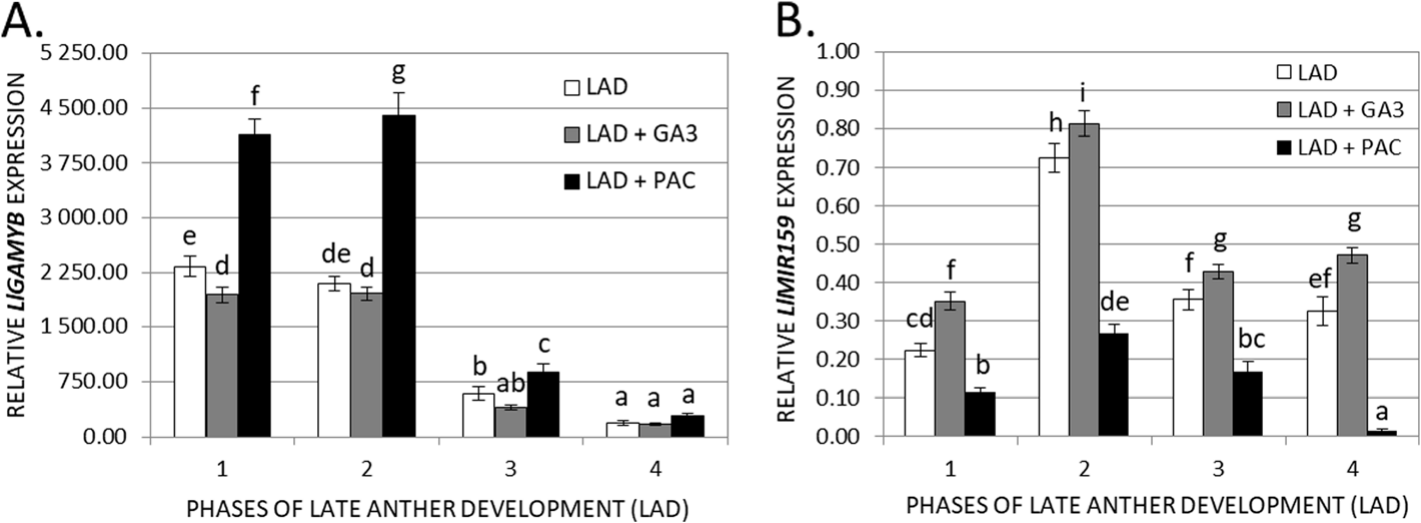
Relative transcriptional activity of *LlGAMYB* involved in GA signalling **(A)** and *LlMIR159* associated with cutting *GAMYB* transcripts (**B**) during late anther development (LAD) of yellow lupine. Some anthers were treated with a solution of GA_3_ (LAD + GA_3_, 100 μM) in 0.05% Tween 20, others were treated with a solution of paclobutrazol (LAD + PAC, 100 μM) in 0.05% Tween 20, and some were treated with only 0.05% Tween 20 (LAD). *LlACT* (*ACTIN*) was used as an internal control. Data are the mean ± SE of three biological replicates, each with two technical replicates. Letters represent statistically significant differences at *p* < 0.05 (one-way ANOVA followed by Tukey’s HSD test)

In yellow lupine, GA_3_ likely participates in the regulation of *LlGAMYB* by controlling the *LlMIR159* expression level. The *LlGAMYB* and *LlMIR159* transcriptional activity in four selected phases of LAD, as well as after GA_3_ and PAC application, were examined (Fig. 6). The *LlGAMYB* expression was almost identical with or without GA_3_ treatment, but the lack of GA_3_ due to PAC application resulted in increased *LlGAMYB* transcripts, especially in the first and second LAD stages (Fig. 6A). These results suggest that GA_3_ possibly indirectly regulates *LlGAMYB* expression. Therefore, we examined the *LlMIR159* expression profile (Fig. 6B). GA_3_ treatment increased *LlMIR159* mRNA levels, and importantly, PAC application significantly decreased the expression of *LlMIR159*. By analysing the natural conditions without the application of any compounds, it can be concluded that *LlGAMYB* expression is high in the first two stages of LAD and then decreases. This negatively correlates with the transcriptional activity of *LlMIR159*, which was significantly increased in the second phase of LAD. This is probably the cause of the reduced mRNA content of *LlGAMYB* in the third and fourth LAD stages. The results suggest that *LlGAMYB* is coexpressed with *LlMIR159* in yellow lupine anthers. It also follows that the *LlGAMYB* transcript level is potentially regulated by miR159 in the anthers of yellow lupine.

### In silico analyses of studied genes

**Fig. 7:**
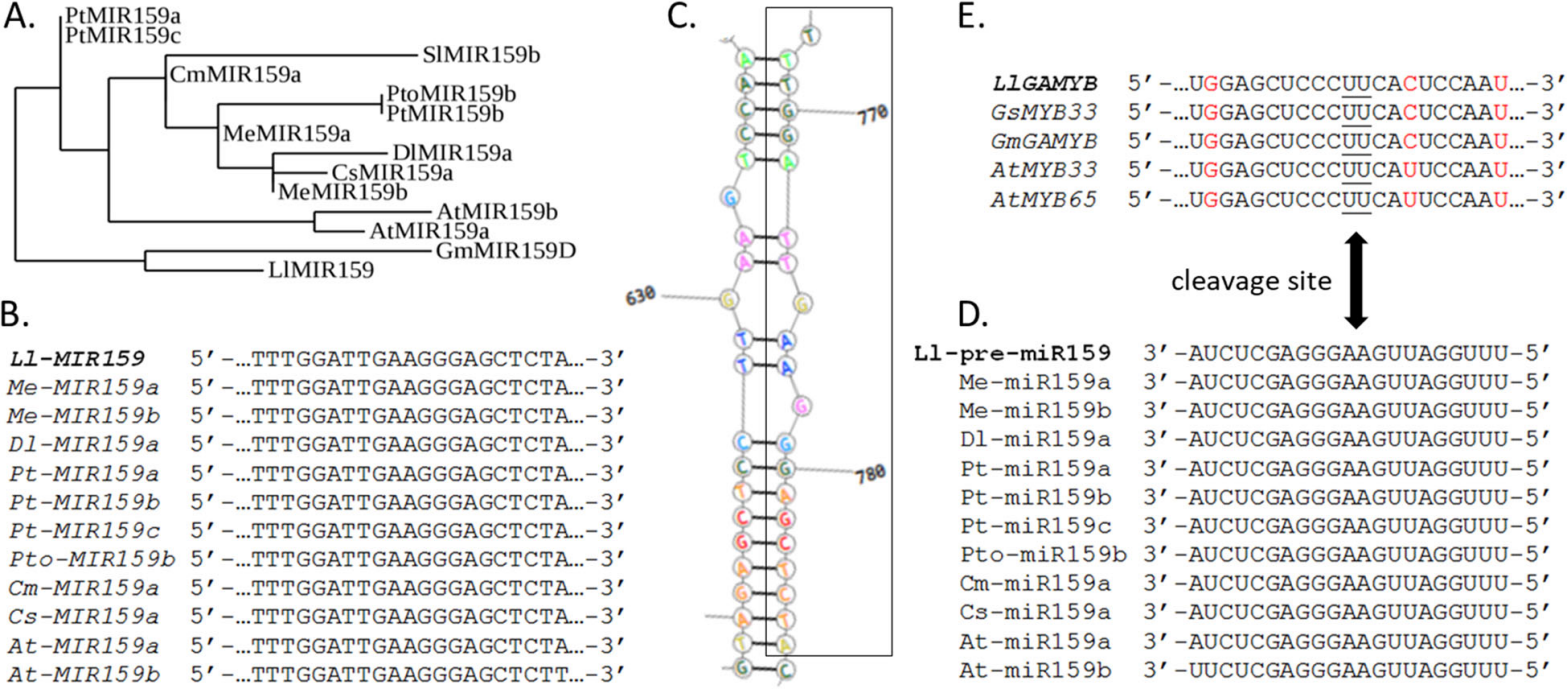
Analysis of the Ll-pre-miR159 sequence identified in yellow lupine; **(A)** The phylogenetic relationship of *Ll-MIR159* compared with *MIR159* in various plant species; **(B)** Alignment of part of the nucleotide sequence (21 bp, which forms a mature miRNA) of *Ll-MIR159* with closely related fragments of *MIR159* in other plant species; **(C)** The fragment of secondary stem-loop structure of Ll-pre-miR159 with localization of mature miR159 marked with a rectangle; **(D)** Alignment of the nucleotide sequences of Ll-pre-miR159 with a mature miR159 in different plant species; **(E)** *LlGAMYB* mRNA cleavage site designated between the 11th and 12th bases from the 5′ end (underlined) and comparison to other plant species. Red letters represent mismatches between *GAMYB* sequences and miR159s; *Ll* – *Lupinus luteus* (MW240683, *Ll-MIR159*; MW240675, *LlGAMYB*), *Me* – *Manihot esculenta* (JX013999, JX014000), *Dl* – *Dimocarpus longan* (MT920321), *Pt* – *Populus trichocarpa* (AY728394, AY728395, AY728401), *Pto* – *Populus tomentosa* (MF463031), *Cm* – *Cucumis melo* (NR_120776), *Cs* – *Citrus sinensis* (NR_129302), *At* – *Arabidopsis thaliana* (NR_139941/At-miR159a, NR_139756/At-miR159b, AAS10086/*AtMYB33*, AAS10055/*AtMYB65*), *Gs - Glycine soja* (XP_028187659), *Gm* – *Glycine max* (AHB19229)

The identification of many genes encoding enzymes connected with anther dehiscence prompted us to perform bioinformatics analyses. The full-length cDNA sequences identified in yellow lupine, their deduced amino acid sequences, molecular weights, predicted isoelectric points and NCBI accession numbers are presented (Fig. S1a, S2a, S3a, S4a, S5a, S6a, S7a, S8a, S9a). Maximum likelihood phylogenetic trees of different proteins with the highest degrees of similarity/identity to proteins predicted in yellow lupine were constructed (Fig. S1b, S2b, S3b, S4b, S5b, S6b, S7b, S8b, S9b). In all cases, the yellow lupine proteins showed the closest relationship to proteins derived from legumes, especially narrow-leaf lupine (*L. angustifolius*). To better understand the function of all yellow lupine proteins, the conserved domains, motifs and specific amino acids were discovered and localized (Fig. S11A’-F′; Fig. S12A’-C′). The positions of all conserved domains, motifs and amino acids present in yellow lupine were determined and confirmed using the background of similar proteins occurring in many plant species (Fig. S1c, S2c, S3c, S4c, S5c, S6c, S7c, S8c, S9c). Additionally, the predicted yellow lupine proteins were assigned a likely function based on those described in other plant species (Table 1). The tertiary structures of yellow lupine proteins were predicted using the Robetta service (Fig. S11A-F; Fig. S12A-C). Additionally, the amino acid sequences of yellow lupine were compared with those of other plant species. Both the similarity and the number of identical amino acids were included (Fig. S1d, S2d, S3d, S4d, S5d, S6d, S7d, S8d, S9d). It follows that proteins such as CAD, CesA8, COBL4 or GAUT12 are strictly conserved among the plant kingdom, while the remaining proteins (PG/QRT2, PCS1, GA3ox, GA2ox and GAMYB) show a greater affinity among closely related species but much lower identity/similarity with species as *A. thaliana*. The complete cDNA sequence of *Ll-MIR159* was identified in yellow lupine (Fig. S10) and compared with *MIR159* sequences cloned in other plant species that showed the highest similarity. On this basis, a phylogenetic tree was constructed (Fig. 7A). Further *Ll-MIR159* analyses revealed very high similarity of the 21-nt fragment that forms a mature miRNA to another identified in different plant species. In 11 of the 12 species compared, these fragments of sequences were 100% identical (Fig. 7B). The fragment of secondary stem-loop structure of Ll-pre-miR159 and localization of mature miR159 on the stem of the precursor sequence is presented (Fig. 7C). The alignment of the nucleotide sequences of Ll-pre-miR159 with mature miR159 in *Manihot esculenta*, *Dimocarpus longan*, *Populus trichocarpa*, *Populus tomentosa*, *Cucumis melo*, *Citrus sinensis* and *A. thaliana* is shown (Fig. 7D). The alignment of some nucleotide sequences of *LlGAMYB* (Ll-miR159 target gene) with homologous genes in *Glycine soja*, *G. max* and *A. thaliana* is also presented (Fig. 7E).

**Table 1:** Predicted function of conserved domains, motifs and specific amino acids in yellow lupine proteins [LlCAD (cinnamyl alcohol dehydrogenase); LlCesA8/LlIRX1 (cellulose synthase A catalytic subunit 8/IRREGULAR XYLEM1); LlCOBL4/IRX6 (COBRA-like4); LlGAUT12/LlIRX8 (galacturonosyltransferase12); LlPG/LlQRT2 (polygalacturonase/ QUARTET2); LlPCS1 (PROMOTION OF CELL SURVIVAL1); LlGA3ox (gibberellin 3-oxidase); LlGA2ox1 (gibberellin 2-oxidase1); LlGAMYB)] based on data published in other plant species

Protein	Identified conserved domains/motifs/specific amino acids	Predicted functions
LlCAD	Alcohol dehydrogenase GroES-like domain	Catalytic domain with GroES-like structure [38]
	Zinc-binding dehydrogenase domain	Catalytic activity, zinc ion binding [38]
	Zn-1 (GHExVGxVxxxGxxV) and Zn-2 (GxxVGxGxxxxxCxxCxxCxxxxxxxC) binding motifs	Zn-1 catalytic centre and Zn-2 binding site [39]
	Three amino acids C, H, C	Define places of catalytic Zn action [40]
	Four C residues	Structural Zn ligation (Zn-2 structural motif) [40]
	G residues (GxGGxG) (so-called Rossmann fold) represent NADPH co-substrate-binding motif	G residues for substrate specificity [40]
	S 212	Specific NADP(H) binding residue [40]
	Many conserved residues: S, Q, L, M, W, V, P, L, F, I	Determine substrate ligation [38]
LlCesA8/ LlIRX1	N-terminal region inclusive of a Zn-binding RING motif with a strictly conserved CxxC sequence motif beginning amino acids: CxxCx_12_FxACxxCxxPxCxxCxExxxxxDxxxCxxC	Protein-protein interactions in the CesA complex [41–43]
	Hypervariable region (VR1) of 117 aa, rich in acidic aa	This region is more conserved than was previously thought. The contribution of this region to the overall function of the enzyme is unknown [41, 43]
	Two transmembrane domains near the N-terminus (TMH1–2) and six transmembrane domains (TMH3–8) at the C-terminus	Transmembrane helixes [44]
	Large cytosolic/catalytic central domain (CD = globular domain = soluble domain), which includes the Plant Conserved Region (P-CR) within Conserved Region 1 (CR1), Class Specific Region (CSR) within Variable Region 2 (VR2) and Conserved region 2 (CR2)	[41–43]
	Located in the CD domain A consists of several widely spaced aspartic acid (D) residues - a single D followed by a DxD	These residues bind the UDP-glucose substrate. Processive enzymes catalyse the addition of many sugar residues to a growing chain [41, 43]
	Located in the CD domain B consists of a third conserved aspartic acid (D) residue and three conserved amino acids QxxRW	Part of the catalytic site [41, 43]
LlCOBL4/ LlIRX6	The putative conserved domain characteristic to COBRA superfamily	CDD (NCBI)
	N-terminal signal peptide with cleavage site	Signal peptide cleavage site [45, 46]
	The putative cellulose-binding site	A carbohydrate-binding module (CBM) [46]
	The central Cys-rich (CCVS) motif	Highly conserved and characteristic for all COBL proteins [45, 46]
	Two conserved consensus N-glycosylation sites	Asparagine (N)-linked glycosylation of protein [46]
	Locus corresponds to the predicted cleavage ω-site at the C-terminus	Glycosylphosphatidylinositol (GPI) modification motif. GPI anchors are added through an amide bond onto the last amino acid residue remaining after cleavage of the ω-site [46]
LlGAUT12/LlIRX8	N-terminal cytoplasmic domain	Phyre^2^
	The transmembrane domain	Phyre^2^
	The specific glycosyl transferase family 8 (GT8) domain	Transfer sugar residues to donor molecules. CDD (NCBI)
	The catalytic DxD motif	CDD (NCBI)
LlPG/ LlQRT	Four typical conserved domains I, II, III and IV	The well-conserved positively charged domain IV (RIKT) constitutes a likely candidate for ionic interactions with carboxylate groups present in the substrate [47–49]
	Three aspartic acids (D) in domains I and II	The carboxylate group in aspartic acids in NT**D** and **DD** structures (domains I and II, respectively) may be a component of the catalytic site [50]
	The histidine residue (H) in domain III	Participates in catalytic reaction [51]
	A tyrosine (Y) at position 320	Catalytically important in PGs [52]
	12 cysteine (C) residues	Important to maintain the three-dimensional structure of extracellular proteins and are distributed all along the sequences but with a higher frequency at the C-terminal end [49]
LlPCS1	Two motifs in both N (DTGS) and C (DS/LGT)-terminal ends characteristic for pepsin like aspartic proteases	Catalytic motifs (CDD, NCBI)
	Two catalytic residues (D)	Plays key catalytic roles in the pepsin family and conserved for all family members (CDD, NCBI)
	Active site flap ATLS and SSSS	An extended loop projecting over the cleft to form an 11-residue flap, which encloses substrates or inhibitors within the active site. It also contributes three residues for substrate specificity (CDD, NCBI)
	Pepsin A like plant domain	Characteristic for chloroplast nucleoids DNA-binding protease and nucellin, pepsin-like aspartic proteases (CDD, NCBI)
	TAXi_N domain; TAXI_C domain Xylanase inhibitor	The N- and C-termini of the members of this family are jointly necessary for creating the catalytic pocket necessary for cleaving xylanase (cell-survival processes) (CDD, NCBI)
LlGA3ox	Gibberellin 3-β-dioxygenase domain	CDD (NCBI) [53]
	2-oxoglutarate (2OG) and Fe (II)-dependent oxygenase (Oxy) superfamily domain	CDD (NCBI)
	The His-x-Asp-(x)n-His (HxD … H) and Arg-x-Ser (RxS) motifs	Recruit Fe (II) as a cofactor and co-substrate CDD (NCBI)
LlGA2ox1	Gibberellin 2-β-dioxygenase domain	CDD (NCBI)
	Domain characteristic for 2-oxoglutarate (2OG)-Fe (II)-dependent oxygenase superfamily	CDD (NCBI)
	The HxD … H and RxS motifs	Amino acid residues presumed to bind Fe^2+^ at the active site of protein
LlGAMYB	R2R3 domain	Near the 5′ terminus
	Box 1, Box 2, Box 3 domains	Distributed throughout the protein
	REB1 domain	Characteristic for Myb superfamily proteins, including transcription factors and mRNA splicing factors
	Myb_DNA-binding domain and SANT (SWI3, ADA2, N-CoR and TFIIIB’) domains	DNA-binding domains have been designated using CDD (NCBI)

## Discussion

### Changes in anther structure of cleistogamous yellow lupine

The dehiscence of anthers is a multistage process that has been examined in species such as *A. thaliana*, rice, maize (*Zea mays*), tobacco and tomato (*Solanum lycopersicum*) [4]. In our previous study, we showed that yellow lupine anther opening takes place in the fourth stage of flower development [34]. Therefore, it is relevant to study the process of late anther development and accompanying alterations in the tested cleistogamous species. In this paper, we established that the anther wall consists of epidermis, endothecium, middle layers and a tapetum, similar to its composition in other plant species, including both cleistogamous species, such as barley and wheat (*Triticum* L.), and chasmogamous species, such as tomato and rice [10, 54–56]. The yellow lupine epidermis protects all anther tissues and develops the stomium, which acts as the site for anther opening, whereas modifications of the endothecium play a critical role in pollen release. The other anther tissues in yellow lupine act similarly to several plant species; hence, it follows that the anther dehiscence process is widely preserved [1, 4, 8, 10, 54, 57]. Nevertheless, there are certain exceptions [12]. Unlike yellow lupine, where the processes of stomium breakage and anther opening are connected in time, in some species such as *Allium triquetrum*, these processes are separated. After the rupture of both stomium simultaneously, each *A. triquetrum* whorl does not fully open, and pollen is not released due to unbroken epidermal cells [58]. Legumes are one of the largest families of cleistogamous plants, although there are different types of cleistogamy [59]. Lupine species show often pre-anthesis cleistogamy when pollination occurs during the bud stage, but the flower ultimately opens. Most annual lupine species reproduce via self-pollination, but cross-pollination is also possible to a lesser degree [60]. In the case of peanut (*Arachis hypogaea*), there is dimorphic/true cleistogamy consisting of flower dimorphism and different developmental pathways within one individual or species [59, 61]. There are also species with only cleistogamous flowers (complete cleistogamy), including many orchids and grasses [59].

### The yellow lupine anther endothecium undergoes lignocellulosic deposition leading to secondary thickening

The secondary thickening of endothecial cell walls occurs in four basic forms depending on the species: (I) annular ribs, (II) helical ribs forming a pattern of U-shaped thickening, (III) reticulate ribs and (IV) palmate ribs [4, 11]. Some species may have two types of thickenings, but the walls of the endothecium typically show only one type [11]. For example, in *A. triquetrum* there are helical and U-shaped thickening whereas in *Crocus* (Iridaceae) species the thickenings are modified in the form of repeated continuous rings [58, 62]. The location and shape of the endothecium can also vary in a species-specific manner. In yellow lupine, the endothecium partially surrounds the locule; the endothecium is located in the upper third of the anther in tomato [54] and the endothecium adheres circularly to the locule in maize [4, 63]. Additionally, in yellow lupine, secondary thickenings are mainly found in endothecial cells (much less connective) whereas in certain species, secondary thickenings are also found in other types of anther cells. *A. triquetrum* has thickenings in the endothecium as well as in the cells of the septum and the connective tissue surrounding the locules [58]. In some species, the thickening epidermis may perform a function similar to that of the endothecium, thereby providing mechanical force to support anther opening [64]. It is also known that dehiscence mechanisms can be variable, both among families and within a given family. The opening of the anther may be caused via cell lysis and/or mechanical force; with the aid of secondary cell wall thickening and desiccation, the stomium ruptures, releasing pollen grains.

The presence of lignin in the endothecium layer determines the formation of secondary cell wall thickening. In most species, the CAD enzyme is involved in the biosynthesis of lignin monomers, and converts cinnamyl aldehydes into their corresponding alcohols. The *A. thaliana* mutant lacking *CAD* displays male sterility and, compared with wild-type (WT) plants, the lignin content is reduced and the lignin structure is greatly disrupted [14]. These changes cause a sterile mutant due to the lack of lignification of the endothecium, which does not result in secondary thickenings and hence does not release pollen. In our paper, we examined the transcriptional activity of *LlCAD* during the late development of yellow lupine anthers. The highest level of *LlCAD* expression was found in the first and second phases during which the process of endothecial cell wall lignification take place. Approximately 20 times more gene transcripts were found compared to the third and fourth LAD stages. This suggests that *LlCAD* participates in lignin biosynthesis in the tissues of yellow lupine anthers.

In addition to lignification of the endothecium, cellulosic thickening is also essential for anther dehiscence. The mutation of the *A. thaliana CesA8*/*IRX1* gene responsible for the synthesis of cellulose, does not affect the formation of primary cell wall thickening, which allows for us to use this gene as a specific marker for secondary thickening [15, 16]. In yellow lupine, the expression of *LlIRX1*/*LlCesA8* was very high in the first two stages of LAD. This closely correlates with the transcriptional activity of *LlCAD* and other identified genes - *LlIRX6*/*LlCOBL4* and *LlIRX8*/*LlGAUT12*. In general, *COBL* genes, which encode plant-specific glycosylphosphatidylinositol (GPI) anchored proteins, are key regulators in the deposition of cellulose in the secondary cell wall [65]. In turn, xylan- and pectin-deficient *irx8* has a mutated *GAUT12* gene, the loss of which results in reduction of G lignin, changes in its deposition, and a lack of anther splitting [18].

Different phytohormones control the formation and deposition of secondary thickening in many plant species. For example, cytokinins (CKs) have been implicated in the regulation of secondary wall formation, since AHP4 (Arabidopsis histidine-containing phosphotransfer 4), which is an element of CK signalling, negatively regulates thickening in the endothecium [66]. Additionally, the transcriptional activity of *AHP4* corresponds to *IRX1*/*IRX6*/*IRX8* expression, which indicates that CKs control cellulose biosynthesis [4, 66]. To date, little is known about the effect of GAs on the formation of secondary thickening; therefore, it became the subject of our research. The impact of this phytohormone treatment on the expression of all secondary thickening-related genes was very similar and consisted of increasing the number of transcripts, especially in the second and third LAD phases. In *Cucumis melo*, several hormone-responsive cis-regulatory elements in the *CAD* promoter region were identified, including GAREs, TATC boxes and P-boxes characteristic of GAs [40]. It also confirms the important role played by GAs in the comprehensive progression leading to anther opening.

### The septum/stomium is enzymatically lysed and undergoes PCD-mediated degeneration

In yellow lupine anthers, separation of the septum from the stomium cells seems to occur by a mechanism similar to the organ abscission process, which involves enzymatic lysis of the middle lamellae between the cells without their damage [35]. In our study, the expression profile of the *LlQRT2* gene encoding a marker enzyme involved in pectin breakdown between cells during LAD in yellow lupine was investigated. The transcript level was highest when the septum and stomium split (first and second LAD phases, respectively). If the dissociation is mechanical, e.g., by stretching of expanding anther walls, it damages the cells involved [62]. Ogawa et al. (2009) have shown that the regulation of cell separation events involves combinations of PGs and phytohormones [19]. *A. thaliana* and tomato *pg* mutants show delayed or blocked anther dehiscence; hence, it is known that the role of cell wall enzymatic lysis is important [19, 67]. Additionally, the transcriptional activity of *A. thaliana PG* - *QRT2* is regulated by JA, ET and ABA [19]. In yellow lupine, it has been revealed that GA_3_ and the ET precursor regulate the functioning of the flower abscission zone; however, GA_3_ acts independently of ABA in this process [33]. In this paper, we demonstrated that the *LlQRT2* transcript level was positively controlled by GA_3_. The level of *LlQRT2* mRNA increased, especially in the second LAD phase, which suggests GA-dependent modulation of the septum/stomium interruption in the studied plant. Data on the impact of GAs on *PG* expression in different plant species are lacking. In *A. thaliana* anthers, the application of another phytohormone, JA, causes an increase in *QRT2*, *ADPG1* (*ARABIDOPSIS DEHISCENCE ZONE POLYGALACTURONASE1*) and *ADPG2* expression by approximately 10-fold [19]. In wheat (*T. aestivum*), cis-acting elements in the promotor sequences of *TaPG* genes were predicted [68]. Importantly, they included cis-elements regulated by phytohormones and transcription factors such as abscisic acid (ABA; ABRE), GAs (P-Box), auxin (TGA-element), methyl jasmonate (MeJA; CGTCA, TGACG), and MYB binding sites involved in drought inducibility (MBS). Similar research was carried out in *Brassica oleracea*, and hormone-response cis-elements were found for MeJA (CGTCA), auxin (TGA-box and AuxRR-core), GA (GARE, P-box, TATC-box), salicylic acid (TCA-element), and ABA (ABRE) [69].

The separated septum cells of yellow lupine progressively degenerate. The septum degenerates first to create a bilocular anther, and the stomial cells then degenerate to break the anther wall. In other plant species, these processes occurring in specified cells are aided by PCD [1, 8]. Ge et al. (2005) showed that the PCD inhibitory factor gene *PCS1*, encoding aspartic protease, promotes cell survival during embryogenesis and gametogenesis. *PCS1* is not continuously expressed in tissues but is involved in various specific developmental processes. A characteristic pattern of *PCS1* expression has been demonstrated in developing flowers, young siliques, and anthers. Further analyses revealed the presence of *PCS1* mRNA in pollen and the anther wall, with the exception of the tapetum layer, where no information has been provided [21]. The overexpression of *PCS1* in *A. thaliana* anthers results in the inhibition of the dehiscence process; therefore, we focused on establishing the expression of this gene in yellow lupine tissues. Our results show that the level of *LlPCS1* fluctuates at different stages of LAD. It is extremely interesting that *LlPCS1* transcriptional activity is controlled by GA_3_. As expected, hormone molecules reduced the amount of *LlPCS1* mRNA, especially in the first and fourth LAD phases, which indirectly proves the GA-dependence of septum/stomium cell degeneration in yellow lupine. In summary, the results suggest that PCD-related processes occur during the development of yellow lupine anthers, but the mechanisms of these strictly controlled processes require further research. In various plant species, two main types of stomium rupture were recognized, basic mechanical breakage and active PCD-related degeneration [8, 9, 70]. Developmental PCD is controlled by phytohormones. Sanders et al. (2000) showed that the mutant associated with the JA biosynthetic pathway *opr3* is characterized by a delay in the degeneration of stomium cells, which disturbs anther dehiscence [9]. Thus, there is strong indirect proof of the involvement of these phytohormones in PCD and in the process of anther opening.

### Gibberellins are modulators of anther dehiscence in yellow lupine

The multifunctionality of GAs in the early stages of anther development, mainly tapetum and pollen, is well documented through analyses of *A. thaliana* and rice mutants. However, the late stages of anther development related to their final split are not well understood due to the earlier GA-deficiency mutant disturbances that prevent further anther development [71]. In yellow lupine, expression profile studies have shown that fluctuating transcript levels of *LlDELLA1*, which encodes the main repressor of GA signalling, promote proper flower and pod development. The *LlDELLA1* mRNA level is lowest when the anthers are opened and then increases during the fertilization and early pod developmental stages [34]. This encouraged us to investigate the localization of GA_3_ in selected stages of late anther development in yellow lupine. Our results obtained in this work closely correlate with the expression pattern of the *LlDELLA1* gene, in the opposite manner. The highest accumulation of GA_3_ occurs in the first LAD phase, which is before anther opening. The GA_3_ signal was observed mainly in degenerating septum cells, near the vascular bundle, in the middle layer, endothecium, and less frequently in the epidermis. In the second LAD phase, where the stomium cells were disrupted and the pollen chambers were opened, the fluorescence signal was almost unnoticeable, and no signal was detected in the third and fourth LAD stages. In the case of rice, GA_4_ accumulation was found to be highest in anthers just before anthesis. The level of active hormone molecules decreased, and was completely undetectable in the young seeds a week after anthesis [72]. These results suggest that GAs play an important role in specific organs (place) at a specific stage of the life cycle (time) and that they may comprise the strict regulation of reproductive growth and development in different species.

The application of the GA biosynthesis inhibitor PAC in petunia (*Petunia hybrida*) causes male sterility resulting from the inhibition of anther development in the postmeiotic phase. Detailed analyses showed that the connective cells and tapetum were degenerated, but pollen grains were still present. In petunia transgenic plants overexpressing the GA signalling repressor *AtSPY*, the anther phenotype was comparable to that observed after PAC treatment [73]. In some plants, inhibition of GA biosynthesis blocks anther development after meiosis (petunia, *A. thaliana*, maize), but in others, blockage occurs before meiosis, i.e., tomato [71]. The key and final gene involved in GA biosynthesis is *GA3ox*. We showed that the transcriptional activity of *LlGA3ox* strictly correlates with GA_3_ localization in yellow lupine during late anther development. The most *LlGA3ox* transcripts were found in the first and second LAD stages, and the level decreased significantly in the third and fourth stages. Moreover, after the application of PAC, the amount of *LlGA3ox* mRNA decreased by more than half, mainly in the first three selected phases. This suggests that this particular *LlGA3ox* gene may be involved in the synthesis of bioactive phytohormone molecules that play an essential role in the studied process in yellow lupine. In addition, we demonstrated that the expression of *LlGA2ox1*, encoding the enzyme responsible for GA inactivation, shows the opposite expression to that of *LlGA3ox*. The role of genes involved in GA biosynthesis and inactivation during stamen development is well understood in many plant species. The transcriptional activity of *AtGA3ox1-AtGA3ox4* genes was observed in stamens, and *AtGA3ox1* was also expressed in some other floral organs. Both the stamen filaments and anthers require GAs for proper development, and de novo synthesis of this hormone was confirmed at both sites. The transcript of *AtGA3ox1* was found in the filament, and *AtGA3ox2-AtGA3ox4* genes were expressed in anthers and pollen [26]. GAs, mainly GA_4_, need to be transported from stamens or flower receptacles to other floral organs to ensure proper development. Despite the lack of expression of all *AtGA3ox* genes in the petals, the mutant deficient in *AtGA3ox1* and *AtGA3ox3* showed serious defects in petal development [26]. In yellow lupine anthers, this could be explained by the observation of a fluorescent signal indicating the presence of GA_3_ in the cells of the conductive bundle. Therefore, there is a high probability that the active phytohormone molecules are transported from the anther to the filament or vice versa. An analogous situation was observed in petunia, where GA application restored the normal corolla pattern, previously disturbed by removal of the stamens [74]. Similarly, anther expression patterns of *GA3ox* genes were also observed in tobacco and rice [75, 76]. In summary, stamens are a key source of GAs in flowers in different plant species. In addition to their local influence on stamen development, their transport to adjacent tissues of flower organs is equally important [26].

The GAMYB transcription factor mediates GA signal transduction in the aleurone tissues of cereals. Furthermore, it is necessary to ensure the fertility of anthers in many species. Similar to GA biosynthesis mutants, *gamyb* demonstrates the impact of GA signalling on the early stages of anther development. A mutation in *GAMYB* in rice and the double mutation of *MYB33* and *MYB65* in *A. thaliana* interrupt PCD, causing abnormal enlargement of tapetum cells, which disorders pollen development [28]. However, there are few data concerning the role of *GAMYB* in late anther development. In this paper, we examined yellow lupine *LlGAMYB* expression in selected phases of LAD and after GA_3_ and PAC application. The *LlGAMYB* transcript level was almost identical with or without GA_3_ treatment, but PAC application resulted in increased *LlGAMYb* mRNA, especially in the first and second LAD phases. This suggests that the GA_3_dependent pathway may control *LlGAMYB* expression but not in a direct manner. It can be assumed only that the presence of GA_3_ blocks the transcription factor, which positively regulates the expression of *LlGAMYB*, but the exact mechanism requires further research. The results obtained in other plant species show that *HvGAMYB* in barley and *GAMYB-like* genes in *A. thaliana* are expressed in flowers and are positively regulated by GAs [29, 77]. It follows that the role of *GAMYB* in GA-regulated gene expression during anther development differs between species. Moreover, the function of *GAMYB* also varies within a species between developmental processes, e.g., seed germination and vegetative and reproductive development [78]. Interestingly, the results of studies carried out on barley, indicate that transgenic plants strongly overexpressing *HvGAMYB* in anthers are completely male sterile. This phenotype was characterized by an intact septum [29]. Taking into account our results obtained in yellow lupine, where we observed the highest level of GA_3_ in progressively degenerating septum cells, it can be concluded that the presence of GA_3_ at this stage of anther development strictly regulates the level of target gene transcripts (establishing a certain balance), which results in a specific and correct physiological response. Pollen of *HvGAMYB*-overexpressing barley anthers has a specific phenotype [29]. The development of slightly smaller and irregular pollen is normal, and some pollen has been shown to be viable. The observed male sterility is mainly due to disrupted anther wall tissues, which do not break [29]. Similar to barley, a phenotype of small, indehiscent anthers was observed in wheat after GA_3_ treatment [79, 80]. The occurrence of male sterility following the application of GA_3_ in wheat, in conjunction with the observations that (1) *HvGAMYB* overexpression causes anther indehiscence and that (2) *LlGAMYB* expression is relatively constant after GA_3_ application but increases after PAC treatment, suggest that *GAMYB* plays a specific role during anther development in a species-specific manner. This requires further research and more clarification.

Many reports have suggested that the expression of *GAMYB-like* genes in *A. thaliana* is regulated posttranscriptionally by a microRNA (miRNA) named miR159 [30, 81]. miR159 is a small (21 nt), noncoding RNA sequence derived from double-stranded RNA (dsRNA). At-miR159 affects the transcriptional activity of its target genes, including *AtMYB33*, *AtMYB65* and *AtMYB101* [30]. In rice, Tsuji et al. (2006) revealed that miR159 regulates the transcript level of *GAMYB* only in flowers, and this situation was not observed in aleurone cells. It follows that the regulation of *GAMYB* expression and its function is organ specific [78]. Due to this fact, and also to find a factor that may determine the level of *LlGAMYB* transcripts during anther development in yellow lupine, we examined *LlMIR159* expression after GA_3_ and PAC application. Interestingly, GA_3_ treatment increased the *LlMIR159* transcript content, and PAC application significantly decreased it. The observed expression profile of *LlMIR159* partially explains the transcriptional activity changes observed for *LlGAMYB* after different compound applications. The results also indicate that the expression of the *LlMIR159* gene is regulated via the GA signalling pathway. A similar situation occurs in *A. thaliana*, where GAs regulate miR159 levels [30]. In the flowers of GA biosynthesis mutant *ga1–3*, there was significantly less miR159 than in the WT flowers. Application of GAs to the flowers of the mutant increased the level of miR159 to a level comparable to that of GA-treated WT plants, and this level was higher than that of untreated WT plants. Due to the reduced level of *AtMYB33*, plants overexpressing miR159 showed male sterility and delayed flowering [30]. As demonstrated by experiments with the *GUS* reporter gene, the localization of AtMYB33:GUS encoded by a gene with the complete miR159 target sequence was only in young anthers whereas AtMYB33:GUS was expressed in various flower organs when the gene had damage to the miR159 target sequence [81].

By analysing the natural conditions without the application of any compounds, it can be concluded that *LlGAMYB* expression is high in the first two stages of LAD and then decreases. This negatively correlates with the transcriptional activity of *LlMIR159*, which was significantly increased in the second phase of LAD. This is probably the cause of the reduced mRNA content of *LlGAMYB* in the third and fourth LAD stages. The results obtained suggest that *LlGAMYB* is coexpressed with *LlMIR159* in yellow lupine anthers. It also follows that the *LlGAMYB* gene is regulated by miR159 in the anther of yellow lupine.

### In silico analyses

In this paper, nine full-length cDNA sequences of genes related to the anther dehiscence process in yellow lupine were identified. The implemented methodology was successful for the identification and characterization of the conserved domains, motifs and specific amino acids of all predicted proteins. This approach made it possible to predict and assign them specific functions. The identified genes encode functional proteins closely related to the Fabaceae family. Moreover, multiple protein alignments provided information about the conservation of sequences in many plant species. It also allowed for the specification of fragments of protein sequences that are the crucial for the function they perform. The construction of phylogenetic trees allowed for making conclusions about the origin of the predicted yellow lupine proteins.

Based on the available bioinformatics and experimental results, miR159 mediates the regulation of the family of *GAMYB* and *GAMYB-like* genes in various plant species, including *A. thaliana* [81], rice [78] and strawberry [82]. These *GAMYB* genes from different plant species and *LlGAMYB* identified in this work from yellow lupine contain a conserved miR159 binding site, which is highly complementary to the corresponding miR159 sequence and is located in the conserved region between Box 1 and Box 2 of *GAMYB* genes [78, 81]. This indicates that the miR159-*GAMYB* pathway is conserved across species.

## Conclusions

This paper advances knowledge of the hormonal and molecular regulation of late anther development in the cleistogamous plant yellow lupine (*Lupinus luteus* L); such knowledge is an important aspect of controlling fertility in this valuable legume crop species. To date, there are no data showing the effect of GAs on the transcriptional activity of genes associated with deposition of lignocellulosic secondary thickening in the endothecium, enzymatic breakdown of cell walls at the septum/stomium and cell degeneration via PCD-related processes. Therefore, we showed that the temporal expression of the identified genes, as markers of developmental changes during yellow lupine anther dehiscence, is regulated in a GA-dependent pathway. Additionally, tissue and cellular localization suggests that GA_3_ is a modulator of this process, especially in the time prior to anther opening. The appropriate GA_3_ level, which correlates with GA metabolism, at the right time and place controls yellow lupine anther opening, likely through the influence on *LlGAMYB* expression via the *Ll-MIR159*-dependent pathway. Therefore, yellow lupine anther dehiscence is highly regulated, enabling the timing of pollen release to be tightly controlled to maximize the chances of fertilization.

## Methods

### Plant material, growing conditions and compound treatments

The plant material was yellow lupine (*Lupinus luteus* L.) cv. Taper. Seeds obtained from the Plant Breeding Station Wiatrowo (Poland) were prepared and sown as described in previous research [34] in the experimental field in Pędzewo, Kuyavian-Pomeranian Voivodeship in north-central Poland (53°05′02″N 18°21′28″E). The plants were cultivated in soil of the 5th class (poor arable soil; Polish bonitation classification of soils) as recommended by the manufacturer [83]. The anthers from particular stages of late development (1–4 LAD, Fig. 1A) were dissected from flowers with a sharp scalpel. For each phase of LAD, no less than 80 plants were used, and part of the collected material was previously treated with gibberellic acid (GA_3_, 100 μM) or GA biosynthesis inhibitor paclobutrazol (PAC, 100 μM) in a 0.05% Tween 20 solution using a sprayer. The anthers in the corresponding developmental phases were used as the control group, and were treated with 0.05% Tween 20 solution only. According to the planned experiment, 3 h after application, the anthers were harvested and processed fresh or frozen in liquid nitrogen (and stored at − 80 °C until use).

### Identification of cDNAs

The cDNA sequences of individual genes were identified differently. Molecular cloning of *LlGA2ox1* cDNA was performed as described in prior research [33]. The identification of *LlGAMYB* cDNA was as follows: 1 g of yellowish-green flowers was pounded in liquid nitrogen using a mortar and pestle. Then total RNA was isolated by the column method according to the manufacturer’s instructions (NucleoSpin® RNA kit, Macherey-Nagel, Düren, Germany), and reverse transcription was performed using 1 μg of RNA, oligo (dT)_18_ primers, and the Transcriptor High Fidelity cDNA Synthesis Kit (Roche, Mannheim, Germany). Touchdown PCR was performed using 1.25 U Perpetual Taq DNA Polymerase^HOT START^ (EURx, Gdańsk, Poland), 2 μl of first-strand cDNA, 1x buffer B, 0.2 mM dNTP mix, 3.0 mM Mg^2+^, 1 μM degenerated primers (Tab. S1) and deionized water (up to a final volume of 50 μl) in a T3 thermocycler (Biometra, Göttingen, Germany). An amplified cDNA fragment (704 bp, Fig. S13A) was isolated and purified from an agarose gel (GeneMATRIX Agarose Out DNA Purification Kit, EURx). At the insertion site, the PCR product was ligated into the pCRII-TOPO vector (Fig. S13B, TOPO TA Cloning Kit, Invitrogen, Carlsbad, USA) and transferred into One Shot Mach1-T1 *E. coli* in the form of a plasmid. The bacterial cells were plated on Petri dishes containing S-Gal/LB agar blend (Sigma-Aldrich, St. Louis, MO, USA) with ampicillin (50 μg/ml) (Fig. S13C). Unlike dark blue bacterial colonies, white colonies were selected and cultured in liquid LB medium with ampicillin (50 μg/ml) for 12 h. Finally, plasmid DNA was isolated in accordance with the manufacturer’s guidelines (GeneMATRIX Plasmid Miniprep DNA purification kit, EURx), digested with the restriction enzyme *Eco*RI (Fermentas) (Fig. S13D), and sequenced by Genomed (Warsaw, Poland). The 485 bp (Fig. S14A) and 768 bp (Fig. S14B) fragments were obtained from 3′ RACE-PCR (FirstChoice RLM-RACE Kit, SuperTaq-Plus Polymerase, Ambion, Austin, USA) using two different pairs of primers (Tab. S1). The amplicons were isolated, purified, cloned, digested (Fig. S14C/D) and sequenced as described above. Due to difficulties arising from the experimental identification of the 5′ end of *LlGAMYB* cDNA, it was obtained based on sequences derived from a later RNA-Seq experiment deposited at the NCBI in the Sequence Read Archive (SRA) database under accession number PRJNA285604 (BioProject) [84] and experiment accession number SRX1069734. All fragments had overlapping nucleotide sequences which allowed for us to obtain the complete *LlGAMYB* sequence. The de novo assembled transcriptome of yellow lupine from RNA-seq experiments was also used to identify *LlCAD*, all *LlIRXs*, *LlQRT2*, *LlPCS1*, *LlGA3ox1* and the precursor of Ll-miR159 (*Ll-MIR159*).

### Expression analysis

Quantitative real-time PCR (qPCR) was used to establish the expression pattern of all identified genes. Eighty milligrams of frozen anthers (at a specific stage of development with or without GA_3_/PAC treatment) were homogenized in a sterile mortar with a pestle. According to the manufacturer’s instructions, total RNA was isolated using an Isolate II RNA Plant Kit (Bioline, London, UK), and reverse transcription with matrix (2 μg), oligo (dT)_18_ (Roche) and the Transcriptor High Fidelity cDNA Synthesis Kit was performed. qPCRs were carried out using a LightCycler 2.0 Carousel-Based System (Roche) in 20 μl capillaries containing a mix of 0.1 μg of cDNA, 0.2 μM gene-specific primers (Tab. S1), 0.05 μM Universal Probe Library (UPL) hydrolysis probes (Roche) (Tab. S1), 1 × LightCycler TaqMan Master Mix (LightCycler TaqMan Master Kit, Roche) and H_2_O. The following program was used: 600 s at 96 °C; 45 cycles of 10 s at 96 °C, 20 s at a specific annealing temperature (Tab. S1), 1 s at 72 °C; and 30 s at 40 °C. Negative no template controls (NTCs) were included. As an endogenous control the actin gene (*LlACT*, GenBank accession number KP257588) was selected [32–34]. Absolute quantification was designed from the serial dilutions of cDNAs generating standard curves, and the relative gene expression (the data of the studied gene relative to the internal control gene and calibrator) was determined using the 2 (−DeltaDeltaC(T)) method [85].

### Histological studies

A fixer containing 4% paraformaldehyde (w/v), 0.2% glutaraldehyde (v/v) and 3% N-ethyl-N′-(3-dimethylaminopropyl) carbodiimide hydrochloride (EDAC) (w/v) (Sigma-Aldrich) in 1× phosphate-buffered saline buffer (1× PBS, pH 7.2) was prepared and applied to appropriate anther tissue small fragments for 12 h at 4 °C. Triplicate samples were washed of fixative with 1× PBS (pH 7.2) for 10 min. Dehydration was achieved by placing the material in increasing ethanol concentrations (30, 50, 70, 90, 100%) (v/v). Then, supersaturation and embedding in BMM resin (butyl methacrylate, methyl methacrylate, 0.5% (w/v) benzoin ethyl ether, 10 mM dithiothreitol) (Fluka, Buchs, Switzerland) were performed at − 20 °C under UV light. Using an ultramicrotome (Reichert-Jung, Germany) semithin sections were obtained, which were placed on slides, stained with 0.05% toluidine blue (Sigma-Aldrich), and observed under an LM Zeiss Axioplan (Carl Zeiss, Oberkochen, Germany) microscope with a ProGres C3 digital camera.

### GA_3_ immunolocalization

The anther tissue fragments were fixed, washed, dehydrated, supersaturated, embedded, polymerized and cut in the same way as described for the histological studies. Semithin sections were placed on slides with Biobond (BBInternational, Cardiff, UK), and after washing, the resin was blocked in BlockAid TM Blocking Solution (Thermo Fisher Scientific, Waltham, MA, USA) according to the manufacturer’s instructions. Then, sections were incubated with polyclonal primary antibody anti-GA_3_ (Abbexa, Cambridge, UK) diluted 1:50 in 1% bovine serum albumin (BSA) in 1x PBS (pH 7.2) and placed in a wet container at 4 °C for 12 h. Next, the primary antibodies were removed by washing 3 times in 1x PBS (pH 7.2), and secondary antibody (MFP488 goat anti-rabbit IgG, MoBiTec, Goettingen, Germany) diluted 1:250 in PBS buffer was applied for 2 h at 37 °C. A negative control was performed by omitting incubation with the primary antibody (Fig. S15). The final steps were incubation with DAPI (1:2500) for 10 min and washing with distilled water. The samples were observed under a Leica DMI4000B inverted microscope using BP365, FT395, and LP397 filters.

### Is silico analyses

The integrated FastPCR v.6.5.99 [86] tool was used to design the degenerate and RACE-PCR primers. The Universal Probe Library Assay Design Center [87] was used to design the qPCR-specific primers and short hydrolysis UPL probes substituted with locked nucleic acids. The identified cDNA sequences of all genes and predicted proteins were analysed using the Basic Local Alignment Search Tool (BLAST) [88] and the bioinformatics resource portal of the Swiss Institute of Bioinformatics [Expasy [89], including the Translate tool [90], which allows for the translation of nucleotide sequences to protein sequences, and the ProtParam tool [91], which allows the calculation of molecular weights and isoelectric points. Alignments and phylogenetic reconstructions were performed using the Python Environment for Tree Exploration3 (ETE3) v3.1.1 program as implemented in GenomeNet [92]. Maximum likelihood phylogenetic trees were inferred using PhyML v20160115 ran with model and parameters: --pinv e -o tlr -f m --bootstrap − 2 --nclasses 4 --alpha e. Branch supports are the Chi^2^-based parametric values return by the approximate likelihood ratio test. Multiple alignments of different amino acid sequences found in BlastP and showing close association with yellow lupine sequences were carried out using the ClustalW [93] program with the default settings. The presence of functional domains was checked via the NCBI Conserved Domain Database (CDD) [94], a protein annotation resource consisting of a collection of well-annotated multiple sequence alignment models for ancient domains and full-length proteins. These are available as position-specific score matrices (PSSMs) for the rapid identification of conserved domains in protein sequences via RPS-BLAST. The CDD content includes NCBI-curated domains as well as domain models imported from a number of external source databases (Pfam, SMART, COG, PRK, TIGRFAMs). The cytoplasmic and transmembrane domains were also predicted using TMHMM v. 2.0 [95] and the Protein Homology/analogY Recognition Engine V 2.0 (Phyre^2^) [96] web portal for protein modelling, prediction and analysis. Comparisons of proteins derived from different plant species were made using the DiAlign program (Genomatix) [97] with default parameters. The tertiary structures of the proteins derived from yellow lupine were constructed using the Robetta service for protein structure prediction [98], which uses the PDB100 template database and a coevolution-based model database (MDB). The results were visualized using UCSF ChimeraX [99], which is a next-generation molecular visualization program from the Resource for Biocomputing, Visualization, and Informatics (RBVI). The Ll-pre-miR159 sequence was analysed using the microRNA database (miRBase), ETE3 and BLAST. RNA structure software [100] was used to compute the secondary structure of Ll-pre-miR159.

### Statistical analysis

All presented data are the results of three separate samples (biological replications) with two repetitions of each (technical replications) and are presented as the mean ± standard error (SE). Statistical analysis was performed using one-way ANOVA followed by post hoc Tukey’s HSD test, with differences accepted at *p* < 0.05. All analyses were performed using R version 3.5.3.

## Supplementary Information

**Additional file 1: Fig. S1**a. *LlCAD* cDNA (GenBank accession number MW240676, 2250 bp) identified in yellow lupine (*Lupinus luteus* L.) and its deduced amino acid sequence [357 aa (ExPASy, translate tool), m.w. = 38.936 kD and pI = 6.01 (ExPASy, ProtParam)]. The nucleotides are marked with lowercase letters, and amino acids are marked with capital letters. The START and STOP codons (yellow background) are indicated. 5′ and 3′ UTR regions are marked with small italic letters before the ATG and after the TGA codons, respectively. The ORF is shown in pink. **Fig. S1**b. Maximum likelihood phylogenetic tree of 15 cinnamyl alcohol dehydrogenase (CAD) proteins (BlastP) with the highest degree of similarity to LlCAD. Alignment and phylogenetic reconstructions were performed using the Environment for Tree Exploration3 (ETE3) v3.1.1 program as implemented in GenomeNet. The ML tree was inferred using PhyML v20160115. Branch supports are the Chi^2^-based parametric values returned by the approximate likelihood ratio test. **Fig. S1**c. Multiple alignment (ClustalW) of 15 cinnamyl alcohol dehydrogenase (CAD) amino acid sequences (BlastP) that are closely related to LlCAD. The alcohol dehydrogenease GroES-like domain is shaded medium grey, and the zinc-binding dehydrogenase domain is shaded dark grey. The Zn-1 and Zn-2 binding motifs are shaded with violet. Three green amino acids, C, H, and C, are marked with green dots. Yellow letters define four cysteine (C) residues. Conserved glycine (G) residues (GxGGxG) are indicated with red letters. They represent NADPH cosubstrate-binding motif, which is highlighted in green. The conserved residues (S, Q, L, M, W, V, P, L, F, I) are highlighted in blue. The serine (S) 212 is labelled with a red background. Most of the alignment information was identified according to the results described by [38–40]. *Lang* - *Lupinus angustifolius* (XP_019452456); *Lalb* - *Lupinus albus* (KAE9589372); *Ap* - *Abrus precatorius* (XP_027363622); *Vu* - *Vigna unguiculata* (XP_027937462); *Gs* - *Glycine soja* (XP_028185029); *Gm* - *Glycine max* (XP_003555961); *Ss* - *Spatholobus suberectus* (TKY50969); *Ck* - *Caragana korshinskii* (AEV93476); *Ah* - *Arachis hypogaea* (XP_025620500); *Ad* - *Arachis duranensis* (XP_015941997); *Ai* - *Arachis ipaensis* (XP_016175492); *In* - *Ipomoea nil* (XP_019169368); *Mt* - *Medicago truncatula* (XP_013470061); *Cc* - *Cajanus cajan* (XP_020210039); *At* - *Arabidopsis thaliana* (AAK44076). **Fig. S1**d. Comparison of CAD proteins derived from different plant species (BlastP) using the DiAlign program (Genomatix). For each pairwise alignment, the similarity (relative to the maximum similarity) and the number of identical amino acids (in % of shorter sequence) are given. Maximum values are underlined. The similarity value of 1.000 denotes only the two most similar sequences; it does not necessarily mean that these sequences are identical. *Lang* - *Lupinus angustifolius* (XP_019452456); *Lalb* - *Lupinus albus* (KAE9589372); *Ap* - *Abrus precatorius* (XP_027363622); *Vu* - *Vigna unguiculata* (XP_027937462); *Gs* - *Glycine soja* (XP_028185029); *Gm* - *Glycine max* (XP_003555961); *Ss* - *Spatholobus suberectus* (TKY50969); *Ck* - *Caragana korshinskii* (AEV93476); *Ah* - *Arachis hypogaea* (XP_025620500); *Ad* - *Arachis duranensis* (XP_015941997); *Ai* - *Arachis ipaensis* (XP_016175492); *In* - *Ipomoea nil* (XP_019169368); *Mt* - *Medicago truncatula* (XP_013470061); *Cc* - *Cajanus cajan* (XP_020210039); *At* - *Arabidopsis thaliana* (AAK44076). **Fig. S2**a. *LlCesA8* (alternative name *LlIRX1)* cDNA (GenBank accession number MW240677, 3788 bp) identified in yellow lupine (*Lupinus luteus* L.) and its deduced amino acid sequence [975 aa (ExPASy, translate tool), m.w. = 110.227 kD and pI = 5.97 (ExPASy, ProtParam)]. The nucleotides are marked with lowercase letters, and amino acids with capital letters. The START and STOP codons (yellow background) are indicated. 5′ and 3′ UTR regions are marked with small italic letters before the ATG and after the TAA codons, respectively. The ORF is shown in pink. **Fig. S2**b. Maximum likelihood phylogenetic tree of 12 CesA8 proteins (alternative name IRX1, BlastP) with the highest degree of similarity to LlCesA8/LlIRX1. Alignment and phylogenetic reconstructions were performed using the ETE3 v3.1.1 program as implemented in GenomeNet. The ML tree was inferred using PhyML v20160115. Branch supports are the Chi^2^-based parametric values returned by the approximate likelihood ratio test. **Fig. S2**c. Multiple alignment (ClustalW) of 12 CesA8/IRX1 amino acid sequences (BlastP) that are closely related to LlCesA8/LlIRX8. Proteins contain N-terminus; globular/soluble central domain (CD) and the C-terminus. Short N-terminus (N) is prior to the Zinc-binding domain (Zn) (medium gray). This Zn domain contains strictly conserved CxxC motif (green box and red letters) beginning amino acids: CxxCx_12_FxACxxCxxPxCxxCxExxxxxDxxxCxxC, where x is any amino acid. Within the N-terminus is also a region rich in acidic amino acids designated as a hypervariable region (VR1, red box). Following the N-terminal domains are two transmembrane domains (TMH1/2, yellow boxes). The CD contains variable region 2 (VR2, dark grey) composed mostly of the Class Specific Region (CSR) which on either side is flanked by conserved regions: CR1 [with the Plant Conserved Region (P-CR) in the middle highlighted in light grey)] and CR2. The four recognized catalytic motifs are marked as blue boxes, with the D, DxD, and D residues in bold red and the QxxRW residues in bold orange. The numerous basic residues of Arg (R) and Lys (K) (green leterrs) and acidic residues of Asp (D) and Glu (E) (red letters) of the VR2/CSR, as well as the conserved Cys (C, yellow letters) are conserved across species in an isoform-specific manner [43]. The C-terminus contains six transmembrane domains (THM 3–8) and remaining protein after the last TM helix marked as C. **Fig. S2**d. Comparison of CesA8 proteins derived from different plant species using the DiAlign program (Genomatix). For each pairwise alignment, the similarity (relative to the maximum similarity) and the number of identical amino acids (in % of shorter sequence) are given. Maximum values are underlined. The similarity value of 1.000 denotes only the two most similar sequences; it does not necessarily mean that these sequences are identical. *Lang* - *Lupinus angustifolius* (XP_019439632); *Gm* -*Glycine max* (XP_003526279); *Mt* - *Medicago truncatula* (XP_013462596); *Va* - *Vigna angularis* (XP_017421931); *Cc* - *Cajanus cajan* (XP_020211247); *Ap* - *Abrus precatorius* (XP_027339063); *Ad* - *Arachis duranensis* (XP_015943020); *Ah* - *Arachis hypogaea* (XP_025621820); *Gs* - *Glycine soja* (KHN48007); *Jc* - *Jatropha curcas* (XP_012091811); *Lalb* - *Lupinus albus* (KAE9602950); *At* - *Arabidopsis thaliana* (NP_567564). **Fig. S3**a. *LlCOBL4* (alternative name *LlIRX8)* cDNA (GenBank accession number MW240678, 2370 bp) identified in yellow lupine (*Lupinus luteus* L.) and its deduced amino acid sequence [431 aa (ExPASy, translate tool), m.w. = 48.489 kD and pI = 8.85 (ExPASy, ProtParam)]. The nucleotides are marked with lowercase letters, and amino acids with capital letters. The START and STOP codons (yellow background) are indicated. 5′ and 3′ UTR regions are marked with small italic letters before the ATG and after the TGA codons, respectively. The ORF is shown in pink. **Fig. S3**b. Maximum likelihood phylogenetic tree of 14 COBRA-like 4 (COBL4) / irregular xylem 6 (IRX6) proteins (BlastP) with the highest degree of similarity to LlCOBL4/LlIRX6. Alignment and phylogenetic reconstructions were performed using the ETE3 v3.1.1 program as implemented in GenomeNet. The ML tree was inferred using PhyML v20160115. Branch supports are the Chi^2^-based parametric values returned by the approximate likelihood ratio test. *Lang* - *Lupinus angustifolius* (XP_019452983); *Cc* - *Cajanus cajan* (XP_020240142); *Ap* - *Abrus precatorius* (XP_027353541); *Ss* - *Spatholobus suberectus* (TKY65050); *Mp* - *Mucuna pruriens* (RDX75309); *Ad* - *Arachis duranensis* (XP_015946272); *Ai* - *Arachis ipaensis* (XP_016182359); *Vr* - *Vigna radiata* (XP_014522888); *Gs* - *Glycine soja* (XP_028215469); *Ah* - *Arachis hypogaea* (XP_025624246); *Mt* - *Medicago truncatula* (XP_013451095); *Gm* - *Glycine max* (XP_003554956); *Pa* - *Prosopis alba* (XP_028764818); *At* - *Arabidopsis thaliana* (NP_197067). **Fig. S3**c. Multiple alignment (ClustalW) of 14 COBL4/IRX6 amino acid sequences (BlastP) that are closely related to LlCOBL4/LlIRX6. Conserved domain characteristic to COBRA superfamily is marked in green background. The N-terminal predicted signal peptide is marked yellow, with cleavage site marked by black arrow. The underlined sequences with red letters show the putative cellulose-binding sites. The light blue sequences denote the Cys-rich (CCVS) motif characteristic for all COBL proteins, while CCVS amino acids are marked in dark blue background and white letters. Two conserved consensus N-glycosylation sites are indicated by a white letters on violet backgrounds. Locus corresponds to the predicted cleavage ω sites in the C terminus is indicated in middium grey background and black arrow. **Fig. S3**d. Comparison of COBL4/IRX6 proteins (BlastP) derived from different plant species using the DiAlign program (Genomatix). For each pairwise alignment, the similarity (relative to the maximum similarity) and the number of identical amino acids (in % of shorter sequence) are given. Maximum values are underlined. The similarity value of 1.000 denotes only the two most similar sequences; it does not necessarily mean that these sequences are identical. *Lang* - *Lupinus angustifolius* (XP_019452983); *Cc* - *Cajanus cajan* (XP_020240142); *Ap* - *Abrus precatorius* (XP_027353541); *Ss* - *Spatholobus suberectus* (TKY65050); *Mp* - *Mucuna pruriens* (RDX75309); *Ad* - *Arachis duranensis* (XP_015946272); *Ai* - *Arachis ipaensis* (XP_016182359); *Vr* - *Vigna radiata* (XP_014522888); *Gs* - *Glycine soja* (XP_028215469); *Ah* - *Arachis hypogaea* (XP_025624246); *Mt* - *Medicago truncatula* (XP_013451095); *Gm* - *Glycine max* (XP_003554956); *Pa* - *Prosopis alba* (XP_028764818); *At* - *Arabidopsis thaliana* (NP_197067). **Fig. S4**a. *LlGAUT12* (alternative name *LlIRX8)* cDNA (GenBank accession number MW240679, 2276 bp) identified in yellow lupine (*Lupinus luteus* L.) and its deduced amino acid sequence [533 aa (ExPASy, translate tool), m.w. = 60.673 kD and pI = 8.94 (ExPASy, ProtParam)]. The nucleotides are marked with lowercase letters, and amino acids with capital letters. The START and STOP codons (yellow background) are indicated. 5′ and 3′ UTR regions are marked with small italic letters before the ATG and after the TAG codons, respectively. The ORF is shown in pink. **Fig. S4**b. Maximum likelihood phylogenetic tree of 15 galacturonosyltransferase 12 (GAUT12) / irregular xylem 8 (IRX8) proteins (BlastP) with the highest degree of similarity to LlGAUT12/LlIRX8. Alignment and phylogenetic reconstructions were performed using the ETE3 v3.1.1 program as implemented in GenomeNet. The ML tree was inferred using PhyML v20160115. Branch supports are the Chi^2^-based parametric values returned by the approximate likelihood ratio test. *Lalb* - *Lupinus albus* (KAE9588601); *Lang* - *Lupinus angustifolius* (XP_019437125); *Gm* - *Glycine max* (XP_003543290); *Ap* - *Abrus precatorius* (XP_027341076); *Pp* - *Prunus persica* (XP_007213904); *Pm* - *Prunus mume* (XP_008244342); *Ca* - *Cicer arietinum* (XP_004487615); *Ss* - *Spatholobus suberectus* (TKY48985); *Cc* - *Cajanus cajan* (XP_020235268); *Ah* - *Arachis hypogaea* (XP_025614714); *Ad* - *Arachis duranensis* (XP_015936483); *Ai* - *Arachis ipaensis* (XP_016170753); *Md* - *Malus domestica* (XP_008349951); *Dz* - *Durio zibethinus* (XP_022716962); *At* - *Arabidopsis thaliana* (NP_200280). **Fig. S4**c. Multiple alignment (ClustalW) of 15 galacturonosyl transferase 12 (GAUT12) / irregular xylem 8 (IRX8) amino acid sequences (BlastP) that are closely related to LlGAUT12/LlIRX8. N-terminal cytoplasmic domain and the transmembrane domain (TM, yellow background) were predicted using the protein homology/analogy recognition engine V 2.0 (Phyre^2^) web portal. The specific glycosyl transferase family 8 (GT8) domain (pfam01501; black background) and the catalytic DxD motif (blue) were predicted using the CDD (NCBI). **Fig. S4**d. Comparison of GAUT12/IRX8 proteins (BlastP) derived from different plant species using the DiAlign program (Genomatix). For each pairwise alignment, the similarity (relative to the maximum similarity) and the number of identical amino acids (in % of shorter sequence) are given. Maximum values are underlined. The similarity value of 1.000 denotes only the two most similar sequences; it does not necessarily mean that these sequences are identical. *Lalb* - *Lupinus albus* (KAE9588601); *Lang* - *Lupinus angustifolius* (XP_019437125); *Gm* - *Glycine max* (XP_003543290); *Ap* - *Abrus precatorius* (XP_027341076); *Pp* - *Prunus persica* (XP_007213904); *Pm* - *Prunus mume* (XP_008244342); *Ca* - *Cicer arietinum* (XP_004487615); *Ss* - *Spatholobus suberectus* (TKY48985); *Cc* - *Cajanus cajan* (XP_020235268); *Ah* - *Arachis hypogaea* (XP_025614714); *Ad* - *Arachis duranensis* (XP_015936483); *Ai* - *Arachis ipaensis* (XP_016170753); *Md* - *Malus domestica* (XP_008349951); *Dz* - *Durio zibethinus* (XP_022716962); *At* - *Arabidopsis thaliana* (NP_200280). **Fig. S5**a. *LlPG*/*LlQRT2* cDNA (GenBank accession number MW240680, 1486 bp) identified in yellow lupine (*Lupinus luteus* L.) and its deduced amino acid sequence [428 aa (ExPASy, translate tool), m.w. = 47.623 kD and pI = 9.18 (ExPASy, ProtParam)]. The nucleotides are marked with lowercase letters, and amino acids with capital letters. The START and STOP codons (yellow background) are indicated. 5′ and 3′ UTR regions are marked with small italic letters before the ATG and after the TGA codons. The ORF is shown in pink. **Fig. S5**b. Maximum likelihood phylogenetic tree of 19 polygalacturonases (PGs) (BlastP) with the highest degree of similarity to LlPG/LlQRT2. Alignment and phylogenetic reconstructions were performed using the ETE3 v3.1.1 program as implemented in GenomeNet. ML tree was inferred using PhyML v20160115. Branch supports are the Chi^2^-based parametric values returned by the approximate likelihood ratio test. *Lang* - *Lupinus angustifolius* (XP_019425177, PG-like); *Lalb* - *Lupinus albus* (KAE9590458, putative PG); *Gm* - *Glycine max* (XP_003545985, PG); *Gs* - *Glycine soja* (KHM99207, PG); *Ca* - *Cicer arietinum* (XP_004499889, PG QRT2); *Ap* - *Abrus precatorius* (XP_027343054, PG); *Va* - *Vigna angularis* (XP_017424613, PG); *Ad* - *Arachis duranensis* (XP_015952899, PG QRT2-like); *Ah* - *Arachis hypogaea* (XP_025642914, PG QRT2); *Ai* - *Arachis ipaensis* (XP_020965356, PG); *Mt* - *Medicago truncatula* (XP_003595746, PG); *Cc* - *Cajanus cajan* (XP_020214988, PG); *Cm* - *Cucurbita maxima* (XP_022982564, PG); *Rc* - *Ricinus communis* (XP_002517823, PG QRT2); *Gh* - *Gossypium hirsutum* (XP_016741024, PG QRT2-like); *At* – *Arabidopsis thaliana* (O23147, ARABIDOPSIS DEHISCENCE ZONE POLYGALACTURONASE 1, ADPG1); *A. thaliana* (Q8RY29, ADPG2); *A. thaliana* (Q9SFB7, QUARTET2, QRT2); *Sl* - *Solanum lycopersicum* (XP_004252072 PG QRT2). **Fig. S5**c. Multiple sequence alignment (ClustalW) of different polygalacturonases (PGs, BlastP) showing the highest similarity to LlPG/LlQRT2. Four typical conserved domains of PGs, referred to as domains I, II, III and IV (RIKT) were indicatated in yellow, green, blue and red, respectively. A tyrosine (Y) is marked in pink. 12 cysteine (C) residues are marked in red letters. Three aspartic acids in NTD and DD structures (domains I and II, respectively) are marked with gray arrows. The histidine residue (H) in domain III is marked with purple arrow. **Fig. S5**d. Comparison of PGs derived from different plant species using the DiAlign program (Genomatix). For each pairwise alignment, the similarity (relative to the maximum similarity) and the number of identical amino acids (in % of shorter sequence) are given. Maximum values are underlined. The similarity value of 1.000 denotes only the two most similar sequences; it does not necessarily mean that these sequences are identical. *Lang* - *Lupinus angustifolius* (XP_019425177); *Lalb* - *Lupinus albus* (KAE9590458); *Gm* - *Glycine max* (XP_003545985); *Gs* - *Glycine soja* (KHM99207); *Ca* - *Cicer arietinum* (XP_004499889); *Ap* - *Abrus precatorius* (XP_027343054); *Va* - *Vigna angularis* (XP_017424613); *Ad* - *Arachis duranensis* (XP_015952899); *Ah* - *Arachis hypogaea* (XP_025642914); *Ai* - *Arachis ipaensis* (XP_020965356); *Mt* - *Medicago truncatula* (XP_003595746); *Cc* - *Cajanus cajan* (XP_020214988); *Cm* - *Cucurbita maxima* (XP_022982564); *Rc* - *Ricinus communis* (XP_002517823); *Gh* - *Gossypium hirsutum* (XP_016741024); *At* - *Arabidopsis thaliana* (O23147, ADPG1); *A. thaliana* (Q8RY29, ADPG2); *A. thaliana* (Q9SFB7, QRT2); *Sl* - *Solanum lycopersicum* (XP_004252072). **Fig. S6**a. *LlPCS1L* cDNA (GenBank accession number MW240681, 1773 bp) identified in yellow lupine (*Lupinus luteus* L.) and its deduced amino acid sequence [480 aa (ExPASy, translate tool), m.w. = 53.044 kD and pI = 5.42 (ExPASy, ProtParam)]. The nucleotides are marked with lowercase letters, and amino acids with capital letters. The START and STOP codons (yellow background) are indicated. 5′ and 3′ UTR regions are marked with small italic letters before the ATG and after the TGA codons, resceptively. The ORF is shown in pink. **Fig. S6**b. Maximum likelihood phylogenetic tree of 15 PCS1 proteins (BlastP) with the highest degree of similarity to LlPCS1. Alignment and phylogenetic reconstructions were performed using the ETE3 v3.1.1 program as implemented in GenomeNet. The ML tree was inferred using PhyML v20160115. Branch supports are the Chi^2^-based parametric values returned by the approximate likelihood ratio test. *Lang* - *Lupinus angustifolius* (XP_019412745); *Vr* - *Vigna radiata* (XP_014497350); *Gm* - *Glycine max* (XP_003530215); *Mt* - *Medicago truncatula* (XP_013448893); Ap - *Abrus precatorius* (XP_027349738); Cc - *Cajanus cajan* (XP_020206919); *Vu* - *Vigna unguiculata* (XP_027927273); *Ca* - *Cicer arietinum* (XP_004514393); *Tp* - *Trifolium pratense* (PNX74271); *Ql* - *Quercus lobata* (XP_030925127); *Pa* - *Prosopis alba* (XP_028786489); *St* - *Senna tora* (KAF7825648); *Qs* - *Quercus suber* (XP_023913890); *Tw* - *Tripterygium wilfordii* (KAF5750924); *At* - *Arabidopsis thaliana* (OAO90380). **Fig. S6**c. Multiple alignment (ClustalW) of 15 PROMOTION OF CELL SURVIVAL1 (PCS1) amino acid sequences (BlastP) that are closely related to LlPCS1. Two motif in both N-terminal (DTGS) and C-terminal (DS/LGT) ends characteristic for pepsin like aspartic proteases are marked in yellow and light grey, respectivelly. Additionally, two catalytic residues (D) (white letters on red background) have been indicated. Active site flap ATLS and SSSS are marked green and light blue, respectively. Pepsin A like plant domain is shaded dark blue with white letters. TAXi_N domain (Xylanase inhibitor N-terminal, sea color) and TAXi_C domain (violet) are additionally underlined. *Lang* - *Lupinus angustifolius* (XP_019412745); *Vr* - *Vigna radiata* (XP_014497350); *Gm* - *Glycine max* (XP_003530215); *Mt* - *Medicago truncatula* (XP_013448893); Ap - *Abrus precatorius* (XP_027349738); Cc - *Cajanus cajan* (XP_020206919); *Vu* - *Vigna unguiculata* (XP_027927273); *Ca* - *Cicer arietinum* (XP_004514393); *Tp* - *Trifolium pratense* (PNX74271); *Ql* - *Quercus lobata* (XP_030925127); *Pa* - *Prosopis alba* (XP_028786489); *St* - *Senna tora* (KAF7825648); *Qs* - *Quercus suber* (XP_023913890); *Tw* - *Tripterygium wilfordii* (KAF5750924); *At* - *Arabidopsis thaliana* (OAO90380). **Fig. S6**d. Comparison of PCS1 proteins derived from different plant species using the DiAlign program (Genomatix). For each pairwise alignment, the similarity (relative to the maximum similarity) and the number of identical amino acids (in % of shorter sequence) are given. Maximum values are underlined. The similarity value of 1.000 marks only the two most similar sequences; it does not necessarily mean that these sequences are identical. *Lang* - *Lupinus angustifolius* (XP_019412745); *Vr* - *Vigna radiata* (XP_014497350); *Gm* - *Glycine max* (XP_003530215); *Mt* - *Medicago truncatula* (XP_013448893); Ap - *Abrus precatorius* (XP_027349738); Cc - *Cajanus cajan* (XP_020206919); *Vu* - *Vigna unguiculata* (XP_027927273); *Ca* - *Cicer arietinum* (XP_004514393); *Tp* - *Trifolium pratense* (PNX74271); *Ql* - *Quercus lobata* (XP_030925127); *Pa* - *Prosopis alba* (XP_028786489); *St* - *Senna tora* (KAF7825648); *Qs* - *Quercus suber* (XP_023913890); *Tw* - *Tripterygium wilfordii* (KAF5750924); *At* - *Arabidopsis thaliana* (OAO90380). **Fig. S7**a. *LlGA3ox* cDNA (GenBank accession number MW240682, 1311 bp) identified in yellow lupine (*Lupinus luteus* L.) and its deduced amino acid sequence [320 aa (ExPASy, translate tool), m.w. = 36.576 kD and pI = 5.67 (ExPASy, ProtParam)]. The nucleotides are marked with lowercase letters, and amino acids with capital letters. The START and STOP codons (yellow background) are indicated. 5′ and 3′ UTR regions are marked with small italic letters before the ATG and after the TGA codons, resceptively. The ORF is shown in pink. **Fig. S7**b. Maximum likelihood phylogenetic tree of 10 GA3ox proteins (BlastP) with the highest degree of similarity to LlGA3ox. Alignment and phylogenetic reconstructions were performed using the ETE3 v3.1.1 program as implemented in GenomeNet. The ML tree was inferred using PhyML v20160115. Branch supports are the Chi^2^-based parametric values returned by the approximate likelihood ratio test. *Lang* - *Lupinus angustifolius* (XP_019459798); *Lalb* - *Lupinus albus* (KAE9605408); *Va* - *Vigna angularis* (XP_017408358); *Ss* - *Spatholobus suberectus* (TKY63915); *Gs* - *Glycine soja* (KHN29357); *Vv* - *Vitis vinifera* (XP_019072492); *St* - *Solanum tuberosum* (XP_006339568); *Ns* - *Nicotiana sylvestris* (XP_009779750); *Na* - *Nicotiana attenuata* (XP_019262262); *At* -*Arabidopsis thaliana* (NP_178149). **Fig. S7**c. Multiple alignment (ClustalW) of 10 GIBBERELLIN 3-OXIDASE (GA3ox) amino acid sequences (BlastP) that are closely related to LlGA3ox. Gibberellin 3-β-dioxygenase domain (black background) and domain characteristic for 2-oxoglutarate (2OG) and Fe (II)-dependent oxygenase (Oxy) superfamily (blue background) are indicated. The putative His-x-Asp-(x)n-His (HxD … H) and Arg-x-Ser (RxS) motif locations are highlighted in red and green, respectively. Domains and specific, highly conserved motifs were delineated from the CDD (NCBI) and [53]. *Lang* - *Lupinus angustifolius* (XP_019459798); *Lalb* - *Lupinus albus* (KAE9605408); *Va* - *Vigna angularis* (XP_017408358); *Ss* - *Spatholobus suberectus* (TKY63915); *Gs* - *Glycine soja* (KHN29357); *Vv* - *Vitis vinifera* (XP_019072492); *St* - *Solanum tuberosum* (XP_006339568); *Ns* - *Nicotiana sylvestris* (XP_009779750); *Na* - *Nicotiana attenuata* (XP_019262262); *At* -*Arabidopsis thaliana* (NP_178149). **Fig. S7**d. Comparison of GIBBERELLIN 3-OXIDASES (GA3oxs) derived from different plant species using the DiAlign program (Genomatix). For each pairwise alignment, the similarity (relative to the maximum similarity) and the number of identical amino acids (in % of shorter sequence) are given. Maximum values are underlined. The similarity value of 1.000 marks only the two most similar sequences; it does not necessarily mean that these sequences are identical. *Lang* - *Lupinus angustifolius* (XP_019459798); *Lalb* - *Lupinus albus* (KAE9605408); *Va* - *Vigna angularis* (XP_017408358); *Ss* - *Spatholobus suberectus* (TKY63915); *Gs* - *Glycine soja* (KHN29357); *Vv* - *Vitis vinifera* (XP_019072492); *St* - *Solanum tuberosum* (XP_006339568); *Ns* - *Nicotiana sylvestris* (XP_009779750); *Na* - *Nicotiana attenuata* (XP_019262262); *At* -*Arabidopsis thaliana* (NP_178149). **Fig. S8**a. *LlGA2ox1* cDNA (GenBank accession number MG181996, 1458 bp) identified in yellow lupine (*Lupinus luteus* L.) and its deduced amino acid sequence [330 aa (ExPASy, translate tool), m.w. = 37.005 kD and pI = 8.14 (ExPASy, ProtParam)]. The nucleotides are marked with lowercase letters, and amino acids with capital letters. The START and STOP codons (yellow background) are indicated. 5′ and 3′ UTR regions are marked with small italic letters before the ATG and after the TGA codons, resceptively. The ORF is shown in pink. **Fig. S8**b. Maximum likelihood phylogenetic tree of 15 GIBBERELLIN 2-OXIDASES (GA2oxs, BlastP) with the highest degree of similarity to LlGA2ox1. Alignment and phylogenetic reconstructions were performed using the ETE3 v3.1.1 as implemented in GenomeNet. The ML tree was inferred using PhyML v20160115. Branch supports are the Chi^2^-based parametric values returned by the approximate likelihood ratio test. *Lang* - *Lupinus angustifolius* (XP_019424643); *Lalb* - *Lupinus albus* (KAE9590556); *Mp* - *Mucuna pruriens* (RDX87676); *Ss* - *Spatholobus suberectus* (TKY49301); *Ap* - *Abrus precatorius* (XP_027342450); *Va* - *Vigna angularis* (NP_001316752); *Cc* - *Cajanus cajan* (XP_020204195); *Vu* - *Vigna unguiculata* (XP_027926310); *Gm* - *Glycine max* (XP_003543155); *Ps* - *Pisum sativum* (AAD45425); *Gs* - *Glycine soja* (XP_028201454); *Ca* - *Cicer arietinum* (XP_004488652); *Jr* - *Juglans regia* (XP_018824979); *Nt* - *Nicotiana tabacum* (XP_016500243); *At* - *Arabidopsis thaliana* (Q8LEA2). **Fig. S8**c. Multiple alignment (ClustalW) of 15 GIBBERELLIN 2-OXIDASE (GA2ox) amino acid sequences (BlastP) that are closely related to LlGA2ox1. Gibberellin 2-β-dioxygenase domain (black background) and domain characteristic for 2-oxoglutarate (2OG)-Fe (II)-dependent oxygenase superfamily (blue background) are indicated. Both domains designated using the CDD (NCBI). Additionally, amino acid residues presumed to bind Fe^2+^ at the active site of protein are indicated with red (HxD … H) and green (RxS) letters. **Fig. S8**d. Comparison of GIBBERELLIN 2-OXIDASES (GA2oxs) derived from different plant species using the DiAlign program (Genomatix). For each pairwise alignment, the similarity (relative to the maximum similarity) and the number of identical amino acids (in % of shorter sequence) are given. Maximum values are underlined. The similarity value of 1.000 marks only the two most similar sequences; it does not necessarily mean that these sequences are identical. *Lang* - *Lupinus angustifolius* (XP_019424643); *Lalb* - *Lupinus albus* (KAE9590556); *Mp* - *Mucuna pruriens* (RDX87676); *Ss* - *Spatholobus suberectus* (TKY49301); *Ap* - *Abrus precatorius* (XP_027342450); *Va* - *Vigna angularis* (NP_001316752); *Cc* - *Cajanus cajan* (XP_020204195); *Vu* - *Vigna unguiculata* (XP_027926310); *Gm* - *Glycine max* (XP_003543155); *Ps* - *Pisum sativum* (AAD45425); *Gs* - *Glycine soja* (XP_028201454); *Ca* - *Cicer arietinum* (XP_004488652); *Jr* - *Juglans regia* (XP_018824979); *Nt* - *Nicotiana tabacum* (XP_016500243); *At* - *Arabidopsis thaliana* (Q8LEA2). **Fig. S9**a. *LlGAMYB* cDNA (GenBank accession number MW240675, 2038 bp) identified in yellow lupine (*Lupinus luteus* L.) and its deduced amino acid sequence [533 aa (ExPASy, translate tool), m.w. = 59.048 kD and pI = 5.25 (ExPASy, ProtParam)]. The nucleotides are marked with lowercase letters, and amino acids with capital letters. The START and STOP codons (yellow background) are indicated. 5′ and 3′ UTR regions are marked with small italic letters before the ATG and after the TGA codons, resceptively. The ORF is shown in pink. 21 nucleotide sequence in blue background corresponds to target site for Ll-miR159. **Fig. S9**b. Maximum likelihood phylogenetic tree of 16 GAMYB proteins (BlastP) with the highest degree of similarity to LlGAMYB. Alignment and phylogenetic reconstructions were performed using the ETE3 v3.1.1 as implemented in GenomeNet. The ML tree was inferred using PhyML v20160115. Branch supports are the Chi^2^-based parametric values returned by the approximate likelihood ratio test. *Lang* - *Lupinus angustifolius* (XP_019449331); *Lalb* - *Lupinus albus* (KAE9617167); *Ss* - *Spatholobus suberectus* (TKY68413); *Cc* - *Cajanus cajan* (XP_020219565); Ap - *Abrus precatorius* (XP_027354365); *Mp* - *Mucuna pruriens* (RDX95167); *Gm* - *Glycine max* (AHB19229); *Gs* - *Glycine soja* (XP_028187659); *Ah* - *Arachis hypogaea* (XP_025615172); *Ai* - *Arachis ipaensis* (XP_016171706); *Ad* - *Arachis duranensis* (XP_015937001); *At* - *Arabidopsis thaliana* (AAS10086, AtMYB33), (AAS10055, AtMYB65), (NP_194423, AtMYB97), (NP_001077993, AtMYB101), (NP_568819, AtMYB120). **Fig. S9**c. Multiple alignment (ClustalW) of 16 MYB amino acid sequences (BlastP) that are closely related to LlGAMYB. The R2R3 domain near the 5′ terminus (black) and Box 1 (yellow), Box 2 (green), Box 3 (blue) domains distributed throughout the protein were designated. REB1 domain characteristic for Myb superfamily proteins (underlined), Myb_DNA-binding domain (pink) and SANT (SWI3, ADA2, N-CoR and TFIIIB” DNA-binding) domain (gray) have been designated using the CDD (NCBI). *Lang* - *Lupinus angustifolius* (XP_019449331); *Lalb* - *Lupinus albus* (KAE9617167); *Ss* - *Spatholobus suberectus* (TKY68413); *Cc* - *Cajanus cajan* (XP_020219565); Ap - *Abrus precatorius* (XP_027354365); *Mp* - *Mucuna pruriens* (RDX95167); *Gm* - *Glycine max* (AHB19229); *Gs* - *Glycine soja* (XP_028187659); *Ah* - *Arachis hypogaea* (XP_025615172); *Ai* - *Arachis ipaensis* (XP_016171706); *Ad* - *Arachis duranensis* (XP_015937001); *At* - *Arabidopsis thaliana* (AAS10086, AtMYB33), (AAS10055, AtMYB65), (NP_194423, AtMYB97), (NP_001077993, AtMYB101), (NP_568819, AtMYB120). **Fig. S9**d. Comparison of GAMYB proteins derived from different plant species using the DiAlign program (Genomatix). For each pairwise alignment, the similarity (relative to the maximum similarity) and the number of identical amino acids (in % of shorter sequence) are given. Maximum values are underlined. The similarity value of 1.000 marks only the two most similar sequences; it does not necessarily mean that these sequences are identical. *Lang* - *Lupinus angustifolius* (XP_019449331); *Lalb* - *Lupinus albus* (KAE9617167); *Ss* - *Spatholobus suberectus* (TKY68413); *Cc* - *Cajanus cajan* (XP_020219565); Ap - *Abrus precatorius* (XP_027354365); *Mp* - *Mucuna pruriens* (RDX95167); *Gm* - *Glycine max* (AHB19229); *Gs* - *Glycine soja* (XP_028187659); *Ah* - *Arachis hypogaea* (XP_025615172); *Ai* - *Arachis ipaensis* (XP_016171706); *Ad* - *Arachis duranensis* (XP_015937001); *At* - *Arabidopsis thaliana* (AAS10086, AtMYB33), (AAS10055, AtMYB65), (NP_194423, AtMYB97), (NP_001077993, AtMYB101), (NP_568819, AtMYB120). **Fig. S10**. The cDNA sequence of *Ll-MIR159* identified in yellow lupine (GenBank accession number MW240683). The 21-nucleotide fragment that constitutes the mature miR159 is marked in yellow. **Fig. S11**. The domain structures of LlCAD (cinnamyl alcohol dehydrogenase), LlCesA8/LlIRX1 (cellulose synthase A catalytic subunit 8/IRREGULAR XYLEM1), LlCOBL4/IRX6 (COBRA-like4), LlGAUT12/LlIRX8 (galacturonosyltransferase 12), LlPG/LlQRT2 (polygalacturonase/ QUARTET2) and LlPCS1 (PROMOTION OF CELL SURVIVAL1) identified in yellow lupine (*Lupinus luteus* L.). (A-F) The predicted tertiary 3D models constructed by the ROBETTA protein modelling server visualized by the ChimeraX program. (A’-F′) Conserved domains, motifs and specific amino acids identified via different tools described in the Methods section. The colours used in the 3D protein models correspond to the colours presented in the protein diagrams. The specific functions of domains/motifs/amino acids are described in detail in Table 1 (main manuscript). **Fig. S12**. The domain structures of LlGA3ox (gibberellin 3-oxidase), LlGA2ox1 (gibberellin 2-oxidase1) and LlGAMYB identified in yellow lupine (*Lupinus luteus* L.). (A-C) The predicted tertiary 3D models constructed by the ROBETTA protein modelling server visualized by the ChimeraX program. (A’-C′) Conserved domains, motifs and specific amino acids identified via different tools described in the Method section. The colours used in the 3D protein models correspond to the colours presented in the protein diagrams. The specific functions of domains/motifs/amino acids are described in detail in Table 1 (main manuscript). **Fig. S13**. (A) Image of the electrophoretic separation of the PCR product carried out with degenerate primers to identify a cDNA fragment of *LlGAMYB* in yellow lupine on a 1.2% agarose gel in 0,5 × TBE buffer at 5 V/cm in the presence of the GeneRuler 100 bp DNA ladder marker (*M*, Fermentas); (B) Simplified diagram of the pSC-A-amp/kan vector (Agilent Technologies); (C) Representative Petri dish containing S-Gal/LB agar blend with one shot Mach1-T1 *E. coli*. White bacterial colonies took the recombinant form of the pCRII-TOPO vector, while dark blue bacterial colonies took the vector without the insert. (D) Image of electrophoretic separation of plasmid DNA digested with the restriction enzyme *Eco*RI (Fermentas) to confirm the presence of the insert - the cDNA fragment of *LlGAMYB*. Separation was performed on a 1.2% agarose gel in 0.5 × TBE buffer at 5 V/cm and in the presence of a GeneRuler 100 bp DNA ladder marker (*M*). The arrow indicates the sample that was sent for sequencing (Genomed, Warsaw, Poland). **Fig. S14**. (A, B) Electrophoretic separation images of the PCR products (A, the results obtained with the first pair of primers, specific + degenerate) and 3’RACE-PCR (B, the results obtained with the second pair of primers) for *LlGAMyb* in 1.2% agarose gels in 0.5 × TBE buffer at 5 V/cm in the presence of GeneRuler 100 bp DNA Ladder marker (*M*, Fermentas); (C, D) Electrophoretic separation images of plasmid DNA containing inserts of various sizes that were digested with the restriction enzyme *Eco*RI (Fermentas) on 1.2% agarose gels in 0.5 × TBE buffer at 5 V/cm and in the presence of GeneRuler 100 bp DNA Ladder marker (*M*); Transformation results obtained with the reaction products carried out using the first pair of primers (C) and the second pair of primers (D); arrows indicate the individual samples that were sent for sequencing (Genomed, Warsaw, Poland). **Fig. S15**. The negative control required for validation of the immunohistochemical reactions was carried out by omitting incubation with the primary antibody, and showed no labelling. The autofluorescence signal of the cell walls and pollen grains is visible. DAPI was used to stain cell nuclei. Scale bar = 50 μm. Tab. S1. (A) Sequences of degenerate primers designed based on the cDNA sequences of *GAMYB* genes derived from different plant species (BlastP); (B) Sequences of specific, degenerate and universal primers used in 3′ RACE (rapid amplification of cDNA ends)-PCR; GSOP – gene specific outer primer, OP – outer primer, GSIP – gene specific inner primer, IP – inner primer; (C) Sequences of specific primers, their melting temperature (Tm) and UPL (UNIVERSAL PROBE LIBRARY) probe sequences and numbers used in qPCR for all studied genes and *LlACT*. The length of the amplified product in PCR and the annealing temperature of the primers are also given. (PPTX 19524 kb)

## Data Availability

The data supporting the conclusions of this article are included within the article and its additional files. The cDNA sequences of all identified genes of yellow lupine are available at GenBank (NCBI) under accession numbers: MW240675, MW240676, MW240677, MW240678, MW240679, MW240680, MW240681, MW240682, MW240683 and MG181996. The nucleotide and protein sequences were downloaded from GenBank and Protein database (NCBI), respectively. The RNA-Seq data are available at the NCBI in the Sequence Read Archive (SRA) database under accession number PRJNA285604 (BioProject) and experiment accession number SRX1069734.

## Abbreviations

ACT: actin
ADPG1/2: Arabidopsis dehiscence zone polygalacturonase 1/2
AHP4: Arabidopsis histidine-containing phosphotransfer 4
C: Connective
CAD: Cinnamyl alcohol dehydrogenase
CesA8: Cellulose synthase A catalytic subunit 8 [UDP-forming]
CKs: Cytokinins
COBL4: COBRA-like 4
E: Epidermis
En: Endothecium
GA_3_: Gibberellic acid
GA3ox: Gibberellin 3-oxidase
GA2ox: Gibberellin 2-oxidases
GAs: Gibberellins
GAUT12: Galacturonosyltransferase 12
GPI: Glycosylphosphatidylinositol
GT8: Glycosyl transferase family 8
IRX1/6/8: Irregular xylem 1/6/8
LAD: Late anther development
PAC: Paclobutrazol
PCD: Programmed cell death
PCS1: Promotion of cell survival 1
P: Pollen grain
PGs: Polygalacturonases
QRT2: Quartet 2
Se: Septum
St: Stomium
StR: Stomium region
T: Tapetum
VB: Vascular bundle
