# Supplementary material for: Anther dehiscence is regulated by gibberellic acid in yellow lupine (Lupinus luteus L.)
Source: BMC Plant Biol. 2021 Jul 2;21:314. doi: 10.1186/s12870-021-03085-4 (PMC8252261; doi:10.1186/s12870-021-03085-4)
Supplement: Supplementary file 1 — Additional file 1: Fig. S1a. LlCAD cDNA (GenBank accession number MW240676, 2250 bp) identified in yellow lupine (Lupinus luteus L.) and its deduced amino acid sequence [357 aa (ExPASy, translate tool), m.w. = 38.936 kD and pI = 6.01 (ExPASy, ProtParam)]. The nucleotides are marked with lowercase letters, and amino acids are marked with capital letters. The START and STOP codons (yellow background) are indicated. 5′ and 3′ UTR regions are marked with small italic letters before the ATG and after the TGA codons, respectively. The ORF is shown in pink. Fig. S1b. Maximum likelihood phylogenetic tree of 15 cinnamyl alcohol dehydrogenase (CAD) proteins (BlastP) with the highest degree of similarity to LlCAD. Alignment and phylogenetic reconstructions were performed using the Environment for Tree Exploration3 (ETE3) v3.1.1 program as implemented in GenomeNet. The ML tree was inferred using PhyML v20160115. Branch supports are the Chi2-based parametric values returned by the approximate likelihood ratio test. Fig. S1c. Multiple alignment (ClustalW) of 15 cinnamyl alcohol dehydrogenase (CAD) amino acid sequences (BlastP) that are closely related to LlCAD. The alcohol dehydrogenease GroES-like domain is shaded medium grey, and the zinc-binding dehydrogenase domain is shaded dark grey. The Zn-1 and Zn-2 binding motifs are shaded with violet. Three green amino acids, C, H, and C, are marked with green dots. Yellow letters define four cysteine (C) residues. Conserved glycine (G) residues (GxGGxG) are indicated with red letters. They represent NADPH cosubstrate-binding motif, which is highlighted in green. The conserved residues (S, Q, L, M, W, V, P, L, F, I) are highlighted in blue. The serine (S) 212 is labelled with a red background. Most of the alignment information was identified according to the results described by [38–40]. Lang - Lupinus angustifolius (XP_019452456); Lalb - Lupinus albus (KAE9589372); Ap - Abrus precatorius (XP_027363622); Vu - Vigna unguiculata (XP_0279 [file 12870_2021_3085_MOESM1_ESM.pptx]

## Slide 1
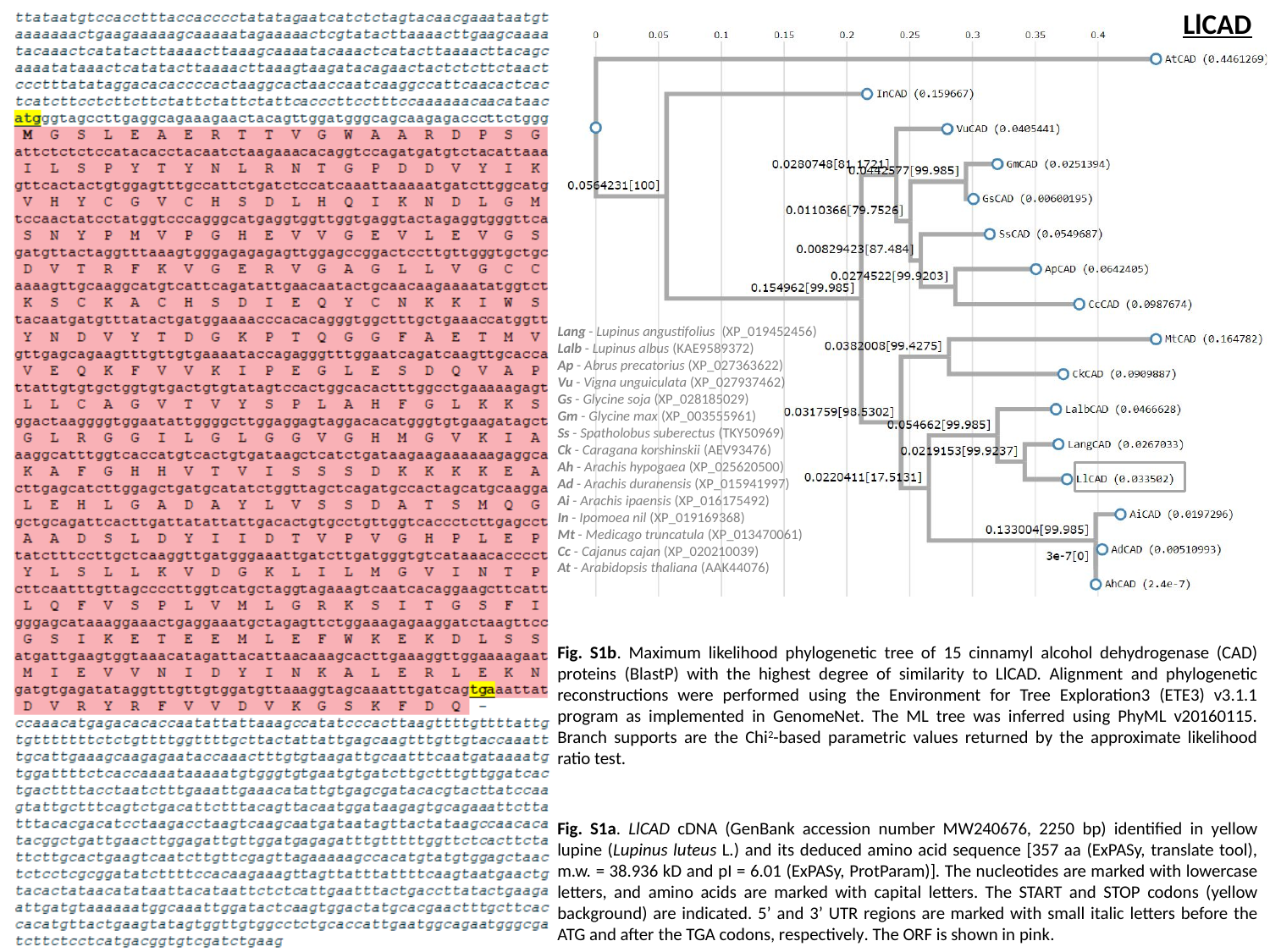

LlCAD
Lang - Lupinus angustifolius (XP_019452456)
Lalb - Lupinus albus (KAE9589372)
Ap - Abrus precatorius (XP_027363622)
Vu - Vigna unguiculata (XP_027937462)
Gs - Glycine soja (XP_028185029)
Gm - Glycine max (XP_003555961)
Ss - Spatholobus suberectus (TKY50969)
Ck - Caragana korshinskii (AEV93476)
Ah - Arachis hypogaea (XP_025620500)
Ad - Arachis duranensis (XP_015941997)
Ai - Arachis ipaensis (XP_016175492)
In - Ipomoea nil (XP_019169368)
Mt - Medicago truncatula (XP_013470061)
Cc - Cajanus cajan (XP_020210039)
At - Arabidopsis thaliana (AAK44076)
Fig. S1b. Maximum likelihood phylogenetic tree of 15 cinnamyl alcohol dehydrogenase (CAD) proteins (BlastP) with the highest degree of similarity to LlCAD. Alignment and phylogenetic reconstructions were performed using the Environment for Tree Exploration3 (ETE3) v3.1.1 program as implemented in GenomeNet. The ML tree was inferred using PhyML v20160115. Branch supports are the Chi2-based parametric values returned by the approximate likelihood ratio test.
Fig. S1a. LlCAD cDNA (GenBank accession number MW240676, 2250 bp) identified in yellow lupine (Lupinus luteus L.) and its deduced amino acid sequence [357 aa (ExPASy, translate tool), m.w. = 38.936 kD and pI = 6.01 (ExPASy, ProtParam)]. The nucleotides are marked with lowercase letters, and amino acids are marked with capital letters. The START and STOP codons (yellow background) are indicated. 5’ and 3’ UTR regions are marked with small italic letters before the ATG and after the TGA codons, respectively. The ORF is shown in pink.

## Slide 2
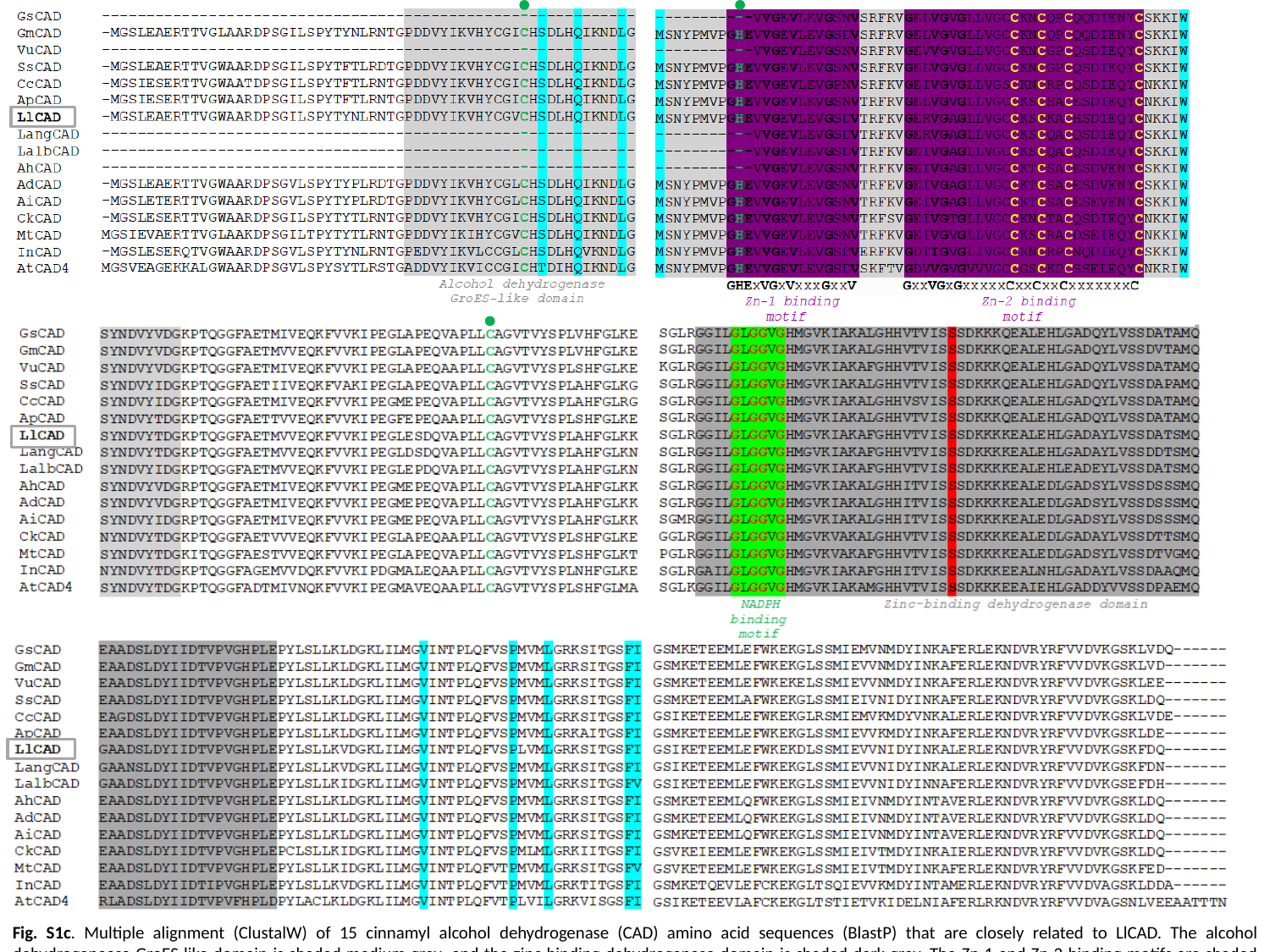

Fig. S1c. Multiple alignment (ClustalW) of 15 cinnamyl alcohol dehydrogenase (CAD) amino acid sequences (BlastP) that are closely related to LlCAD. The alcohol dehydrogenease GroES-like domain is shaded medium grey, and the zinc-binding dehydrogenase domain is shaded dark grey. The Zn-1 and Zn-2 binding motifs are shaded with violet. Three green amino acids, C, H, and C, are marked with green dots. Yellow letters define four cysteine (C) residues. Conserved glycine (G) residues (GxGGxG) are indicated with red letters. They represent NADPH cosubstrate-binding motif, which is highlighted in green. The conserved residues (S, Q, L, M, W, V, P, L, F, I) are highlighted in blue. The serine (S) 212 is labelled with a red background. Most of the alignment information was identified according to the results described by [38, 39, 40]. Lang - Lupinus angustifolius (XP_019452456); Lalb - Lupinus albus (KAE9589372); Ap - Abrus precatorius (XP_027363622); Vu - Vigna unguiculata (XP_027937462); Gs - Glycine soja (XP_028185029); Gm - Glycine max (XP_003555961); Ss - Spatholobus suberectus (TKY50969); Ck - Caragana korshinskii (AEV93476); Ah - Arachis hypogaea (XP_025620500); Ad - Arachis duranensis (XP_015941997); Ai - Arachis ipaensis (XP_016175492); In - Ipomoea nil (XP_019169368); Mt - Medicago truncatula (XP_013470061); Cc - Cajanus cajan (XP_020210039); At - Arabidopsis thaliana (AAK44076).

## Slide 3
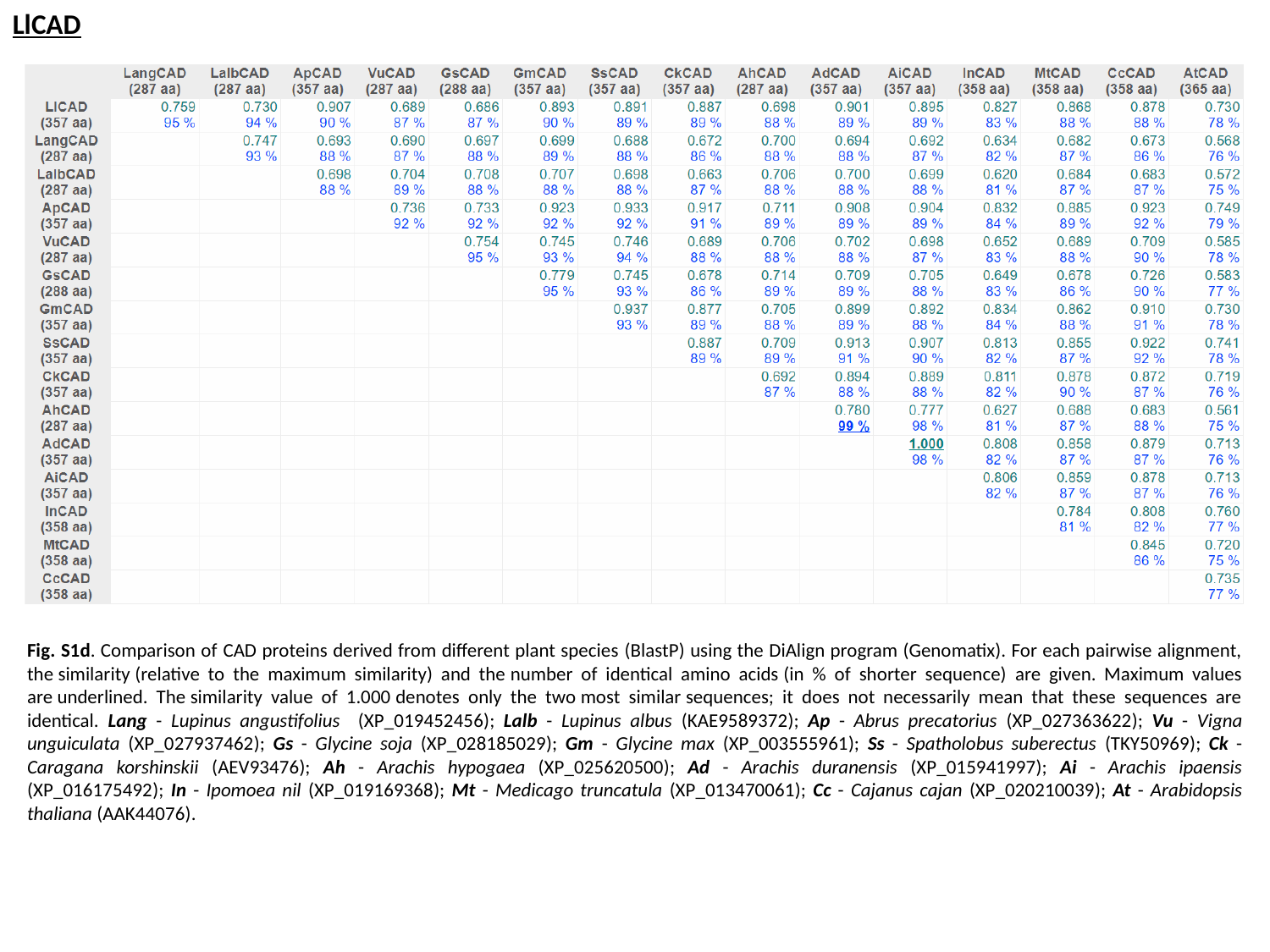

LlCAD
Fig. S1d. Comparison of CAD proteins derived from different plant species (BlastP) using the DiAlign program (Genomatix). For each pairwise alignment, the similarity (relative to the maximum similarity) and the number of identical amino acids (in % of shorter sequence) are given. Maximum values are underlined. The similarity value of 1.000 denotes only the two most similar sequences; it does not necessarily mean that these sequences are identical. Lang - Lupinus angustifolius (XP_019452456); Lalb - Lupinus albus (KAE9589372); Ap - Abrus precatorius (XP_027363622); Vu - Vigna unguiculata (XP_027937462); Gs - Glycine soja (XP_028185029); Gm - Glycine max (XP_003555961); Ss - Spatholobus suberectus (TKY50969); Ck - Caragana korshinskii (AEV93476); Ah - Arachis hypogaea (XP_025620500); Ad - Arachis duranensis (XP_015941997); Ai - Arachis ipaensis (XP_016175492); In - Ipomoea nil (XP_019169368); Mt - Medicago truncatula (XP_013470061); Cc - Cajanus cajan (XP_020210039); At - Arabidopsis thaliana (AAK44076).

## Slide 4
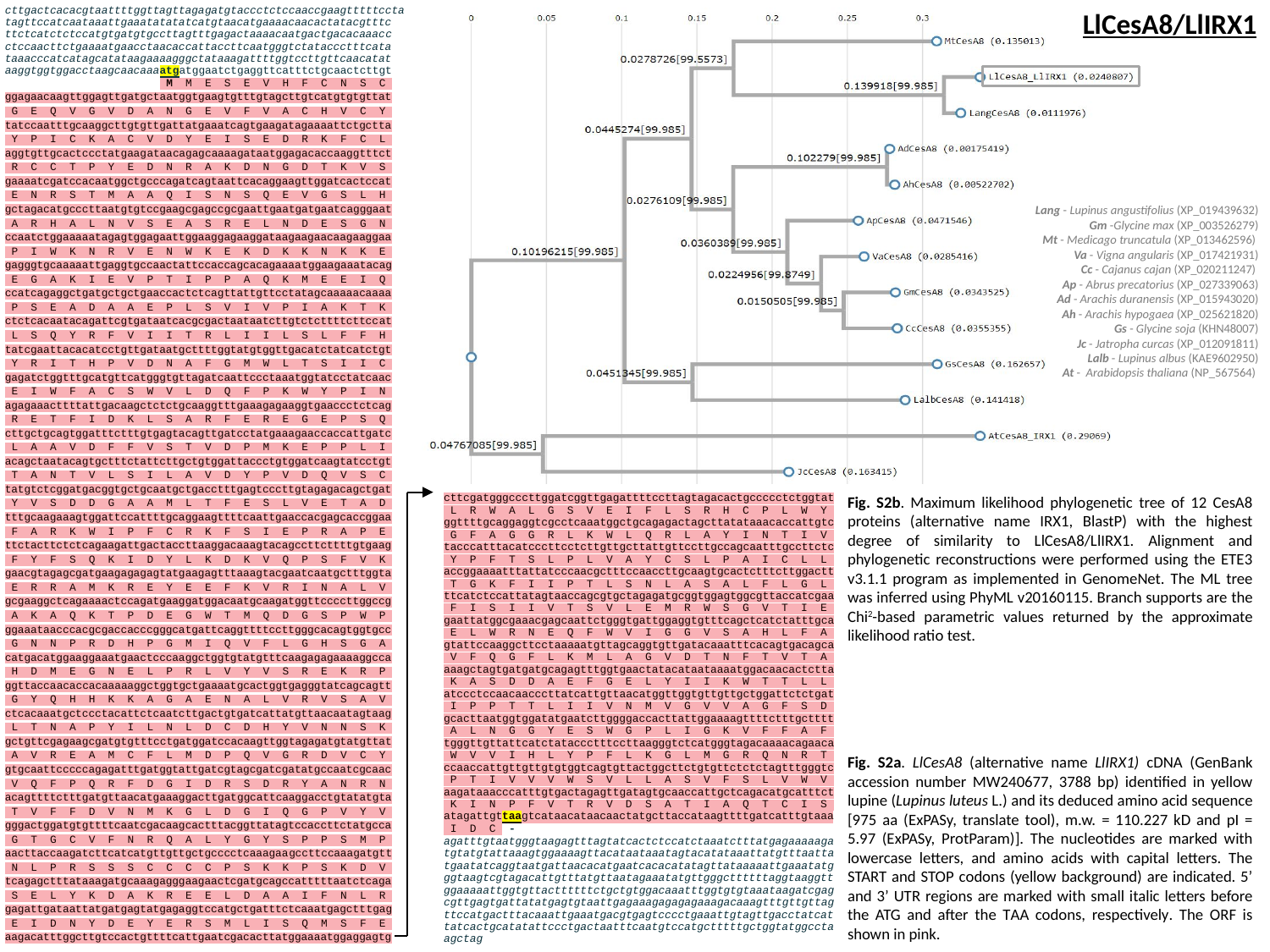

LlCesA8/LlIRX1
Lang - Lupinus angustifolius (XP_019439632)
Gm -Glycine max (XP_003526279)
Mt - Medicago truncatula (XP_013462596)
Va - Vigna angularis (XP_017421931)
Cc - Cajanus cajan (XP_020211247)
Ap - Abrus precatorius (XP_027339063)
Ad - Arachis duranensis (XP_015943020)
Ah - Arachis hypogaea (XP_025621820)
Gs - Glycine soja (KHN48007)
 Jc - Jatropha curcas (XP_012091811)
Lalb - Lupinus albus (KAE9602950)
At - Arabidopsis thaliana (NP_567564)
Fig. S2b. Maximum likelihood phylogenetic tree of 12 CesA8 proteins (alternative name IRX1, BlastP) with the highest degree of similarity to LlCesA8/LlIRX1. Alignment and phylogenetic reconstructions were performed using the ETE3 v3.1.1 program as implemented in GenomeNet. The ML tree was inferred using PhyML v20160115. Branch supports are the Chi2-based parametric values returned by the approximate likelihood ratio test.
Fig. S2a. LlCesA8 (alternative name LlIRX1) cDNA (GenBank accession number MW240677, 3788 bp) identified in yellow lupine (Lupinus luteus L.) and its deduced amino acid sequence [975 aa (ExPASy, translate tool), m.w. = 110.227 kD and pI = 5.97 (ExPASy, ProtParam)]. The nucleotides are marked with lowercase letters, and amino acids with capital letters. The START and STOP codons (yellow background) are indicated. 5’ and 3’ UTR regions are marked with small italic letters before the ATG and after the TAA codons, respectively. The ORF is shown in pink.

## Slide 5
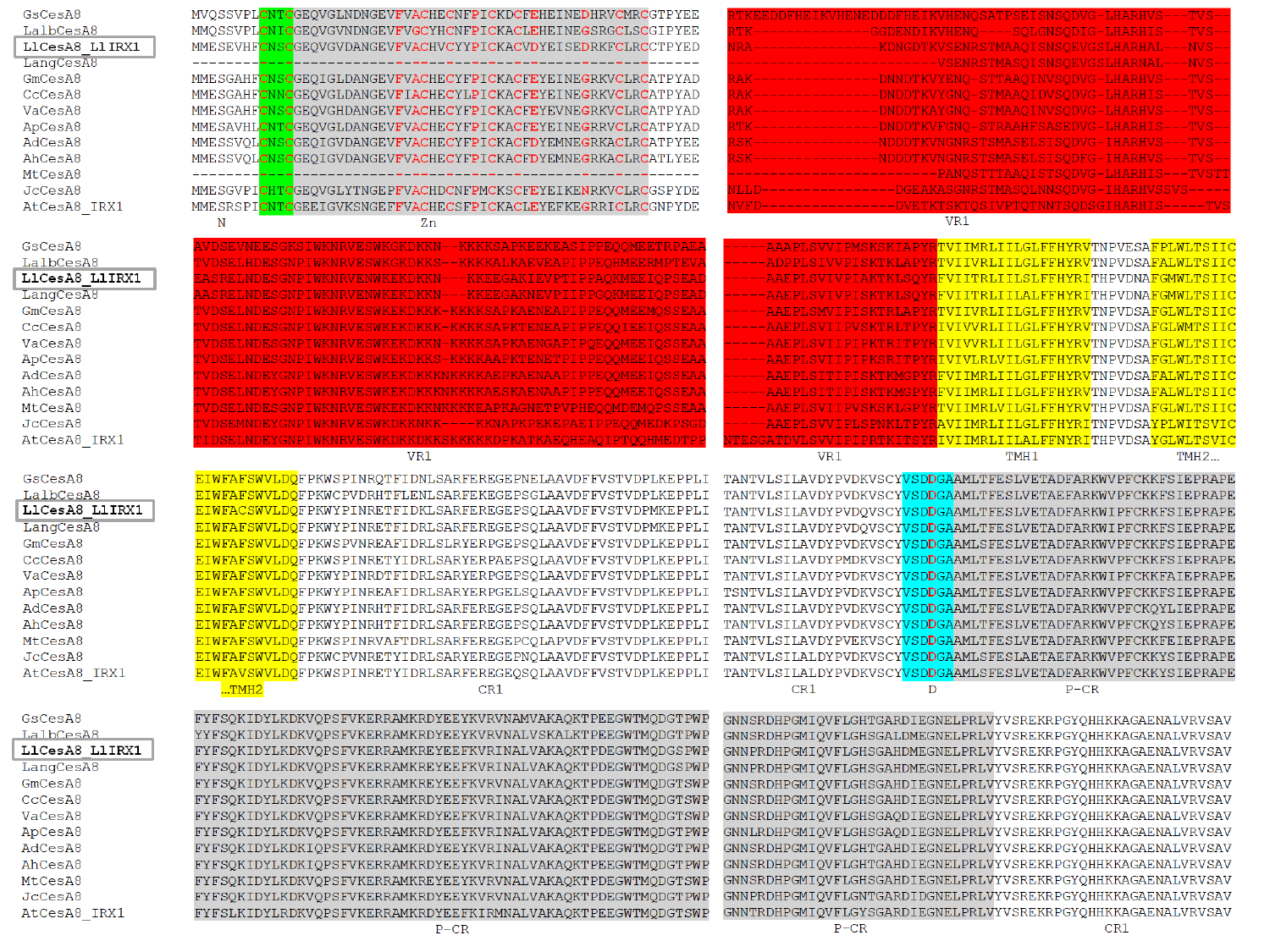

## Slide 6
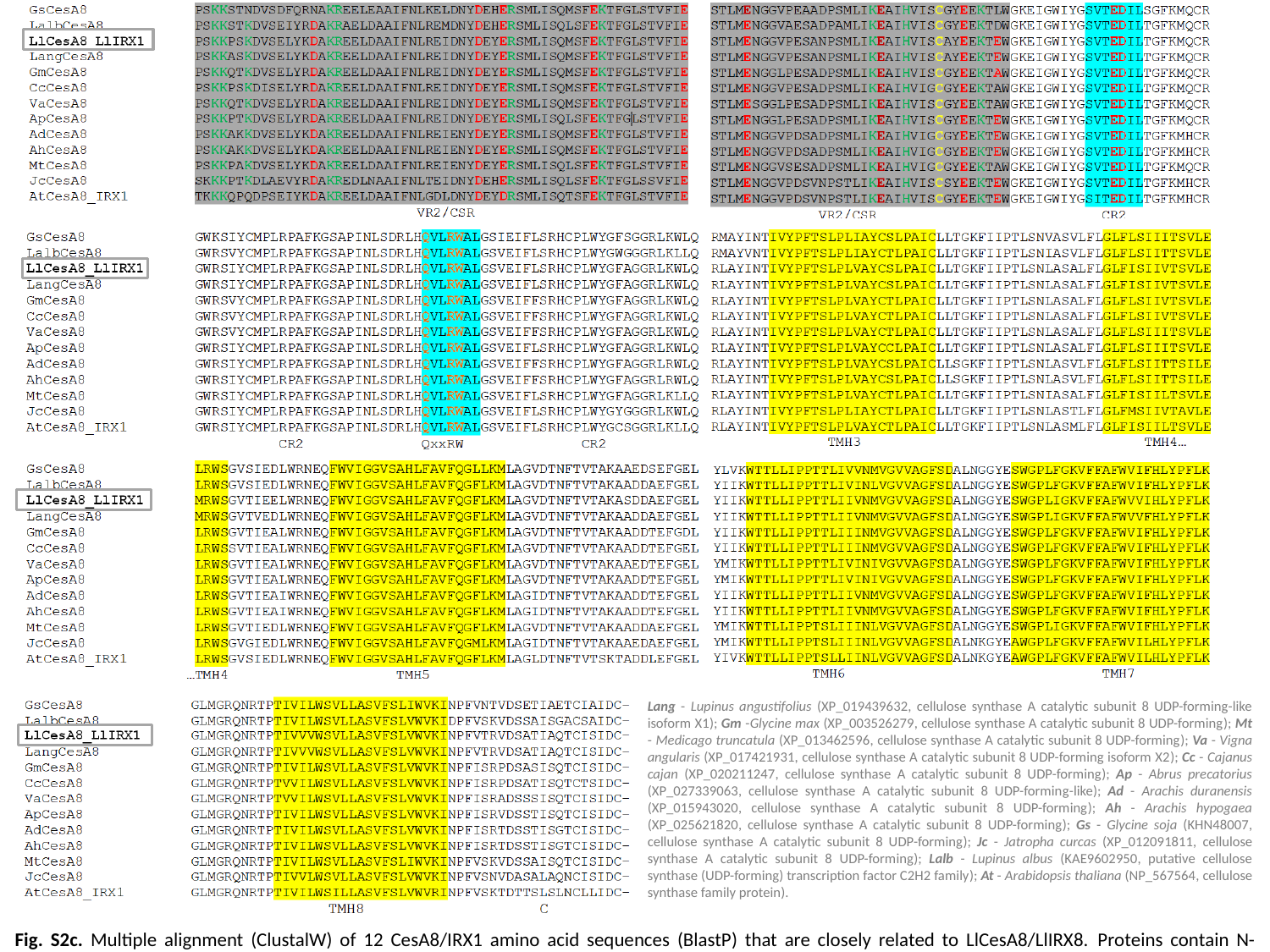

Lang - Lupinus angustifolius (XP_019439632, cellulose synthase A catalytic subunit 8 UDP-forming-like isoform X1); Gm -Glycine max (XP_003526279, cellulose synthase A catalytic subunit 8 UDP-forming); Mt - Medicago truncatula (XP_013462596, cellulose synthase A catalytic subunit 8 UDP-forming); Va - Vigna angularis (XP_017421931, cellulose synthase A catalytic subunit 8 UDP-forming isoform X2); Cc - Cajanus cajan (XP_020211247, cellulose synthase A catalytic subunit 8 UDP-forming); Ap - Abrus precatorius (XP_027339063, cellulose synthase A catalytic subunit 8 UDP-forming-like); Ad - Arachis duranensis (XP_015943020, cellulose synthase A catalytic subunit 8 UDP-forming); Ah - Arachis hypogaea (XP_025621820, cellulose synthase A catalytic subunit 8 UDP-forming); Gs - Glycine soja (KHN48007, cellulose synthase A catalytic subunit 8 UDP-forming); Jc - Jatropha curcas (XP_012091811, cellulose synthase A catalytic subunit 8 UDP-forming); Lalb - Lupinus albus (KAE9602950, putative cellulose synthase (UDP-forming) transcription factor C2H2 family); At - Arabidopsis thaliana (NP_567564, cellulose synthase family protein).
Fig. S2c. Multiple alignment (ClustalW) of 12 CesA8/IRX1 amino acid sequences (BlastP) that are closely related to LlCesA8/LlIRX8. Proteins contain N-terminus; globular/soluble central domain (CD) and the C-terminus. Short N-terminus (N) is prior to the Zinc-binding domain (Zn) (medium gray). This Zn domain contains strictly conserved CxxC motif (green box and red letters) beginning amino acids: CxxCx12FxACxxCxxPxCxxCxExxxxxDxxxCxxC, where x is any amino acid. Within the N-terminus is also a region rich in acidic amino acids designated as a hypervariable region (VR1, red box). Following the N-terminal domains are two transmembrane domains (TMH1/2, yellow boxes). The CD contains variable region 2 (VR2, dark grey) composed mostly of the Class Specific Region (CSR) which on either side is flanked by conserved regions: CR1 [with the Plant Conserved Region (P-CR) in the middle highlighted in light grey)] and CR2. The four recognized catalytic motifs are marked as blue boxes, with the D, DxD, and D residues in bold red and the QxxRW residues in bold orange. The numerous basic residues of Arg (R) and Lys (K) (green leterrs) and acidic residues of Asp (D) and Glu (E) (red letters) of the VR2/CSR, as well as the conserved Cys (C, yellow letters) are conserved across species in an isoform-specific manner [43]. The C-terminus contains six transmembrane domains (THM 3-8) and remaining protein after the last TM helix marked as C.

## Slide 7
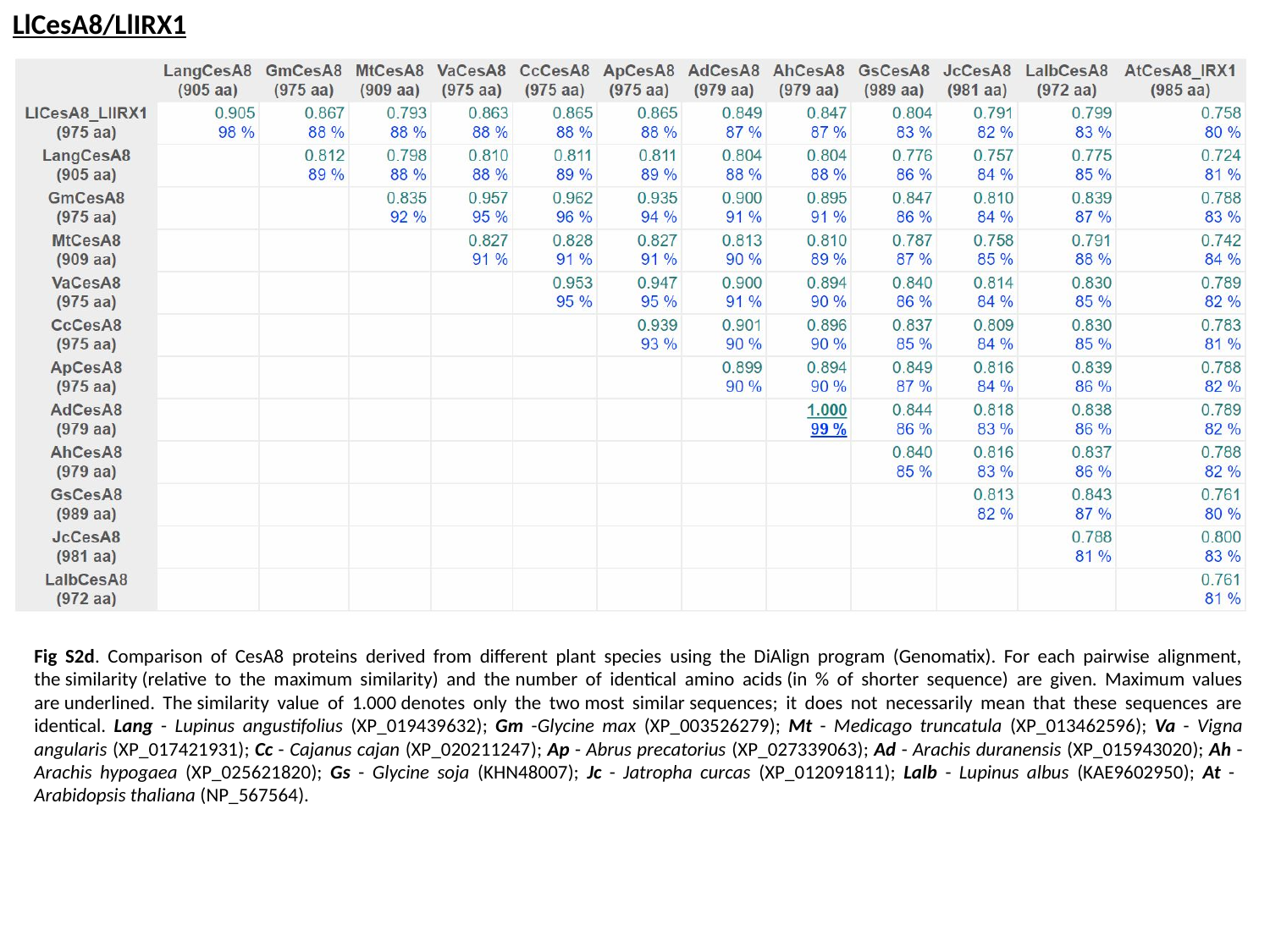

LlCesA8/LlIRX1
Fig S2d. Comparison of CesA8 proteins derived from different plant species using the DiAlign program (Genomatix). For each pairwise alignment, the similarity (relative to the maximum similarity) and the number of identical amino acids (in % of shorter sequence) are given. Maximum values are underlined. The similarity value of 1.000 denotes only the two most similar sequences; it does not necessarily mean that these sequences are identical. Lang - Lupinus angustifolius (XP_019439632); Gm -Glycine max (XP_003526279); Mt - Medicago truncatula (XP_013462596); Va - Vigna angularis (XP_017421931); Cc - Cajanus cajan (XP_020211247); Ap - Abrus precatorius (XP_027339063); Ad - Arachis duranensis (XP_015943020); Ah - Arachis hypogaea (XP_025621820); Gs - Glycine soja (KHN48007); Jc - Jatropha curcas (XP_012091811); Lalb - Lupinus albus (KAE9602950); At - Arabidopsis thaliana (NP_567564).

## Slide 8
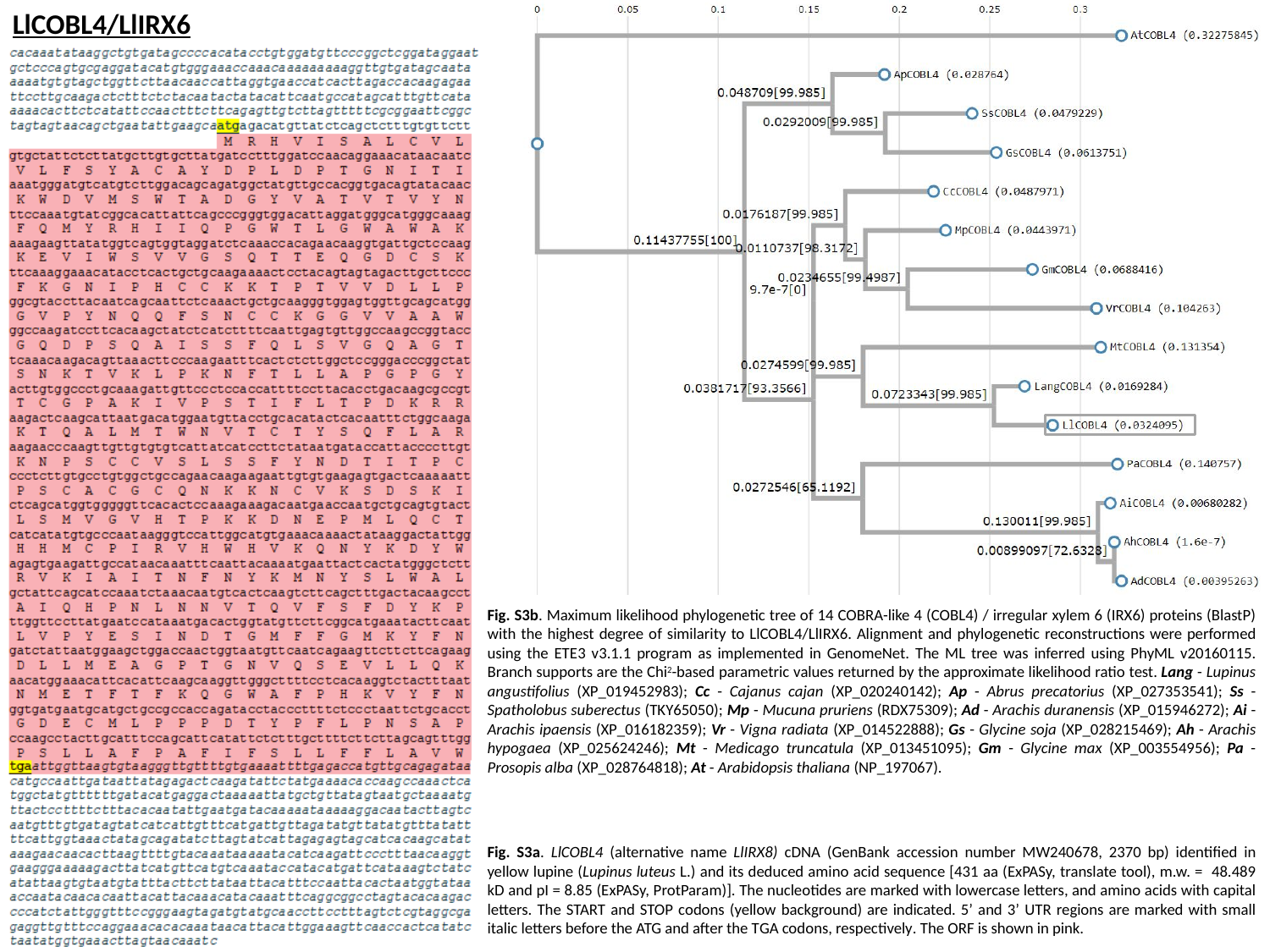

LlCOBL4/LlIRX6
Fig. S3b. Maximum likelihood phylogenetic tree of 14 COBRA-like 4 (COBL4) / irregular xylem 6 (IRX6) proteins (BlastP) with the highest degree of similarity to LlCOBL4/LlIRX6. Alignment and phylogenetic reconstructions were performed using the ETE3 v3.1.1 program as implemented in GenomeNet. The ML tree was inferred using PhyML v20160115. Branch supports are the Chi2-based parametric values returned by the approximate likelihood ratio test. Lang - Lupinus angustifolius (XP_019452983); Cc - Cajanus cajan (XP_020240142); Ap - Abrus precatorius (XP_027353541); Ss - Spatholobus suberectus (TKY65050); Mp - Mucuna pruriens (RDX75309); Ad - Arachis duranensis (XP_015946272); Ai - Arachis ipaensis (XP_016182359); Vr - Vigna radiata (XP_014522888); Gs - Glycine soja (XP_028215469); Ah - Arachis hypogaea (XP_025624246); Mt - Medicago truncatula (XP_013451095); Gm - Glycine max (XP_003554956); Pa - Prosopis alba (XP_028764818); At - Arabidopsis thaliana (NP_197067).
Fig. S3a. LlCOBL4 (alternative name LlIRX8) cDNA (GenBank accession number MW240678, 2370 bp) identified in yellow lupine (Lupinus luteus L.) and its deduced amino acid sequence [431 aa (ExPASy, translate tool), m.w. = 48.489 kD and pI = 8.85 (ExPASy, ProtParam)]. The nucleotides are marked with lowercase letters, and amino acids with capital letters. The START and STOP codons (yellow background) are indicated. 5’ and 3’ UTR regions are marked with small italic letters before the ATG and after the TGA codons, respectively. The ORF is shown in pink.

## Slide 9
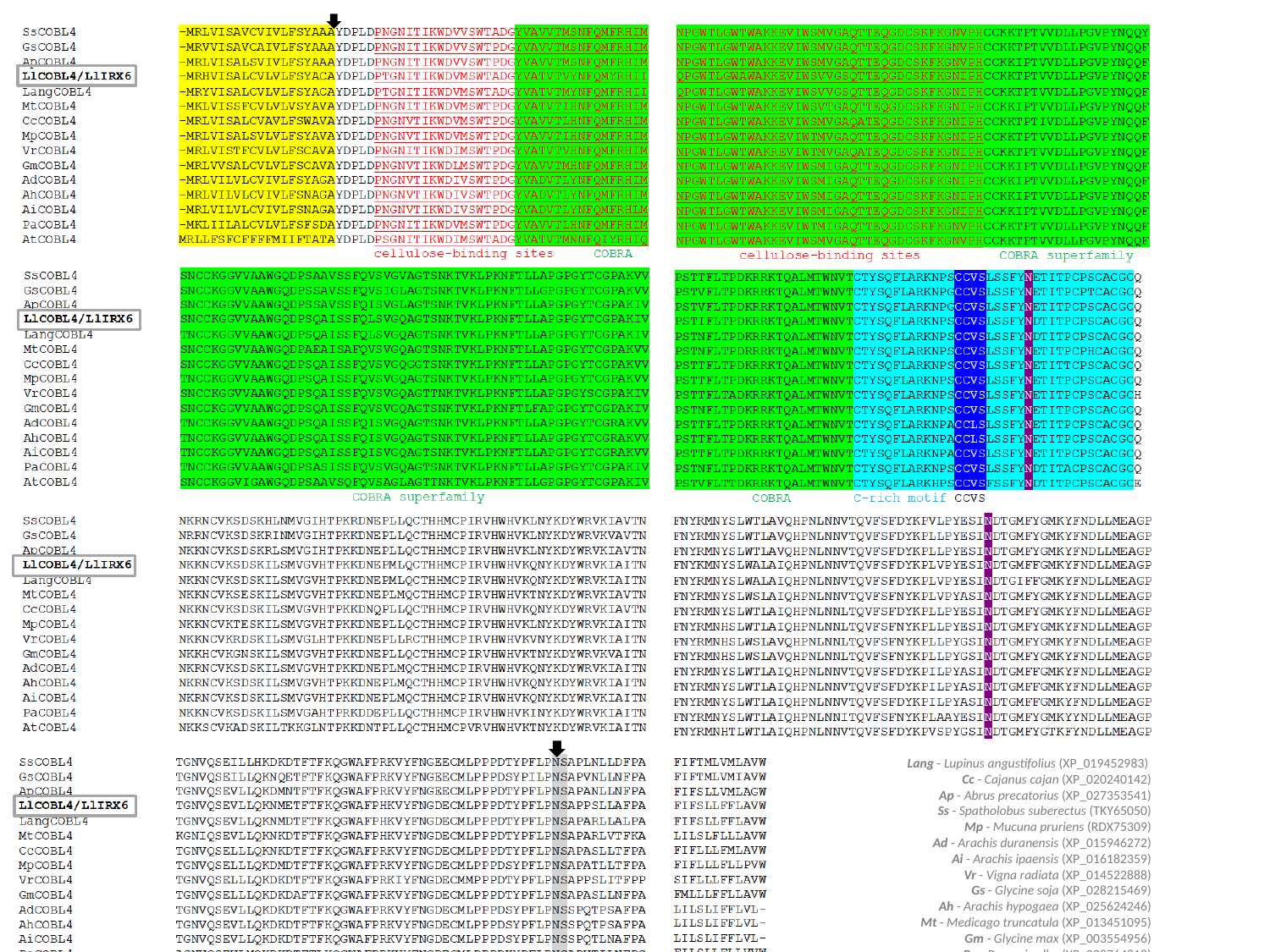

Lang - Lupinus angustifolius (XP_019452983)
Cc - Cajanus cajan (XP_020240142)
Ap - Abrus precatorius (XP_027353541)
Ss - Spatholobus suberectus (TKY65050)
Mp - Mucuna pruriens (RDX75309)
Ad - Arachis duranensis (XP_015946272)
Ai - Arachis ipaensis (XP_016182359)
Vr - Vigna radiata (XP_014522888)
Gs - Glycine soja (XP_028215469)
Ah - Arachis hypogaea (XP_025624246)
Mt - Medicago truncatula (XP_013451095)
Gm - Glycine max (XP_003554956)
Pa - Prosopis alba (XP_028764818)
At - Arabidopsis thaliana (NP_197067)
Fig. S3c. Multiple alignment (ClustalW) of 14 COBL4/IRX6 amino acid sequences (BlastP) that are closely related to LlCOBL4/LlIRX6. Conserved domain characteristic to COBRA superfamily is marked in green background. The N-terminal predicted signal peptide is marked yellow, with cleavage site marked by black arrow. The underlined sequences with red letters show the putative cellulose-binding sites. The light blue sequences denote the Cys-rich (CCVS) motif characteristic for all COBL proteins, while CCVS amino acids are marked in dark blue background and white letters. Two conserved consensus N-glycosylation sites are indicated by a white letters on violet backgrounds. Locus corresponds to the predicted cleavage ω sites in the C terminus is indicated in middium grey background and black arrow.

## Slide 10
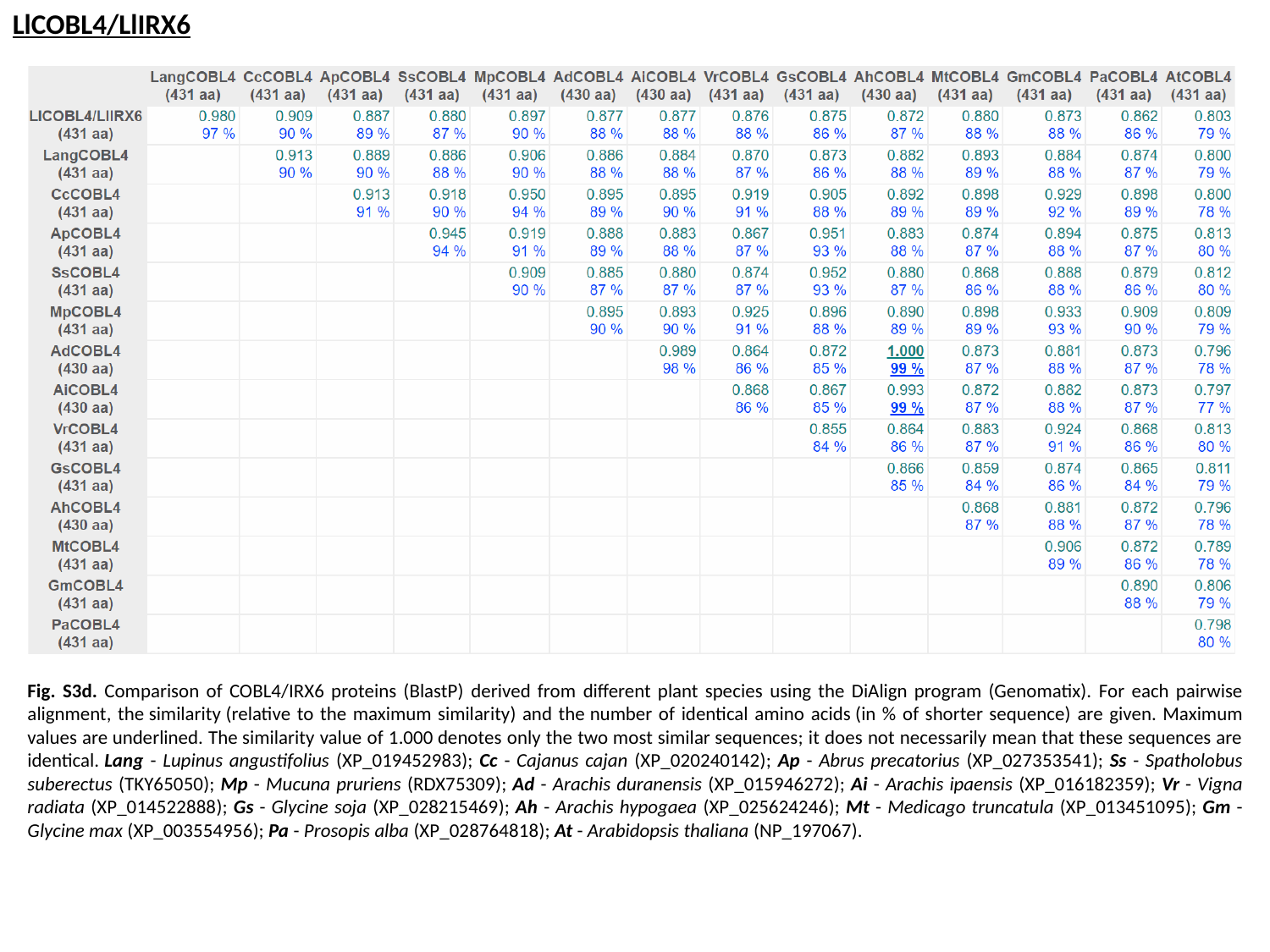

LlCOBL4/LlIRX6
Fig. S3d. Comparison of COBL4/IRX6 proteins (BlastP) derived from different plant species using the DiAlign program (Genomatix). For each pairwise alignment, the similarity (relative to the maximum similarity) and the number of identical amino acids (in % of shorter sequence) are given. Maximum values are underlined. The similarity value of 1.000 denotes only the two most similar sequences; it does not necessarily mean that these sequences are identical. Lang - Lupinus angustifolius (XP_019452983); Cc - Cajanus cajan (XP_020240142); Ap - Abrus precatorius (XP_027353541); Ss - Spatholobus suberectus (TKY65050); Mp - Mucuna pruriens (RDX75309); Ad - Arachis duranensis (XP_015946272); Ai - Arachis ipaensis (XP_016182359); Vr - Vigna radiata (XP_014522888); Gs - Glycine soja (XP_028215469); Ah - Arachis hypogaea (XP_025624246); Mt - Medicago truncatula (XP_013451095); Gm - Glycine max (XP_003554956); Pa - Prosopis alba (XP_028764818); At - Arabidopsis thaliana (NP_197067).

## Slide 11
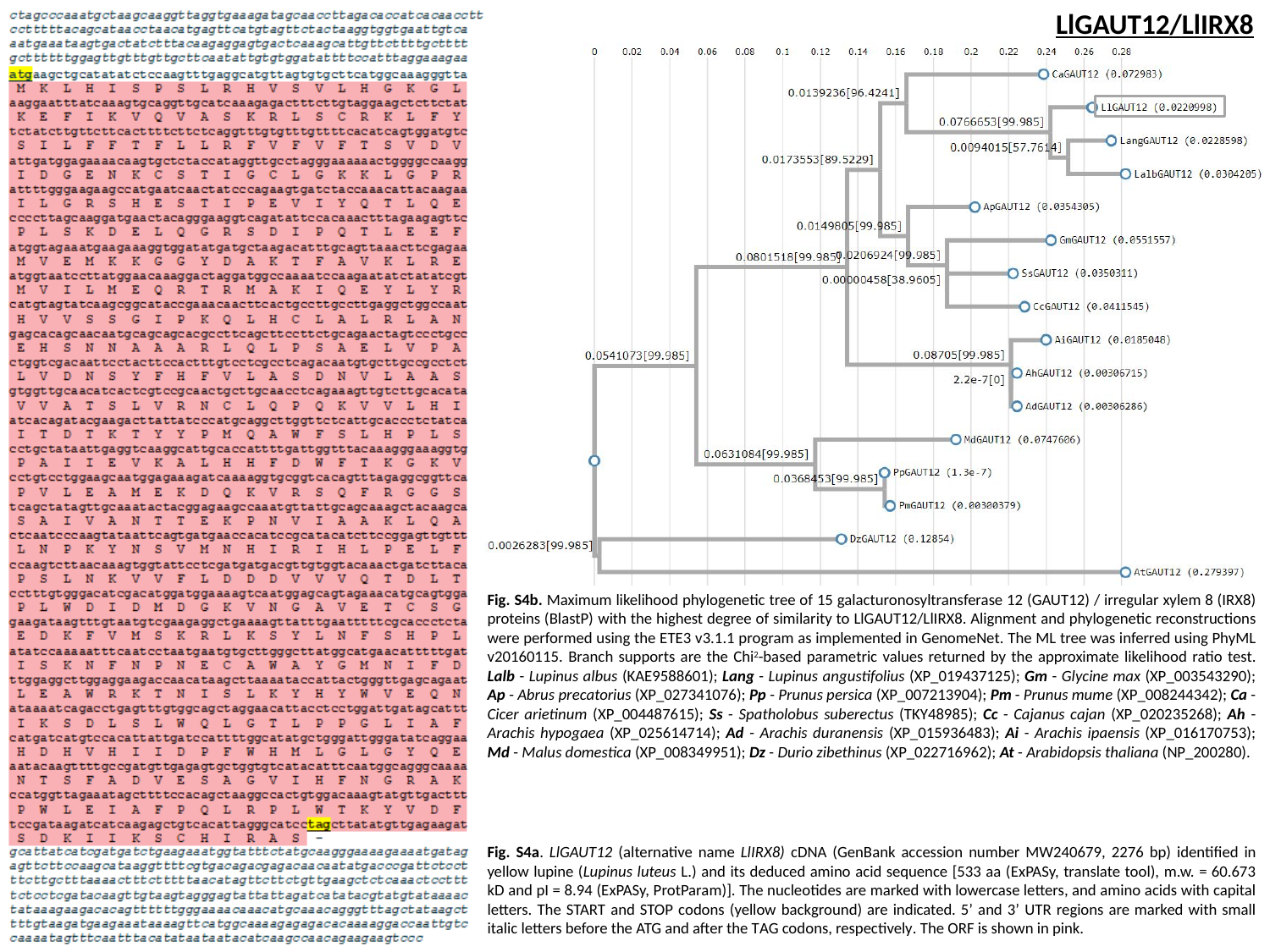

LlGAUT12/LlIRX8
Fig. S4b. Maximum likelihood phylogenetic tree of 15 galacturonosyltransferase 12 (GAUT12) / irregular xylem 8 (IRX8) proteins (BlastP) with the highest degree of similarity to LlGAUT12/LlIRX8. Alignment and phylogenetic reconstructions were performed using the ETE3 v3.1.1 program as implemented in GenomeNet. The ML tree was inferred using PhyML v20160115. Branch supports are the Chi2-based parametric values returned by the approximate likelihood ratio test. Lalb - Lupinus albus (KAE9588601); Lang - Lupinus angustifolius (XP_019437125); Gm - Glycine max (XP_003543290); Ap - Abrus precatorius (XP_027341076); Pp - Prunus persica (XP_007213904); Pm - Prunus mume (XP_008244342); Ca - Cicer arietinum (XP_004487615); Ss - Spatholobus suberectus (TKY48985); Cc - Cajanus cajan (XP_020235268); Ah - Arachis hypogaea (XP_025614714); Ad - Arachis duranensis (XP_015936483); Ai - Arachis ipaensis (XP_016170753); Md - Malus domestica (XP_008349951); Dz - Durio zibethinus (XP_022716962); At - Arabidopsis thaliana (NP_200280).
Fig. S4a. LlGAUT12 (alternative name LlIRX8) cDNA (GenBank accession number MW240679, 2276 bp) identified in yellow lupine (Lupinus luteus L.) and its deduced amino acid sequence [533 aa (ExPASy, translate tool), m.w. = 60.673 kD and pI = 8.94 (ExPASy, ProtParam)]. The nucleotides are marked with lowercase letters, and amino acids with capital letters. The START and STOP codons (yellow background) are indicated. 5’ and 3’ UTR regions are marked with small italic letters before the ATG and after the TAG codons, respectively. The ORF is shown in pink.

## Slide 12
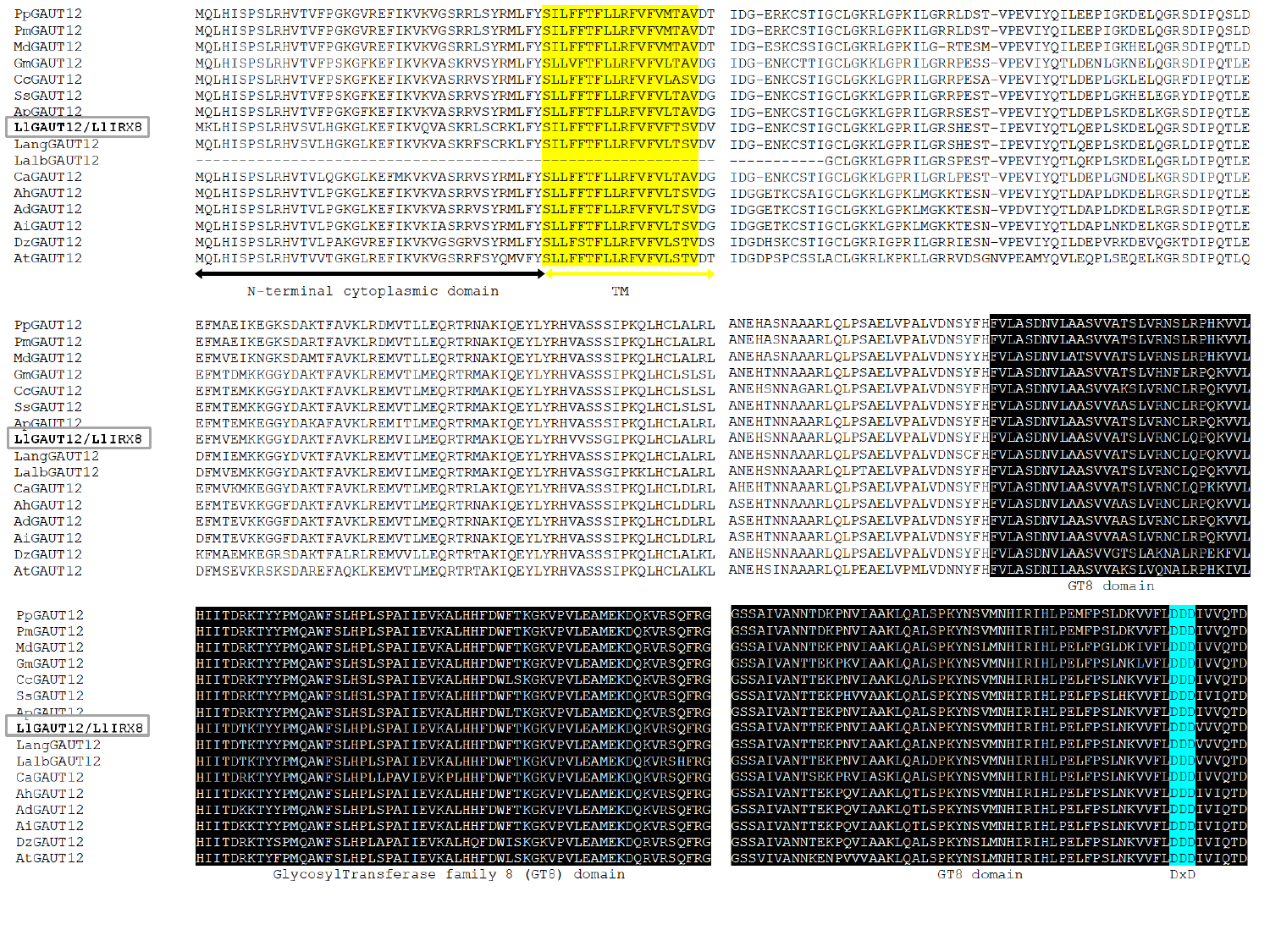

## Slide 13
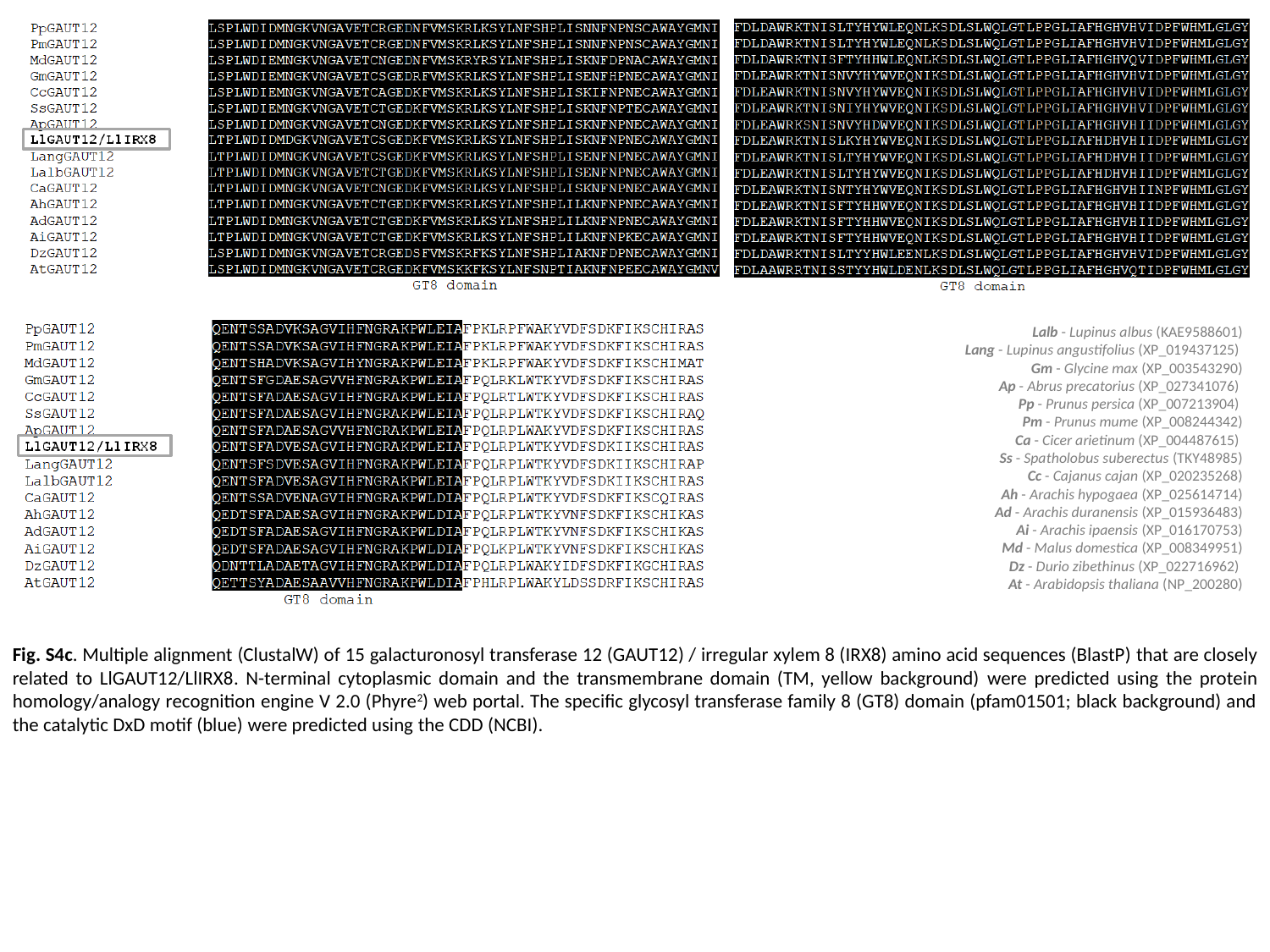

Lalb - Lupinus albus (KAE9588601)
Lang - Lupinus angustifolius (XP_019437125)
Gm - Glycine max (XP_003543290)
Ap - Abrus precatorius (XP_027341076)
Pp - Prunus persica (XP_007213904)
Pm - Prunus mume (XP_008244342)
Ca - Cicer arietinum (XP_004487615)
Ss - Spatholobus suberectus (TKY48985)
Cc - Cajanus cajan (XP_020235268)
Ah - Arachis hypogaea (XP_025614714)
Ad - Arachis duranensis (XP_015936483)
Ai - Arachis ipaensis (XP_016170753)
Md - Malus domestica (XP_008349951)
Dz - Durio zibethinus (XP_022716962)
At - Arabidopsis thaliana (NP_200280)
Fig. S4c. Multiple alignment (ClustalW) of 15 galacturonosyl transferase 12 (GAUT12) / irregular xylem 8 (IRX8) amino acid sequences (BlastP) that are closely related to LlGAUT12/LlIRX8. N-terminal cytoplasmic domain and the transmembrane domain (TM, yellow background) were predicted using the protein homology/analogy recognition engine V 2.0 (Phyre2) web portal. The specific glycosyl transferase family 8 (GT8) domain (pfam01501; black background) and the catalytic DxD motif (blue) were predicted using the CDD (NCBI).

## Slide 14
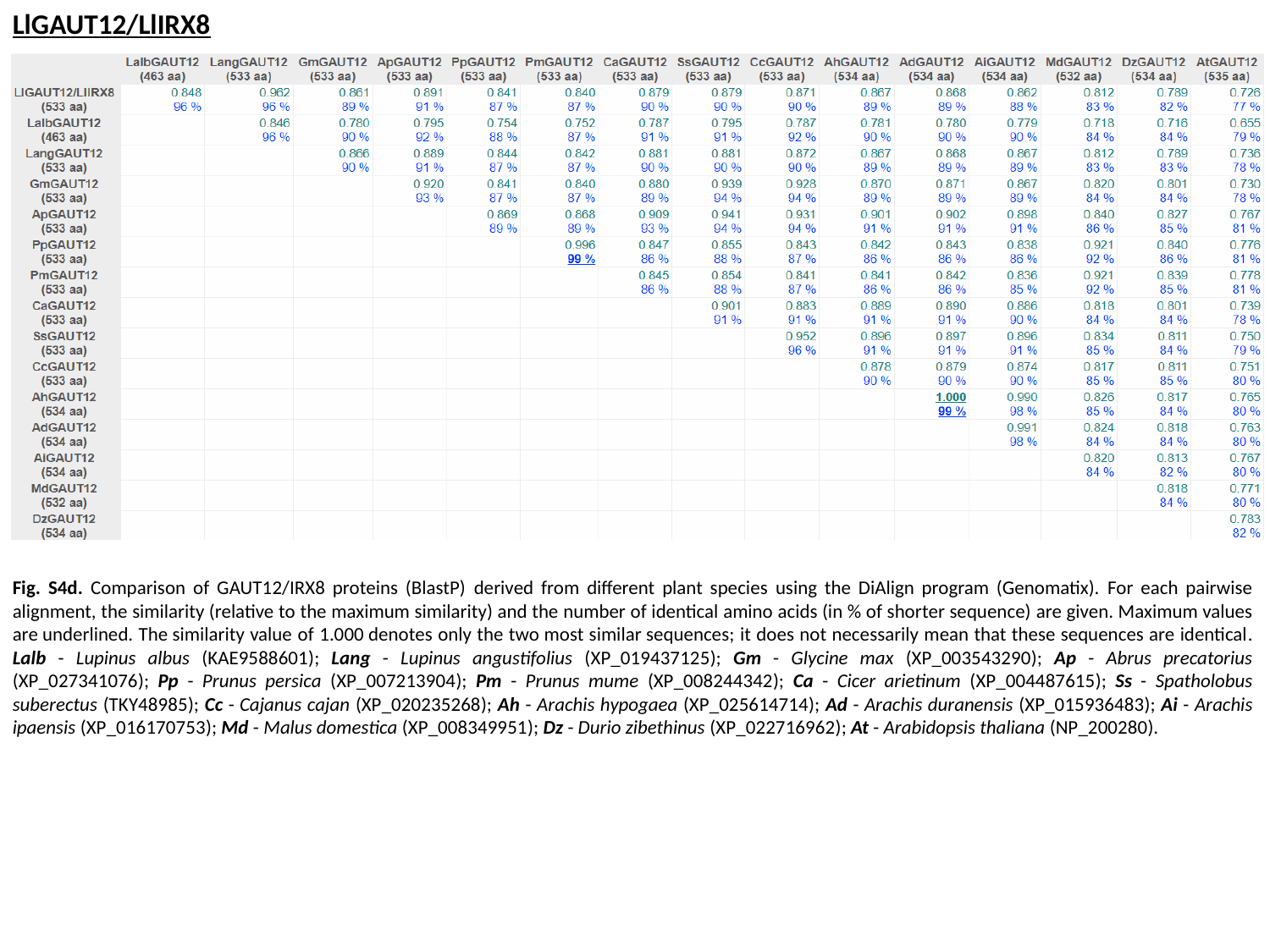

LlGAUT12/LlIRX8
Fig. S4d. Comparison of GAUT12/IRX8 proteins (BlastP) derived from different plant species using the DiAlign program (Genomatix). For each pairwise alignment, the similarity (relative to the maximum similarity) and the number of identical amino acids (in % of shorter sequence) are given. Maximum values are underlined. The similarity value of 1.000 denotes only the two most similar sequences; it does not necessarily mean that these sequences are identical. Lalb - Lupinus albus (KAE9588601); Lang - Lupinus angustifolius (XP_019437125); Gm - Glycine max (XP_003543290); Ap - Abrus precatorius (XP_027341076); Pp - Prunus persica (XP_007213904); Pm - Prunus mume (XP_008244342); Ca - Cicer arietinum (XP_004487615); Ss - Spatholobus suberectus (TKY48985); Cc - Cajanus cajan (XP_020235268); Ah - Arachis hypogaea (XP_025614714); Ad - Arachis duranensis (XP_015936483); Ai - Arachis ipaensis (XP_016170753); Md - Malus domestica (XP_008349951); Dz - Durio zibethinus (XP_022716962); At - Arabidopsis thaliana (NP_200280).

## Slide 15
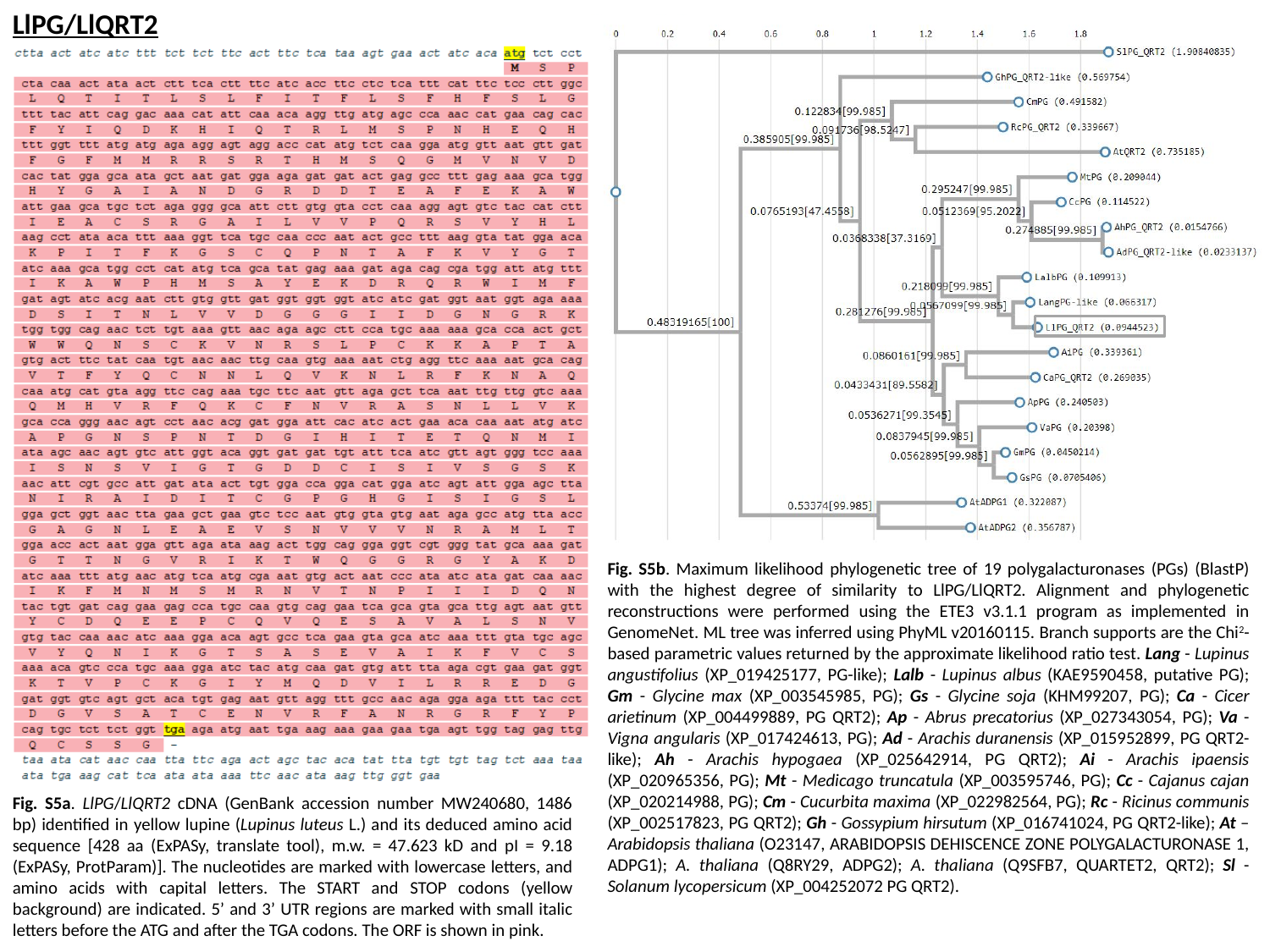

LlPG/LlQRT2
Fig. S5b. Maximum likelihood phylogenetic tree of 19 polygalacturonases (PGs) (BlastP) with the highest degree of similarity to LlPG/LlQRT2. Alignment and phylogenetic reconstructions were performed using the ETE3 v3.1.1 program as implemented in GenomeNet. ML tree was inferred using PhyML v20160115. Branch supports are the Chi2-based parametric values returned by the approximate likelihood ratio test. Lang - Lupinus angustifolius (XP_019425177, PG-like); Lalb - Lupinus albus (KAE9590458, putative PG); Gm - Glycine max (XP_003545985, PG); Gs - Glycine soja (KHM99207, PG); Ca - Cicer arietinum (XP_004499889, PG QRT2); Ap - Abrus precatorius (XP_027343054, PG); Va - Vigna angularis (XP_017424613, PG); Ad - Arachis duranensis (XP_015952899, PG QRT2-like); Ah - Arachis hypogaea (XP_025642914, PG QRT2); Ai - Arachis ipaensis (XP_020965356, PG); Mt - Medicago truncatula (XP_003595746, PG); Cc - Cajanus cajan (XP_020214988, PG); Cm - Cucurbita maxima (XP_022982564, PG); Rc - Ricinus communis (XP_002517823, PG QRT2); Gh - Gossypium hirsutum (XP_016741024, PG QRT2-like); At – Arabidopsis thaliana (O23147, ARABIDOPSIS DEHISCENCE ZONE POLYGALACTURONASE 1, ADPG1); A. thaliana (Q8RY29, ADPG2); A. thaliana (Q9SFB7, QUARTET2, QRT2); Sl - Solanum lycopersicum (XP_004252072 PG QRT2).
Fig. S5a. LlPG/LlQRT2 cDNA (GenBank accession number MW240680, 1486 bp) identified in yellow lupine (Lupinus luteus L.) and its deduced amino acid sequence [428 aa (ExPASy, translate tool), m.w. = 47.623 kD and pI = 9.18 (ExPASy, ProtParam)]. The nucleotides are marked with lowercase letters, and amino acids with capital letters. The START and STOP codons (yellow background) are indicated. 5’ and 3’ UTR regions are marked with small italic letters before the ATG and after the TGA codons. The ORF is shown in pink.

## Slide 16
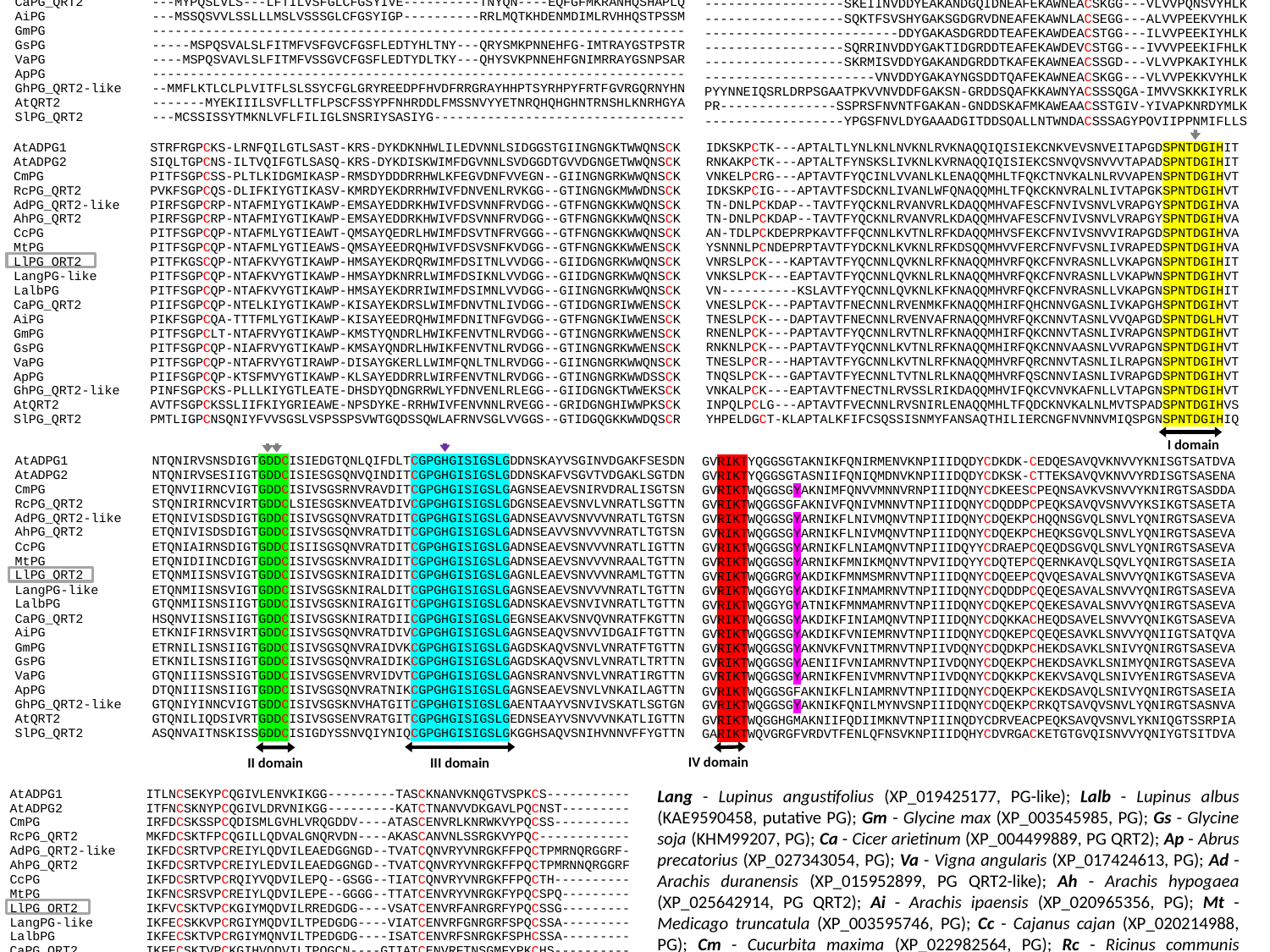

I domain
IV domain
II domain
III domain
Lang - Lupinus angustifolius (XP_019425177, PG-like); Lalb - Lupinus albus (KAE9590458, putative PG); Gm - Glycine max (XP_003545985, PG); Gs - Glycine soja (KHM99207, PG); Ca - Cicer arietinum (XP_004499889, PG QRT2); Ap - Abrus precatorius (XP_027343054, PG); Va - Vigna angularis (XP_017424613, PG); Ad - Arachis duranensis (XP_015952899, PG QRT2-like); Ah - Arachis hypogaea (XP_025642914, PG QRT2); Ai - Arachis ipaensis (XP_020965356, PG); Mt - Medicago truncatula (XP_003595746, PG); Cc - Cajanus cajan (XP_020214988, PG); Cm - Cucurbita maxima (XP_022982564, PG); Rc - Ricinus communis (XP_002517823, PG QRT2); Gh - Gossypium hirsutum (XP_016741024, PG QRT2-like); At – Arabidopsis thaliana (O23147, ARABIDOPSIS DEHISCENCE ZONE POLYGALACTURONASE 1, ADPG1); A. thaliana (Q8RY29, ADPG2); A. thaliana (Q9SFB7, QUARTET2, QRT2); Sl - Solanum lycopersicum (XP_004252072 PG QRT2).
Fig. S5c. Multiple sequence alignment (ClustalW) of different polygalacturonases (PGs, BlastP) showing the highest similarity to LlPG/LlQRT2. Four typical conserved domains of PGs, referred to as domains I, II, III and IV (RIKT) were indicatated in yellow, green, blue and red, respectively. A tyrosine (Y) is marked in pink. 12 cysteine (C) residues are marked in red letters. Three aspartic acids in NTD and DD structures (domains I and II, respectively) are marked with gray arrows. The histidine residue (H) in domain III is marked with purple arrow.

## Slide 17
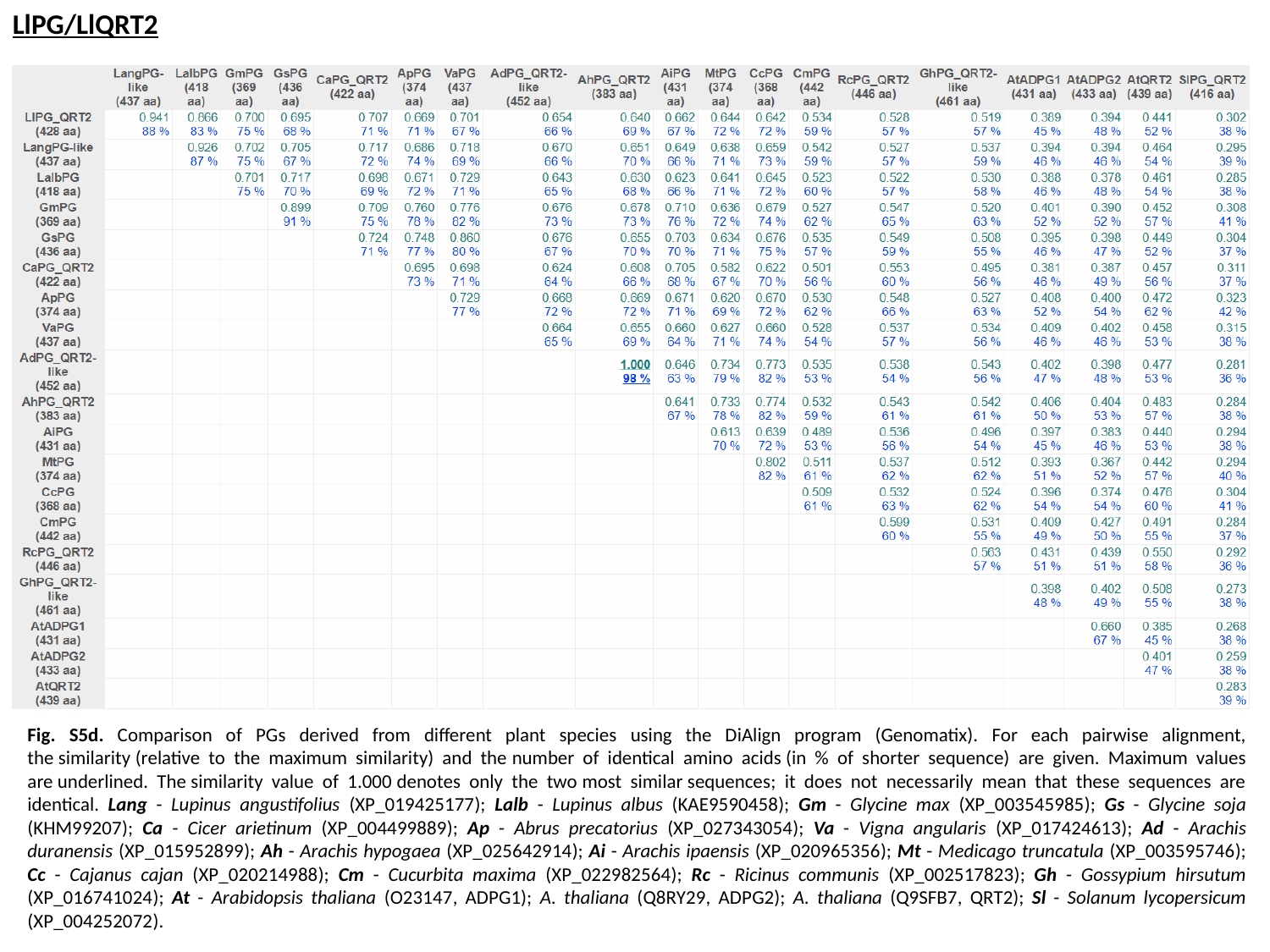

LlPG/LlQRT2
Fig. S5d. Comparison of PGs derived from different plant species using the DiAlign program (Genomatix). For each pairwise alignment, the similarity (relative to the maximum similarity) and the number of identical amino acids (in % of shorter sequence) are given. Maximum values are underlined. The similarity value of 1.000 denotes only the two most similar sequences; it does not necessarily mean that these sequences are identical. Lang - Lupinus angustifolius (XP_019425177); Lalb - Lupinus albus (KAE9590458); Gm - Glycine max (XP_003545985); Gs - Glycine soja (KHM99207); Ca - Cicer arietinum (XP_004499889); Ap - Abrus precatorius (XP_027343054); Va - Vigna angularis (XP_017424613); Ad - Arachis duranensis (XP_015952899); Ah - Arachis hypogaea (XP_025642914); Ai - Arachis ipaensis (XP_020965356); Mt - Medicago truncatula (XP_003595746); Cc - Cajanus cajan (XP_020214988); Cm - Cucurbita maxima (XP_022982564); Rc - Ricinus communis (XP_002517823); Gh - Gossypium hirsutum (XP_016741024); At - Arabidopsis thaliana (O23147, ADPG1); A. thaliana (Q8RY29, ADPG2); A. thaliana (Q9SFB7, QRT2); Sl - Solanum lycopersicum (XP_004252072).

## Slide 18
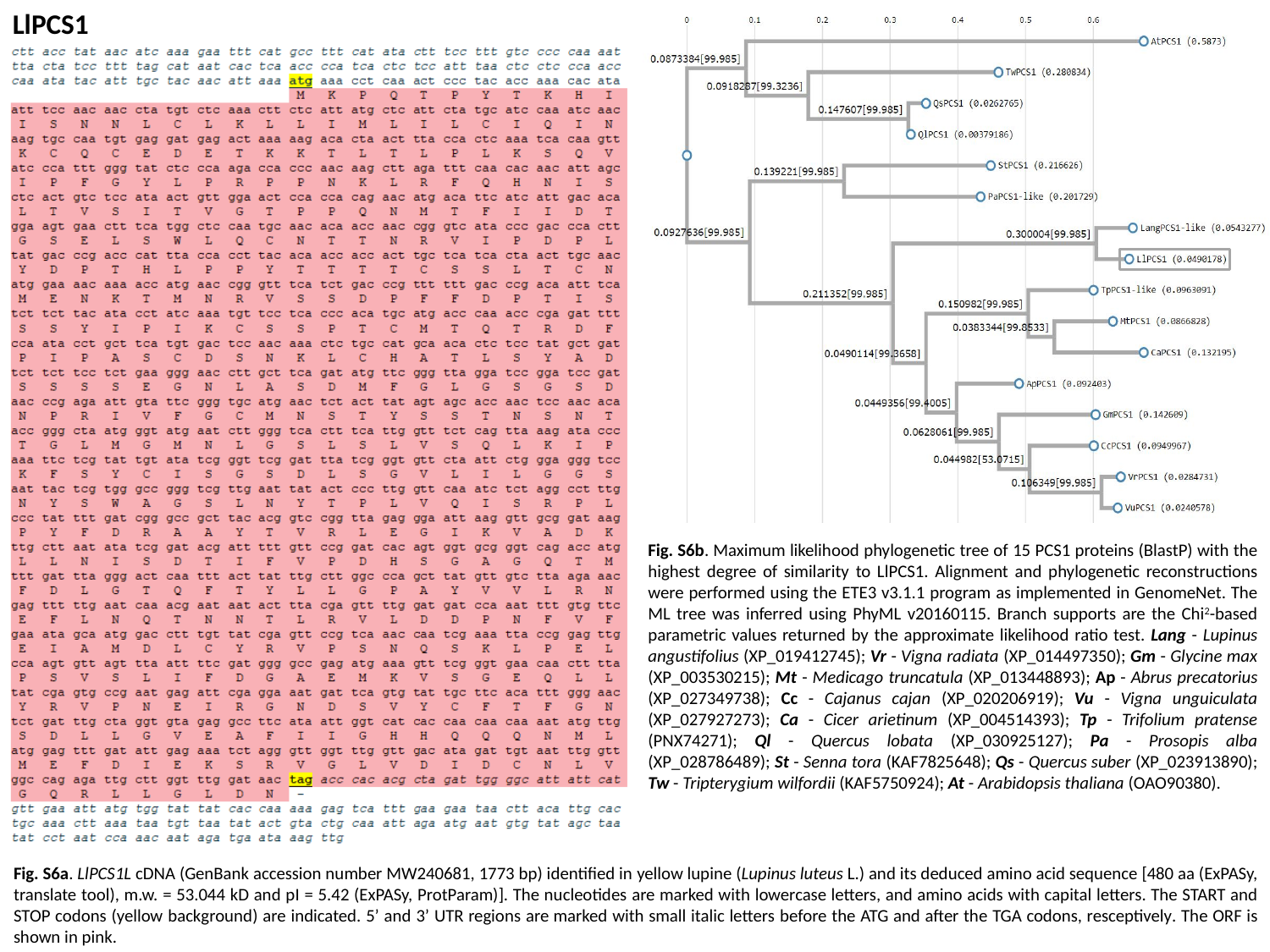

LlPCS1
Fig. S6b. Maximum likelihood phylogenetic tree of 15 PCS1 proteins (BlastP) with the highest degree of similarity to LlPCS1. Alignment and phylogenetic reconstructions were performed using the ETE3 v3.1.1 program as implemented in GenomeNet. The ML tree was inferred using PhyML v20160115. Branch supports are the Chi2-based parametric values returned by the approximate likelihood ratio test. Lang - Lupinus angustifolius (XP_019412745); Vr - Vigna radiata (XP_014497350); Gm - Glycine max (XP_003530215); Mt - Medicago truncatula (XP_013448893); Ap - Abrus precatorius (XP_027349738); Cc - Cajanus cajan (XP_020206919); Vu - Vigna unguiculata (XP_027927273); Ca - Cicer arietinum (XP_004514393); Tp - Trifolium pratense (PNX74271); Ql - Quercus lobata (XP_030925127); Pa - Prosopis alba (XP_028786489); St - Senna tora (KAF7825648); Qs - Quercus suber (XP_023913890); Tw - Tripterygium wilfordii (KAF5750924); At - Arabidopsis thaliana (OAO90380).
Fig. S6a. LlPCS1L cDNA (GenBank accession number MW240681, 1773 bp) identified in yellow lupine (Lupinus luteus L.) and its deduced amino acid sequence [480 aa (ExPASy, translate tool), m.w. = 53.044 kD and pI = 5.42 (ExPASy, ProtParam)]. The nucleotides are marked with lowercase letters, and amino acids with capital letters. The START and STOP codons (yellow background) are indicated. 5’ and 3’ UTR regions are marked with small italic letters before the ATG and after the TGA codons, resceptively. The ORF is shown in pink.

## Slide 19
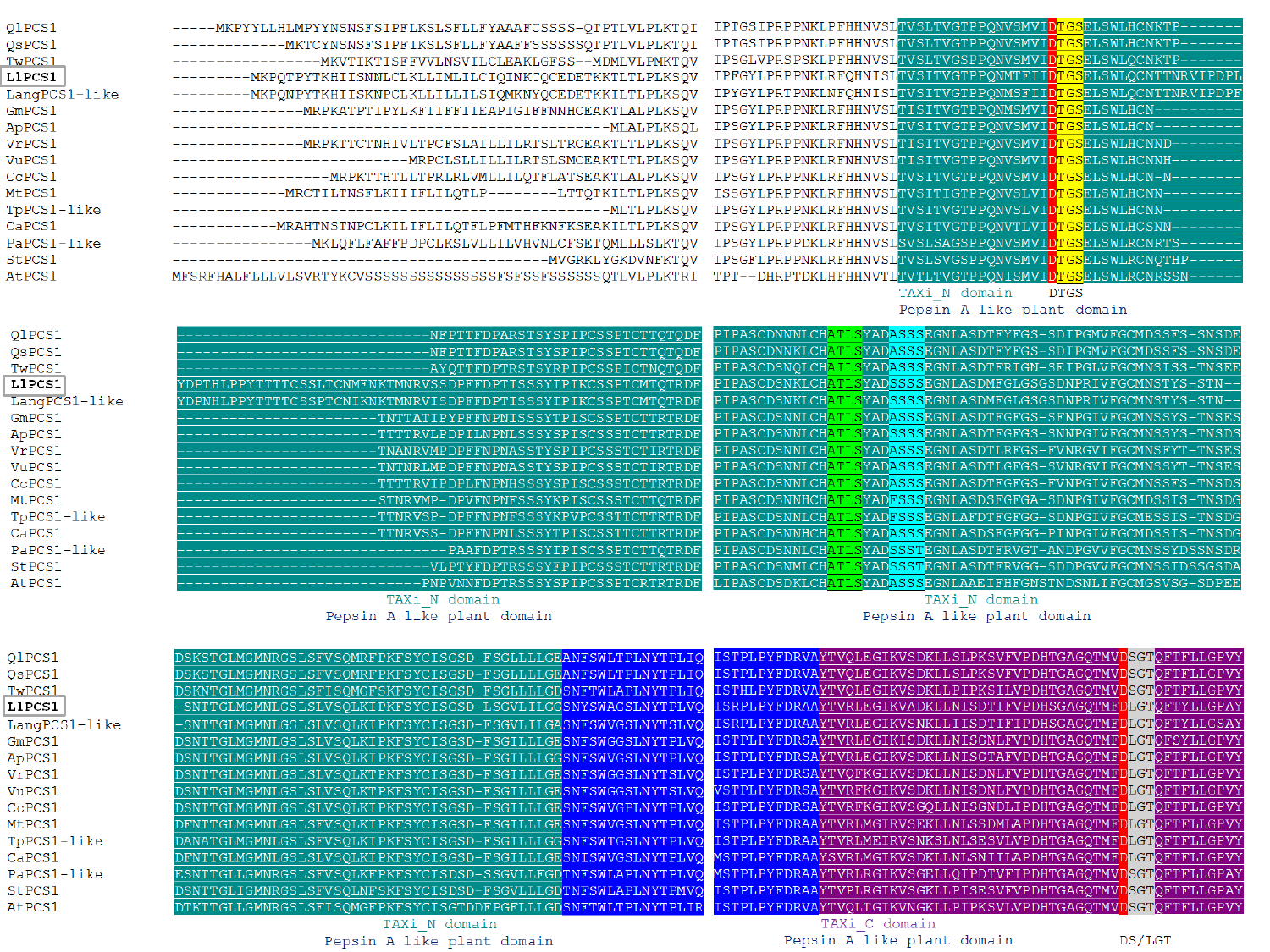

## Slide 20
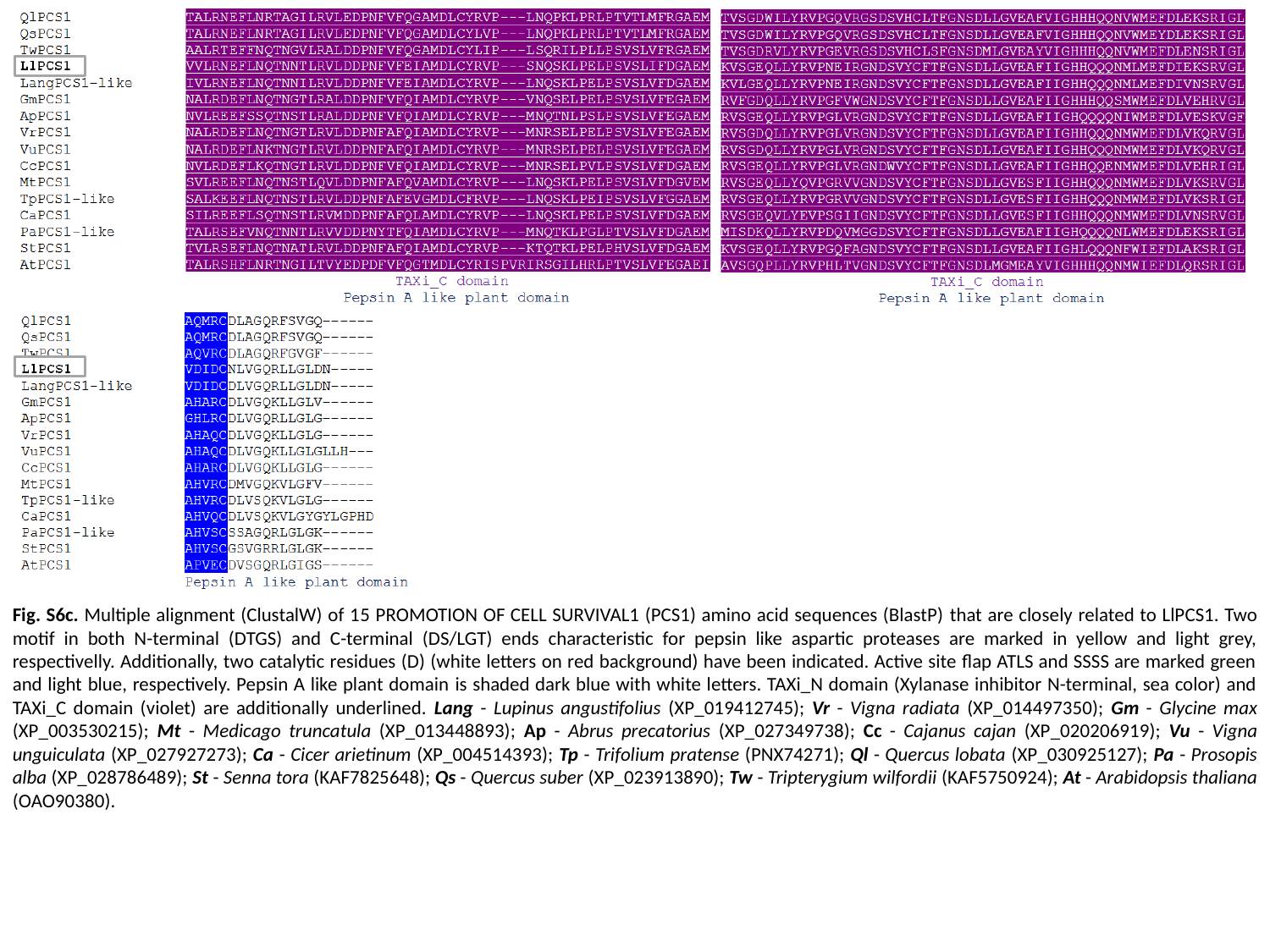

Fig. S6c. Multiple alignment (ClustalW) of 15 PROMOTION OF CELL SURVIVAL1 (PCS1) amino acid sequences (BlastP) that are closely related to LlPCS1. Two motif in both N-terminal (DTGS) and C-terminal (DS/LGT) ends characteristic for pepsin like aspartic proteases are marked in yellow and light grey, respectivelly. Additionally, two catalytic residues (D) (white letters on red background) have been indicated. Active site flap ATLS and SSSS are marked green and light blue, respectively. Pepsin A like plant domain is shaded dark blue with white letters. TAXi_N domain (Xylanase inhibitor N-terminal, sea color) and TAXi_C domain (violet) are additionally underlined. Lang - Lupinus angustifolius (XP_019412745); Vr - Vigna radiata (XP_014497350); Gm - Glycine max (XP_003530215); Mt - Medicago truncatula (XP_013448893); Ap - Abrus precatorius (XP_027349738); Cc - Cajanus cajan (XP_020206919); Vu - Vigna unguiculata (XP_027927273); Ca - Cicer arietinum (XP_004514393); Tp - Trifolium pratense (PNX74271); Ql - Quercus lobata (XP_030925127); Pa - Prosopis alba (XP_028786489); St - Senna tora (KAF7825648); Qs - Quercus suber (XP_023913890); Tw - Tripterygium wilfordii (KAF5750924); At - Arabidopsis thaliana (OAO90380).

## Slide 21
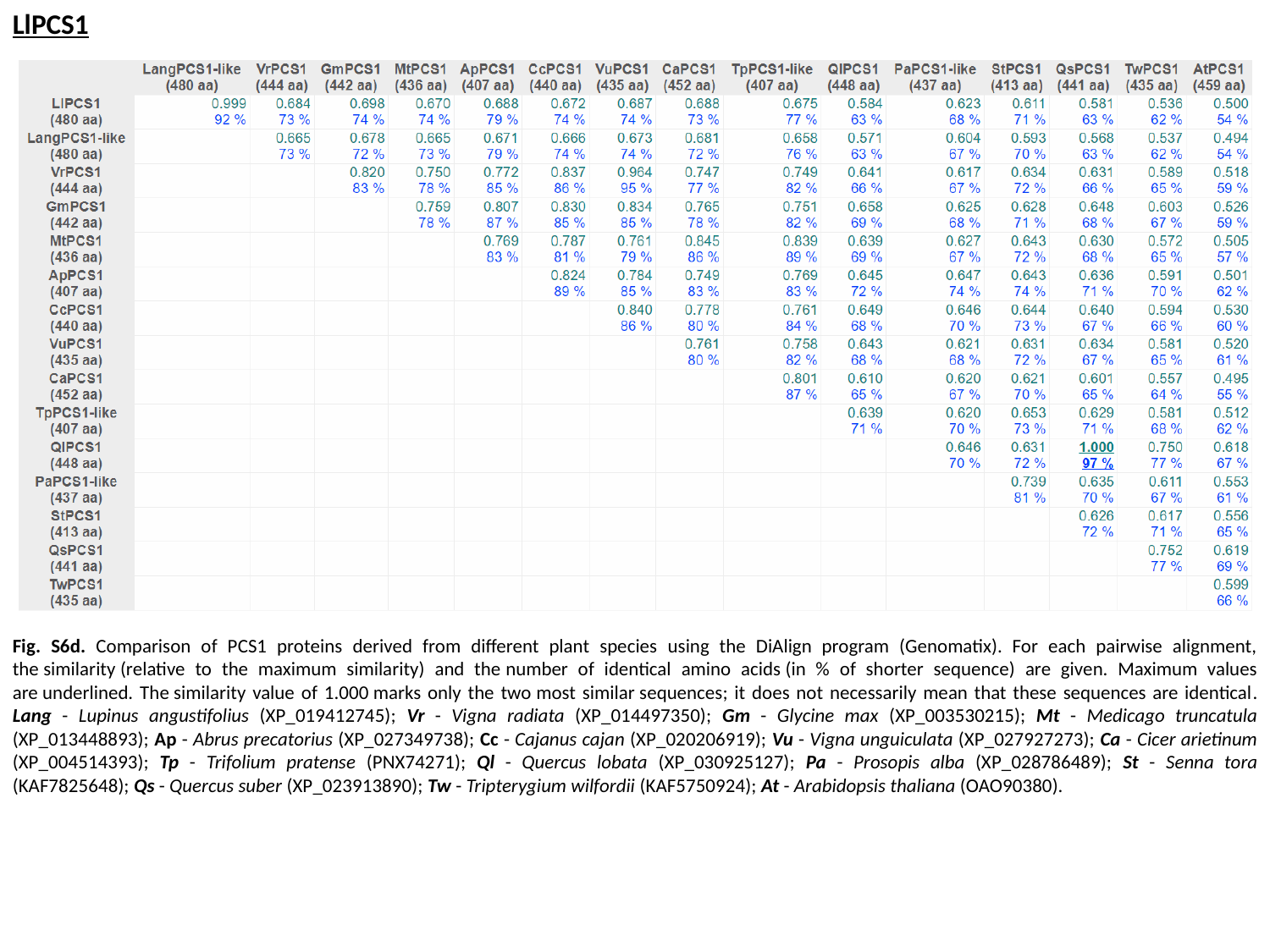

LlPCS1
Fig. S6d. Comparison of PCS1 proteins derived from different plant species using the DiAlign program (Genomatix). For each pairwise alignment, the similarity (relative to the maximum similarity) and the number of identical amino acids (in % of shorter sequence) are given. Maximum values are underlined. The similarity value of 1.000 marks only the two most similar sequences; it does not necessarily mean that these sequences are identical. Lang - Lupinus angustifolius (XP_019412745); Vr - Vigna radiata (XP_014497350); Gm - Glycine max (XP_003530215); Mt - Medicago truncatula (XP_013448893); Ap - Abrus precatorius (XP_027349738); Cc - Cajanus cajan (XP_020206919); Vu - Vigna unguiculata (XP_027927273); Ca - Cicer arietinum (XP_004514393); Tp - Trifolium pratense (PNX74271); Ql - Quercus lobata (XP_030925127); Pa - Prosopis alba (XP_028786489); St - Senna tora (KAF7825648); Qs - Quercus suber (XP_023913890); Tw - Tripterygium wilfordii (KAF5750924); At - Arabidopsis thaliana (OAO90380).

## Slide 22
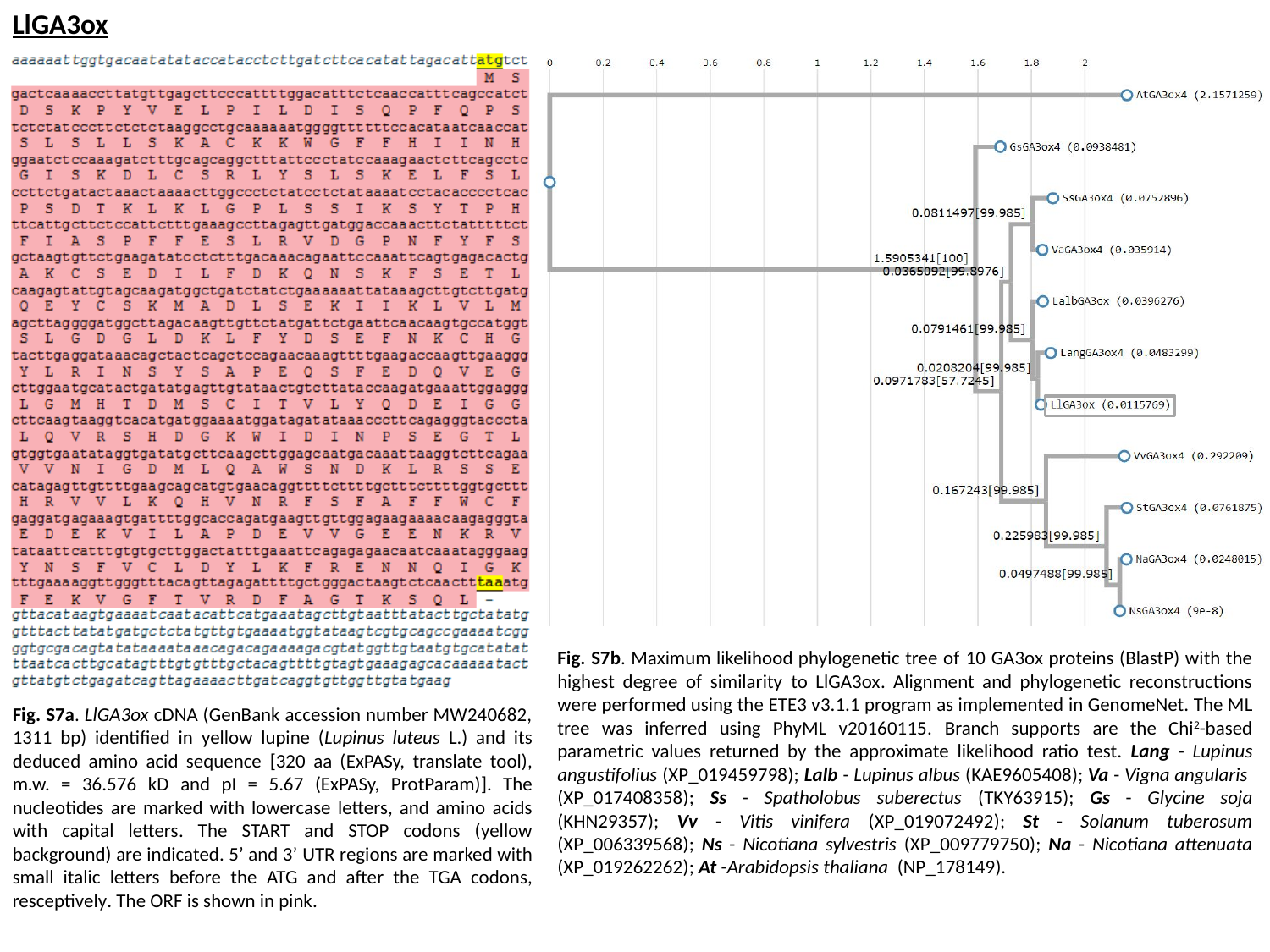

LlGA3ox
Fig. S7b. Maximum likelihood phylogenetic tree of 10 GA3ox proteins (BlastP) with the highest degree of similarity to LlGA3ox. Alignment and phylogenetic reconstructions were performed using the ETE3 v3.1.1 program as implemented in GenomeNet. The ML tree was inferred using PhyML v20160115. Branch supports are the Chi2-based parametric values returned by the approximate likelihood ratio test. Lang - Lupinus angustifolius (XP_019459798); Lalb - Lupinus albus (KAE9605408); Va - Vigna angularis (XP_017408358); Ss - Spatholobus suberectus (TKY63915); Gs - Glycine soja (KHN29357); Vv - Vitis vinifera (XP_019072492); St - Solanum tuberosum (XP_006339568); Ns - Nicotiana sylvestris (XP_009779750); Na - Nicotiana attenuata (XP_019262262); At -Arabidopsis thaliana (NP_178149).
Fig. S7a. LlGA3ox cDNA (GenBank accession number MW240682, 1311 bp) identified in yellow lupine (Lupinus luteus L.) and its deduced amino acid sequence [320 aa (ExPASy, translate tool), m.w. = 36.576 kD and pI = 5.67 (ExPASy, ProtParam)]. The nucleotides are marked with lowercase letters, and amino acids with capital letters. The START and STOP codons (yellow background) are indicated. 5’ and 3’ UTR regions are marked with small italic letters before the ATG and after the TGA codons, resceptively. The ORF is shown in pink.

## Slide 23
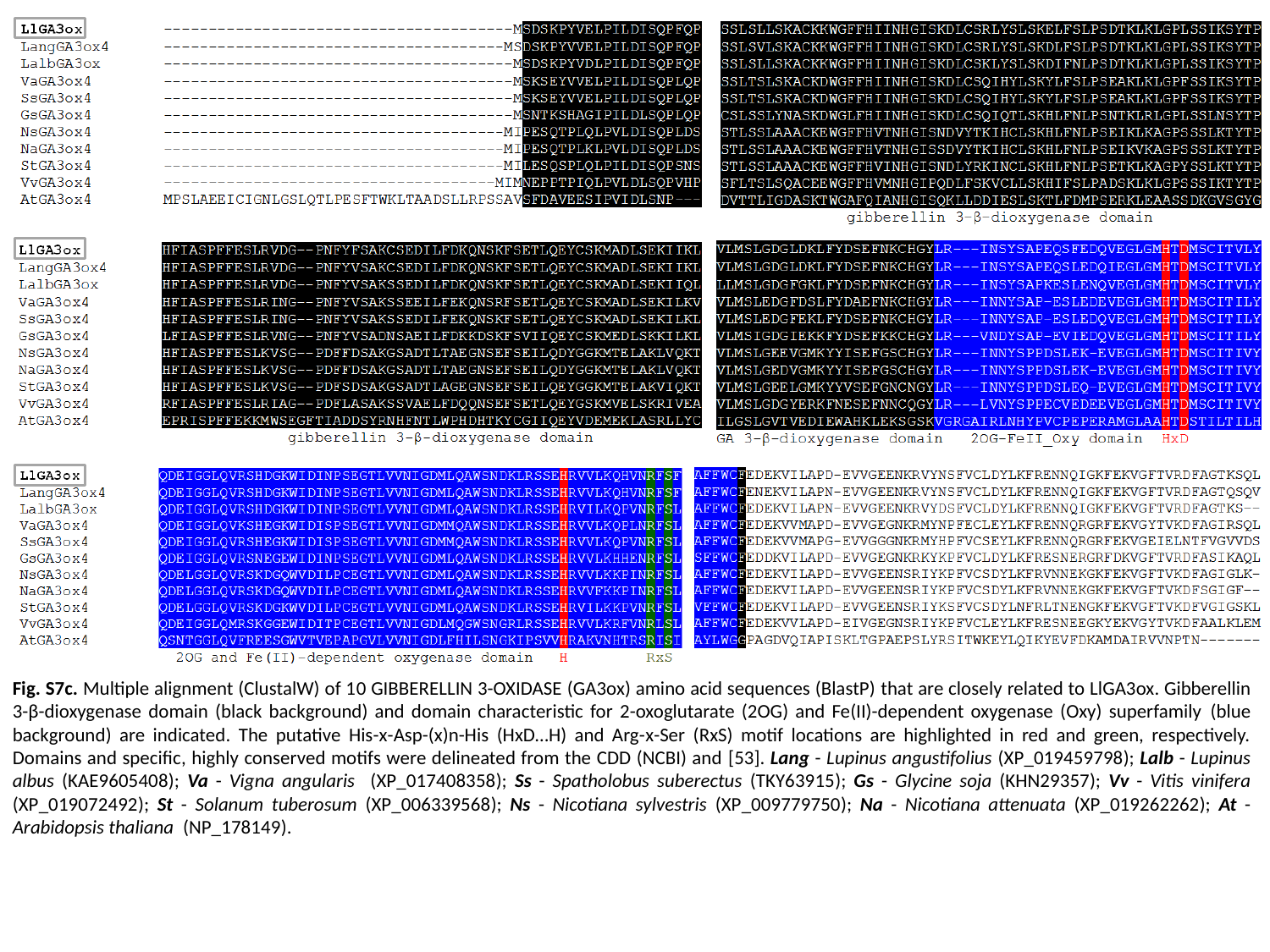

Fig. S7c. Multiple alignment (ClustalW) of 10 GIBBERELLIN 3-OXIDASE (GA3ox) amino acid sequences (BlastP) that are closely related to LlGA3ox. Gibberellin 3-β-dioxygenase domain (black background) and domain characteristic for 2-oxoglutarate (2OG) and Fe(II)-dependent oxygenase (Oxy) superfamily (blue background) are indicated. The putative His-x-Asp-(x)n-His (HxD…H) and Arg-x-Ser (RxS) motif locations are highlighted in red and green, respectively. Domains and specific, highly conserved motifs were delineated from the CDD (NCBI) and [53]. Lang - Lupinus angustifolius (XP_019459798); Lalb - Lupinus albus (KAE9605408); Va - Vigna angularis (XP_017408358); Ss - Spatholobus suberectus (TKY63915); Gs - Glycine soja (KHN29357); Vv - Vitis vinifera (XP_019072492); St - Solanum tuberosum (XP_006339568); Ns - Nicotiana sylvestris (XP_009779750); Na - Nicotiana attenuata (XP_019262262); At -Arabidopsis thaliana (NP_178149).

## Slide 24
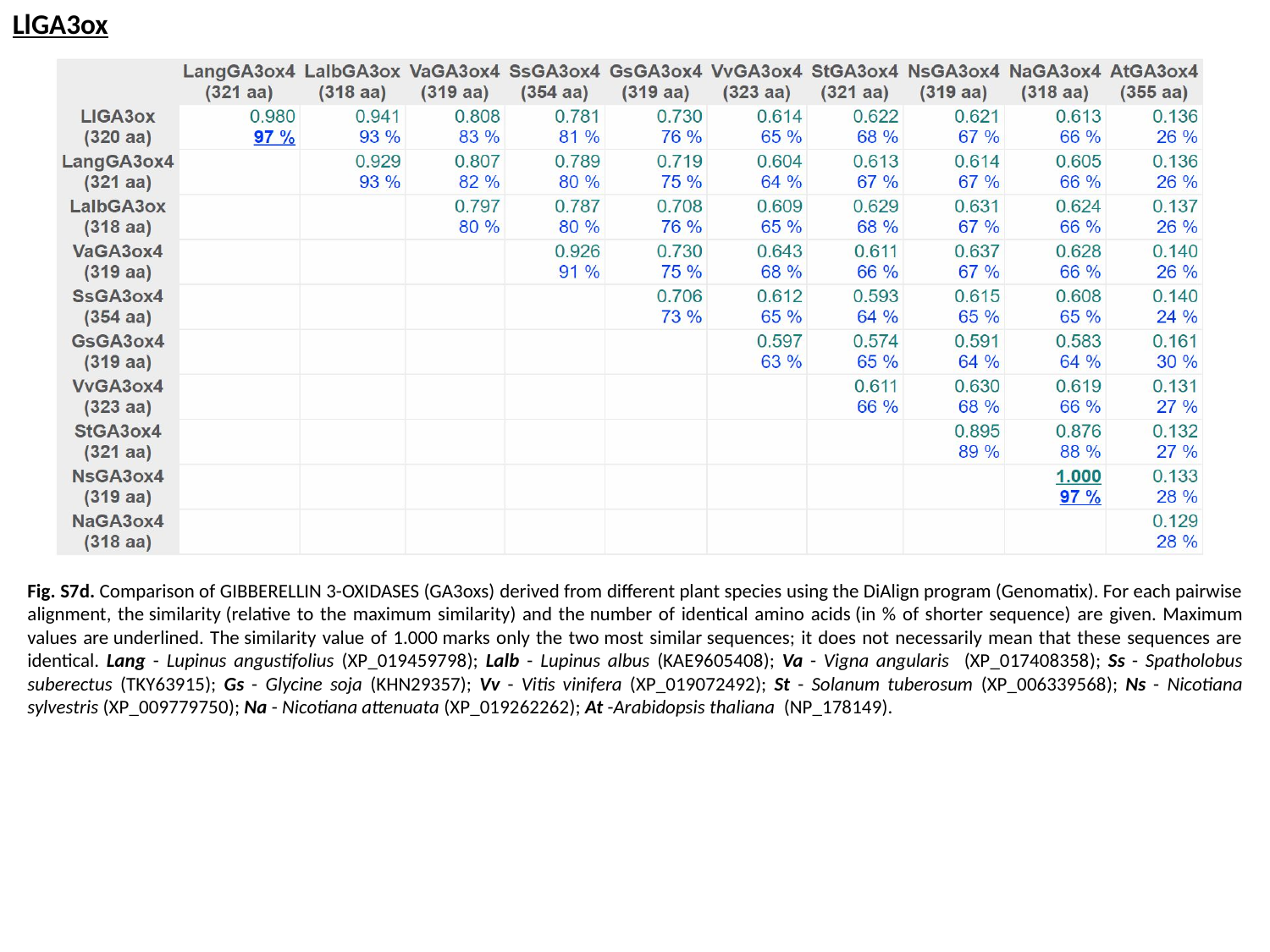

LlGA3ox
Fig. S7d. Comparison of GIBBERELLIN 3-OXIDASES (GA3oxs) derived from different plant species using the DiAlign program (Genomatix). For each pairwise alignment, the similarity (relative to the maximum similarity) and the number of identical amino acids (in % of shorter sequence) are given. Maximum values are underlined. The similarity value of 1.000 marks only the two most similar sequences; it does not necessarily mean that these sequences are identical. Lang - Lupinus angustifolius (XP_019459798); Lalb - Lupinus albus (KAE9605408); Va - Vigna angularis (XP_017408358); Ss - Spatholobus suberectus (TKY63915); Gs - Glycine soja (KHN29357); Vv - Vitis vinifera (XP_019072492); St - Solanum tuberosum (XP_006339568); Ns - Nicotiana sylvestris (XP_009779750); Na - Nicotiana attenuata (XP_019262262); At -Arabidopsis thaliana (NP_178149).

## Slide 25
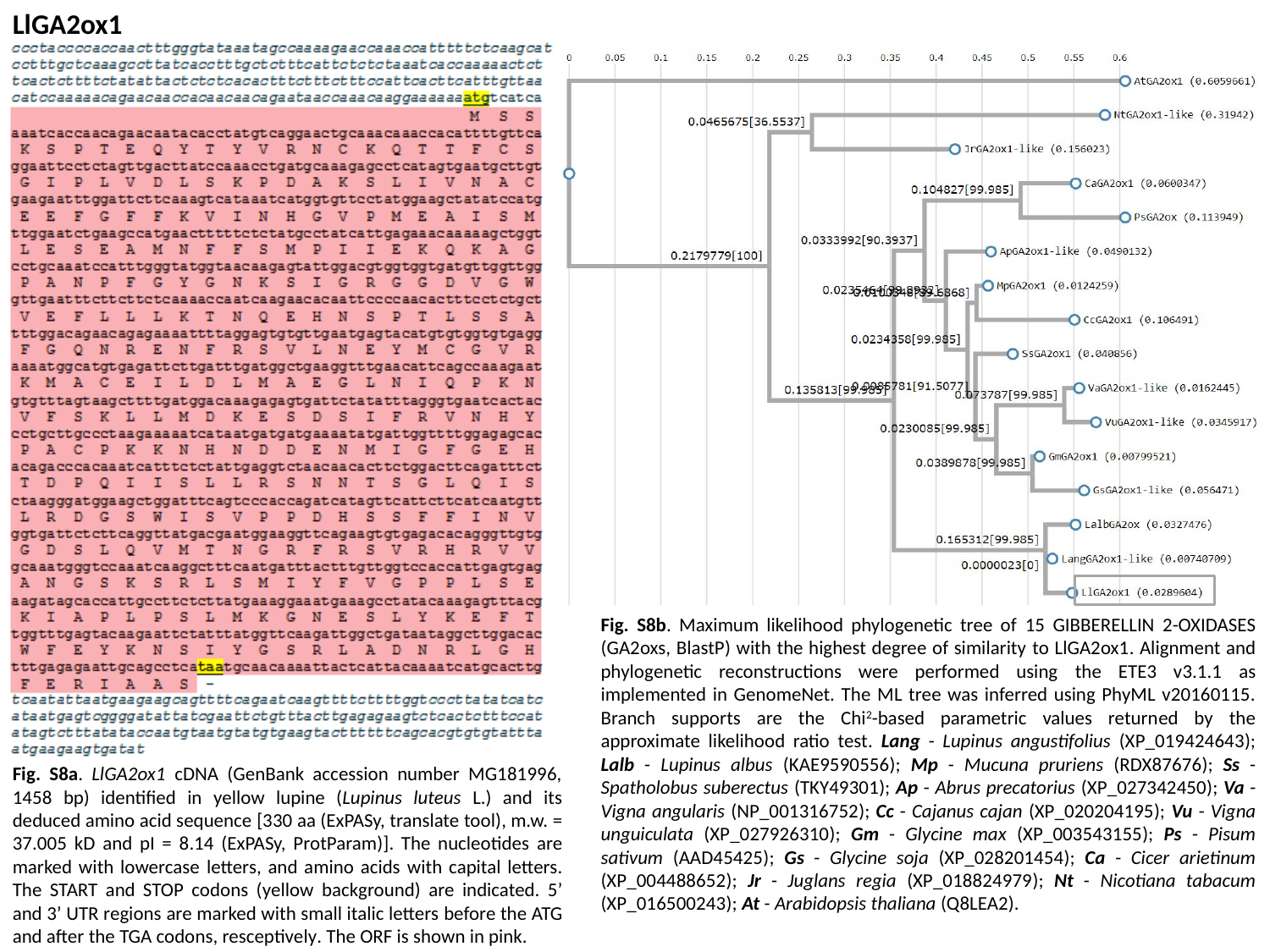

LlGA2ox1
Fig. S8b. Maximum likelihood phylogenetic tree of 15 GIBBERELLIN 2-OXIDASES (GA2oxs, BlastP) with the highest degree of similarity to LlGA2ox1. Alignment and phylogenetic reconstructions were performed using the ETE3 v3.1.1 as implemented in GenomeNet. The ML tree was inferred using PhyML v20160115. Branch supports are the Chi2-based parametric values returned by the approximate likelihood ratio test. Lang - Lupinus angustifolius (XP_019424643); Lalb - Lupinus albus (KAE9590556); Mp - Mucuna pruriens (RDX87676); Ss - Spatholobus suberectus (TKY49301); Ap - Abrus precatorius (XP_027342450); Va - Vigna angularis (NP_001316752); Cc - Cajanus cajan (XP_020204195); Vu - Vigna unguiculata (XP_027926310); Gm - Glycine max (XP_003543155); Ps - Pisum sativum (AAD45425); Gs - Glycine soja (XP_028201454); Ca - Cicer arietinum (XP_004488652); Jr - Juglans regia (XP_018824979); Nt - Nicotiana tabacum (XP_016500243); At - Arabidopsis thaliana (Q8LEA2).
Fig. S8a. LlGA2ox1 cDNA (GenBank accession number MG181996, 1458 bp) identified in yellow lupine (Lupinus luteus L.) and its deduced amino acid sequence [330 aa (ExPASy, translate tool), m.w. = 37.005 kD and pI = 8.14 (ExPASy, ProtParam)]. The nucleotides are marked with lowercase letters, and amino acids with capital letters. The START and STOP codons (yellow background) are indicated. 5’ and 3’ UTR regions are marked with small italic letters before the ATG and after the TGA codons, resceptively. The ORF is shown in pink.

## Slide 26
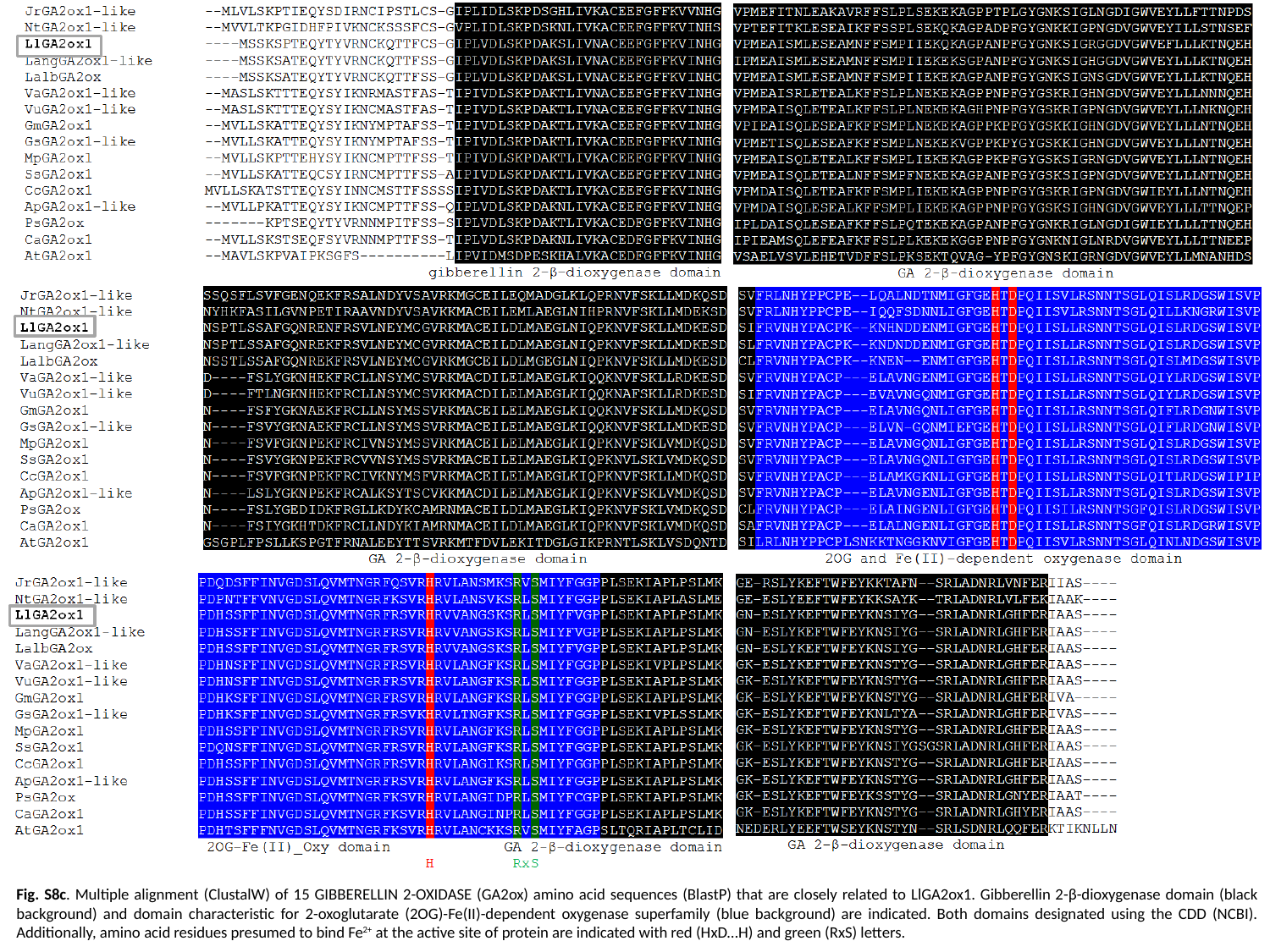

Fig. S8c. Multiple alignment (ClustalW) of 15 GIBBERELLIN 2-OXIDASE (GA2ox) amino acid sequences (BlastP) that are closely related to LlGA2ox1. Gibberellin 2-β-dioxygenase domain (black background) and domain characteristic for 2-oxoglutarate (2OG)-Fe(II)-dependent oxygenase superfamily (blue background) are indicated. Both domains designated using the CDD (NCBI). Additionally, amino acid residues presumed to bind Fe2+ at the active site of protein are indicated with red (HxD…H) and green (RxS) letters.

## Slide 27
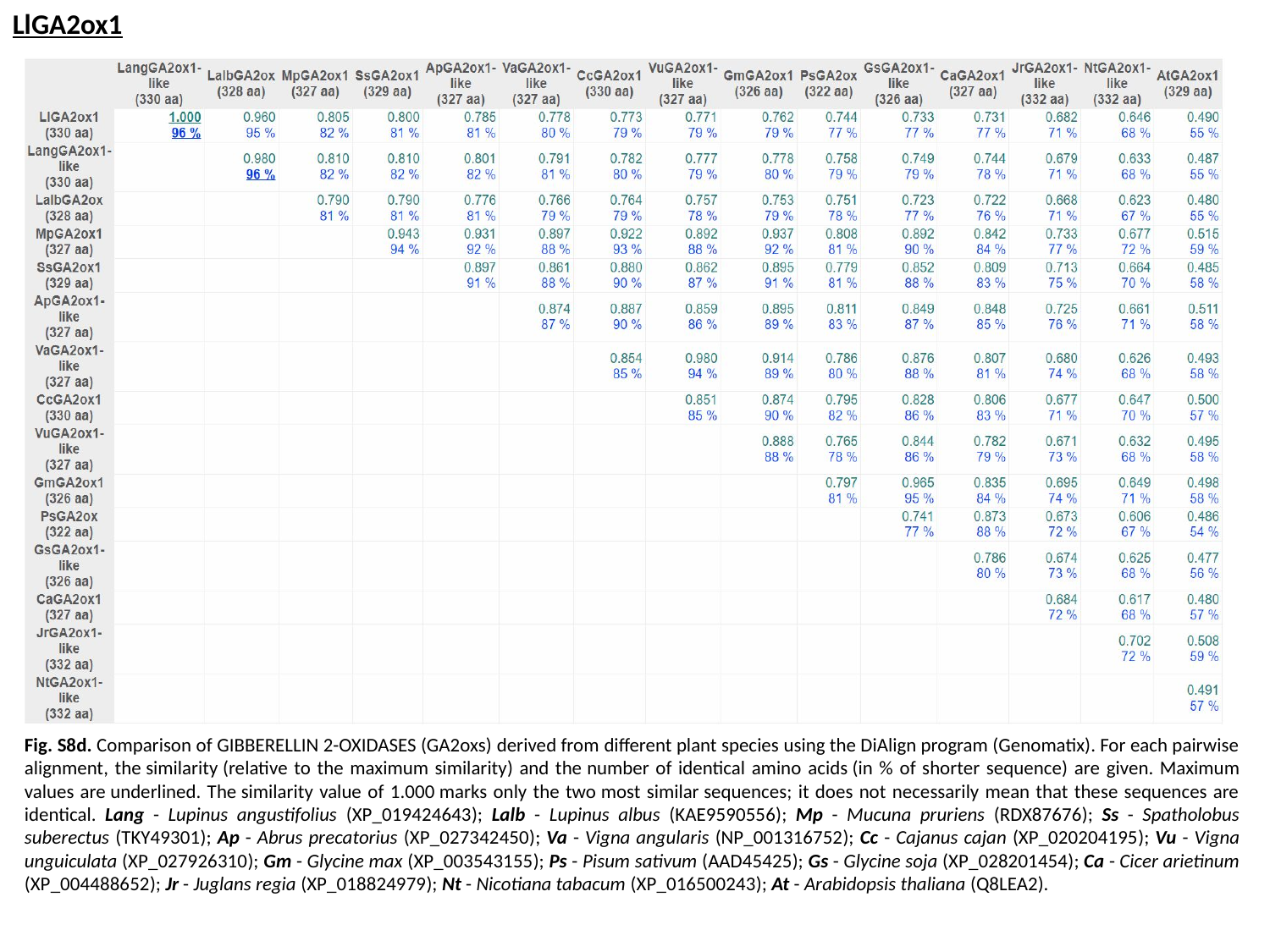

LlGA2ox1
Fig. S8d. Comparison of GIBBERELLIN 2-OXIDASES (GA2oxs) derived from different plant species using the DiAlign program (Genomatix). For each pairwise alignment, the similarity (relative to the maximum similarity) and the number of identical amino acids (in % of shorter sequence) are given. Maximum values are underlined. The similarity value of 1.000 marks only the two most similar sequences; it does not necessarily mean that these sequences are identical. Lang - Lupinus angustifolius (XP_019424643); Lalb - Lupinus albus (KAE9590556); Mp - Mucuna pruriens (RDX87676); Ss - Spatholobus suberectus (TKY49301); Ap - Abrus precatorius (XP_027342450); Va - Vigna angularis (NP_001316752); Cc - Cajanus cajan (XP_020204195); Vu - Vigna unguiculata (XP_027926310); Gm - Glycine max (XP_003543155); Ps - Pisum sativum (AAD45425); Gs - Glycine soja (XP_028201454); Ca - Cicer arietinum (XP_004488652); Jr - Juglans regia (XP_018824979); Nt - Nicotiana tabacum (XP_016500243); At - Arabidopsis thaliana (Q8LEA2).

## Slide 28
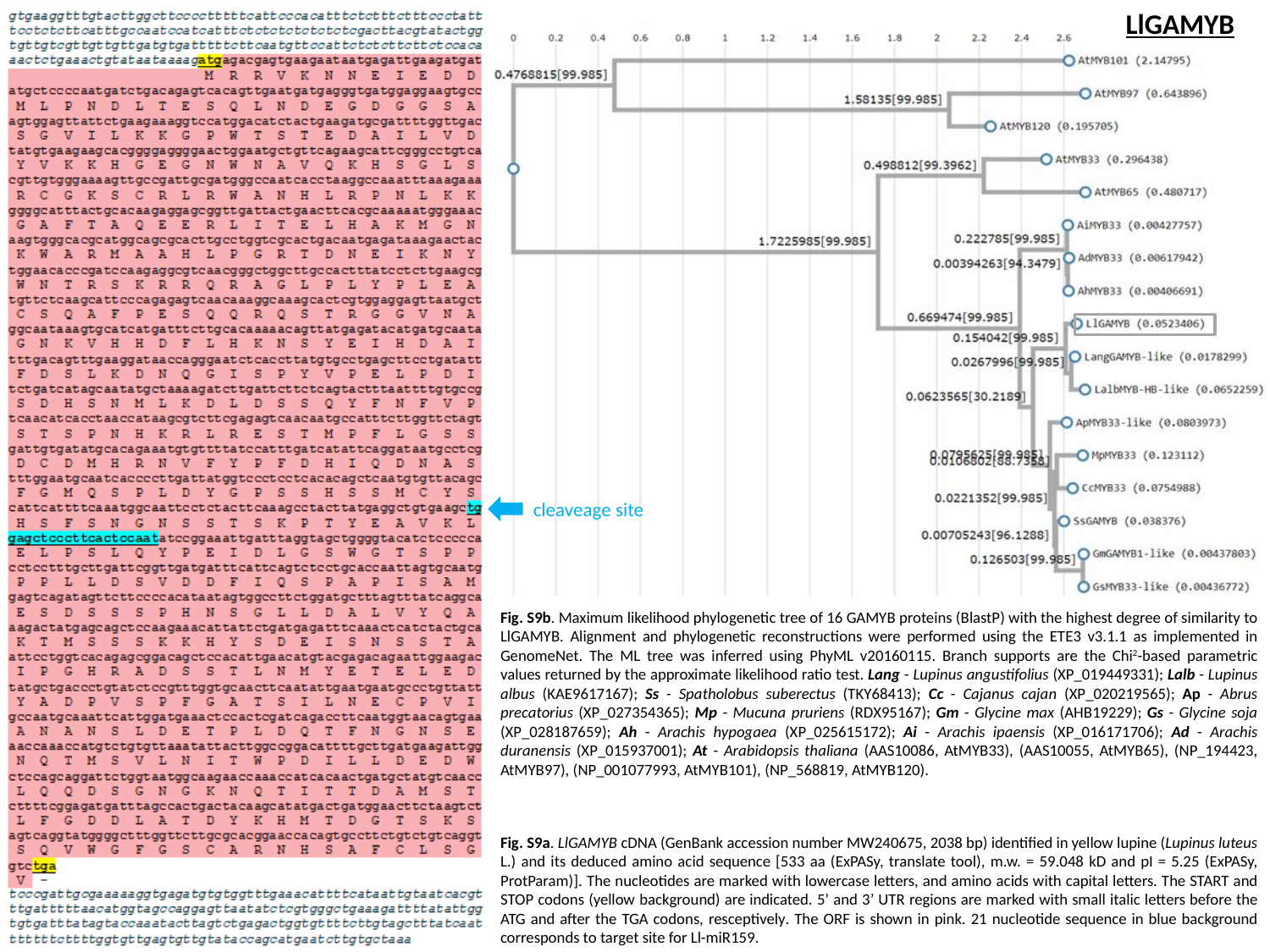

LlGAMYB
cleaveage site
Fig. S9b. Maximum likelihood phylogenetic tree of 16 GAMYB proteins (BlastP) with the highest degree of similarity to LlGAMYB. Alignment and phylogenetic reconstructions were performed using the ETE3 v3.1.1 as implemented in GenomeNet. The ML tree was inferred using PhyML v20160115. Branch supports are the Chi2-based parametric values returned by the approximate likelihood ratio test. Lang - Lupinus angustifolius (XP_019449331); Lalb - Lupinus albus (KAE9617167); Ss - Spatholobus suberectus (TKY68413); Cc - Cajanus cajan (XP_020219565); Ap - Abrus precatorius (XP_027354365); Mp - Mucuna pruriens (RDX95167); Gm - Glycine max (AHB19229); Gs - Glycine soja (XP_028187659); Ah - Arachis hypogaea (XP_025615172); Ai - Arachis ipaensis (XP_016171706); Ad - Arachis duranensis (XP_015937001); At - Arabidopsis thaliana (AAS10086, AtMYB33), (AAS10055, AtMYB65), (NP_194423, AtMYB97), (NP_001077993, AtMYB101), (NP_568819, AtMYB120).
Fig. S9a. LlGAMYB cDNA (GenBank accession number MW240675, 2038 bp) identified in yellow lupine (Lupinus luteus L.) and its deduced amino acid sequence [533 aa (ExPASy, translate tool), m.w. = 59.048 kD and pI = 5.25 (ExPASy, ProtParam)]. The nucleotides are marked with lowercase letters, and amino acids with capital letters. The START and STOP codons (yellow background) are indicated. 5’ and 3’ UTR regions are marked with small italic letters before the ATG and after the TGA codons, resceptively. The ORF is shown in pink. 21 nucleotide sequence in blue background corresponds to target site for Ll-miR159.

## Slide 29
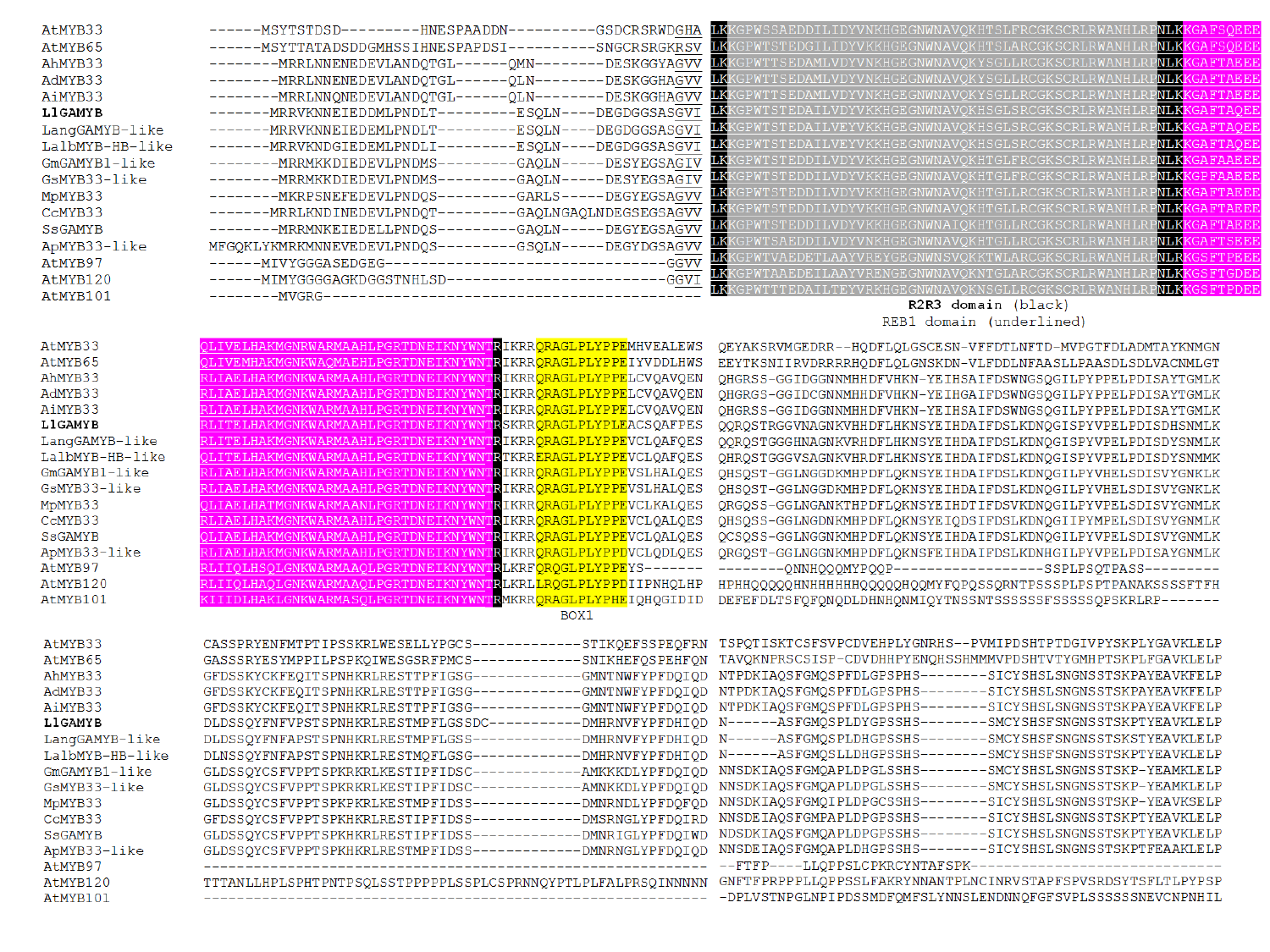

## Slide 30
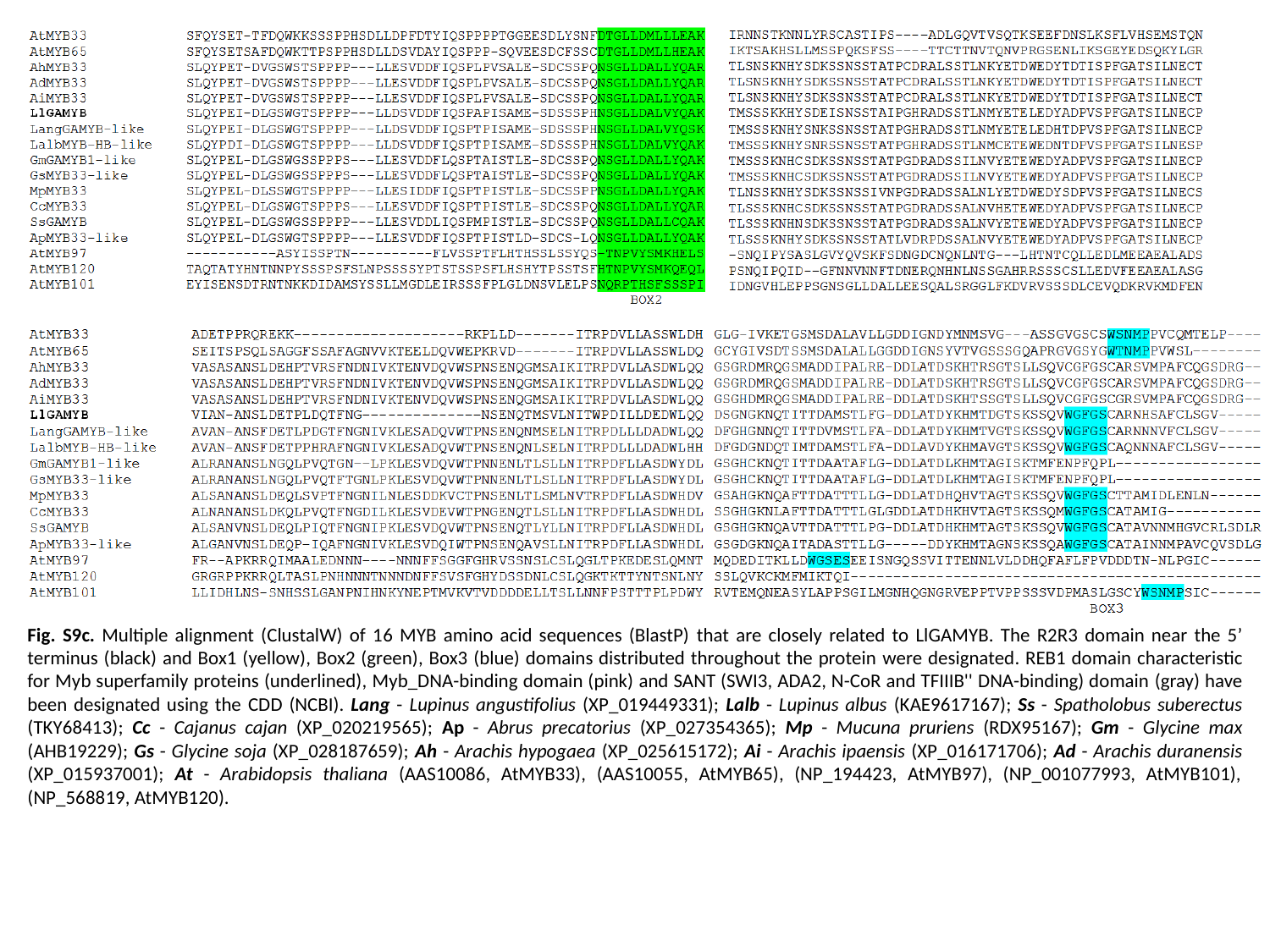

Fig. S9c. Multiple alignment (ClustalW) of 16 MYB amino acid sequences (BlastP) that are closely related to LlGAMYB. The R2R3 domain near the 5’ terminus (black) and Box1 (yellow), Box2 (green), Box3 (blue) domains distributed throughout the protein were designated. REB1 domain characteristic for Myb superfamily proteins (underlined), Myb_DNA-binding domain (pink) and SANT (SWI3, ADA2, N-CoR and TFIIIB'' DNA-binding) domain (gray) have been designated using the CDD (NCBI). Lang - Lupinus angustifolius (XP_019449331); Lalb - Lupinus albus (KAE9617167); Ss - Spatholobus suberectus (TKY68413); Cc - Cajanus cajan (XP_020219565); Ap - Abrus precatorius (XP_027354365); Mp - Mucuna pruriens (RDX95167); Gm - Glycine max (AHB19229); Gs - Glycine soja (XP_028187659); Ah - Arachis hypogaea (XP_025615172); Ai - Arachis ipaensis (XP_016171706); Ad - Arachis duranensis (XP_015937001); At - Arabidopsis thaliana (AAS10086, AtMYB33), (AAS10055, AtMYB65), (NP_194423, AtMYB97), (NP_001077993, AtMYB101), (NP_568819, AtMYB120).

## Slide 31
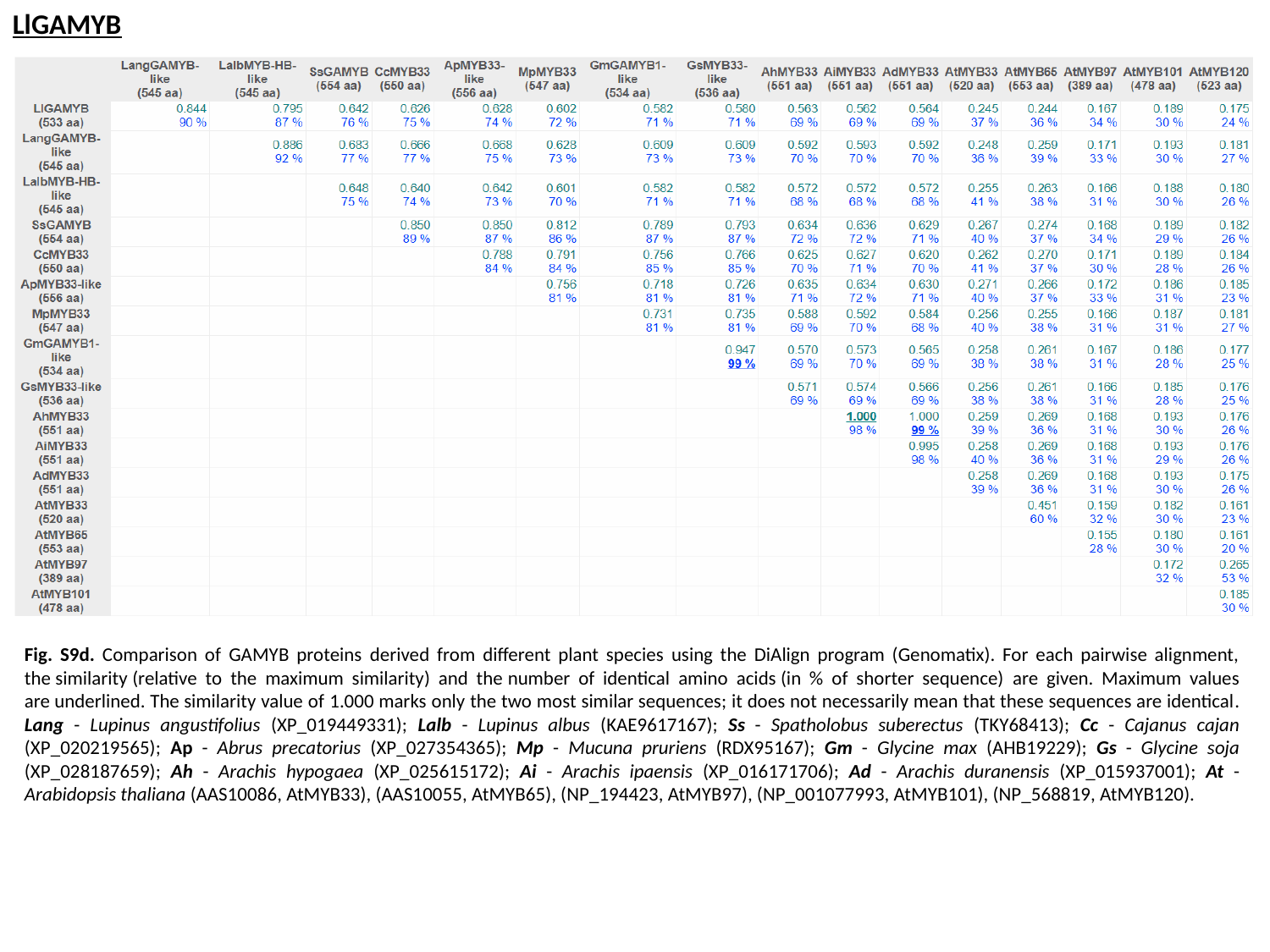

LlGAMYB
Fig. S9d. Comparison of GAMYB proteins derived from different plant species using the DiAlign program (Genomatix). For each pairwise alignment, the similarity (relative to the maximum similarity) and the number of identical amino acids (in % of shorter sequence) are given. Maximum values are underlined. The similarity value of 1.000 marks only the two most similar sequences; it does not necessarily mean that these sequences are identical. Lang - Lupinus angustifolius (XP_019449331); Lalb - Lupinus albus (KAE9617167); Ss - Spatholobus suberectus (TKY68413); Cc - Cajanus cajan (XP_020219565); Ap - Abrus precatorius (XP_027354365); Mp - Mucuna pruriens (RDX95167); Gm - Glycine max (AHB19229); Gs - Glycine soja (XP_028187659); Ah - Arachis hypogaea (XP_025615172); Ai - Arachis ipaensis (XP_016171706); Ad - Arachis duranensis (XP_015937001); At - Arabidopsis thaliana (AAS10086, AtMYB33), (AAS10055, AtMYB65), (NP_194423, AtMYB97), (NP_001077993, AtMYB101), (NP_568819, AtMYB120).

## Slide 32
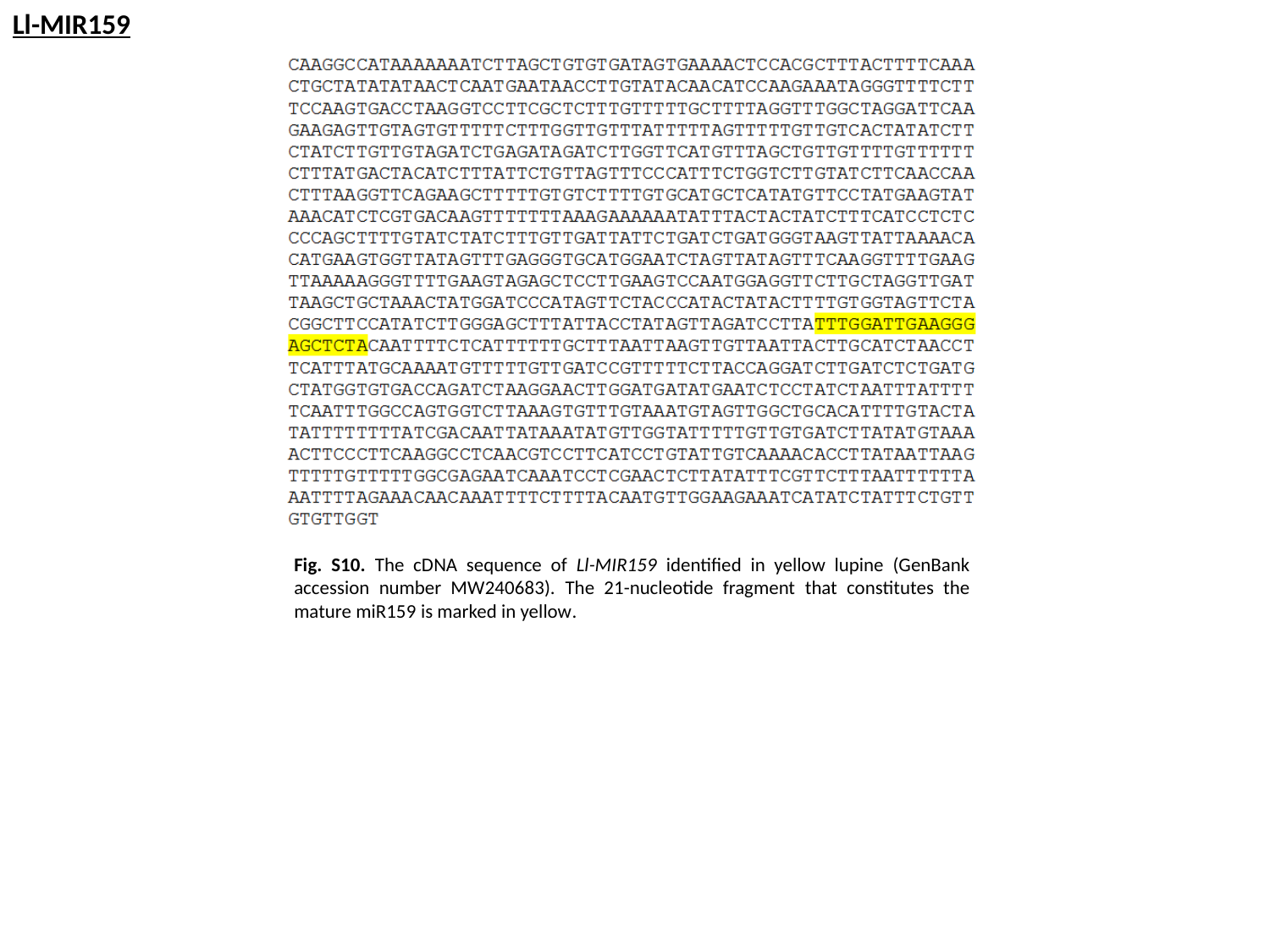

Ll-MIR159
Fig. S10. The cDNA sequence of Ll-MIR159 identified in yellow lupine (GenBank accession number MW240683). The 21-nucleotide fragment that constitutes the mature miR159 is marked in yellow.

## Slide 33
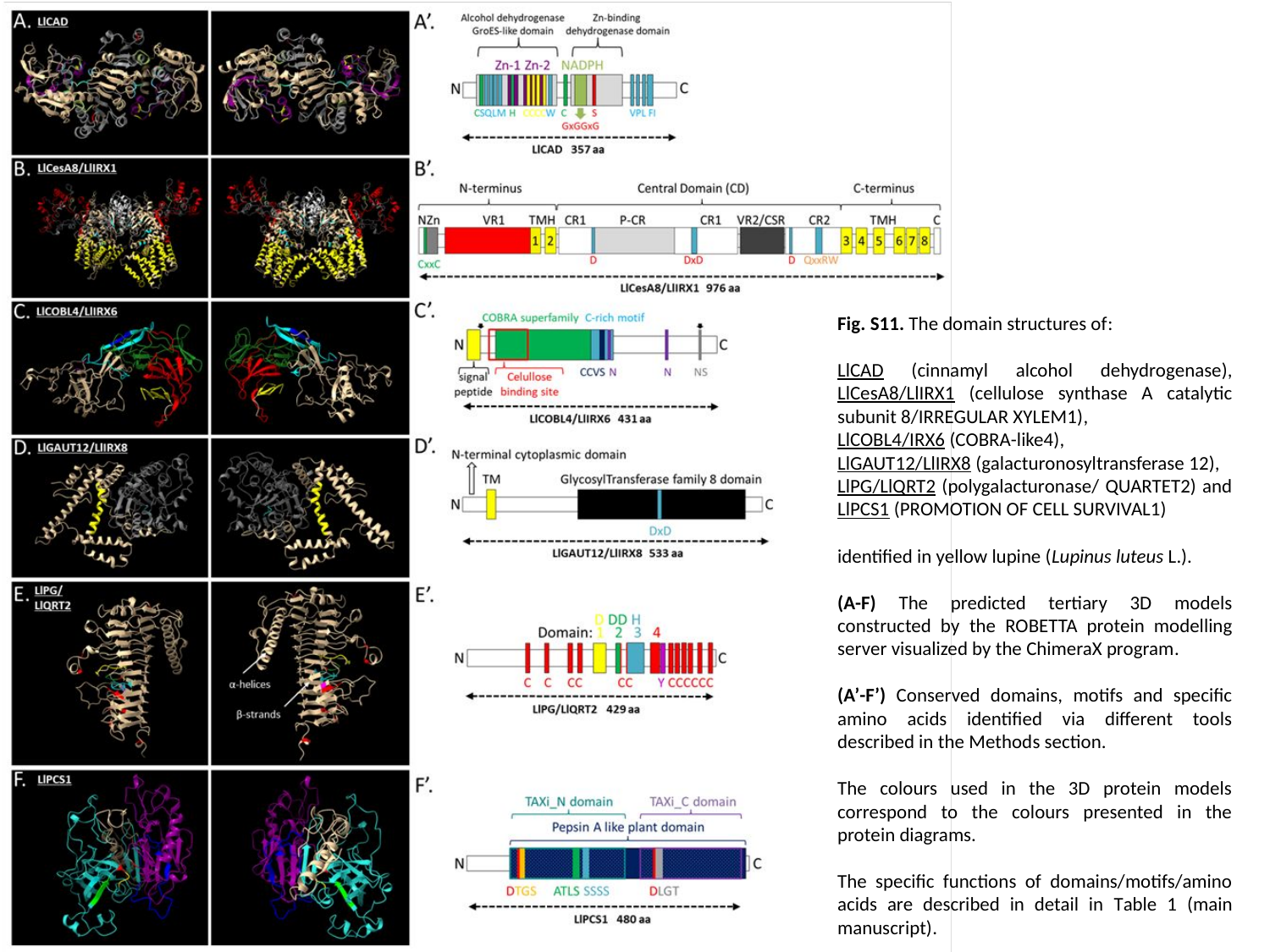

Fig. S11. The domain structures of:
LlCAD (cinnamyl alcohol dehydrogenase), LlCesA8/LlIRX1 (cellulose synthase A catalytic subunit 8/IRREGULAR XYLEM1),
LlCOBL4/IRX6 (COBRA-like4),
LlGAUT12/LlIRX8 (galacturonosyltransferase 12),
LlPG/LlQRT2 (polygalacturonase/ QUARTET2) and LlPCS1 (PROMOTION OF CELL SURVIVAL1)
identified in yellow lupine (Lupinus luteus L.).
(A-F) The predicted tertiary 3D models constructed by the ROBETTA protein modelling server visualized by the ChimeraX program.
(A’-F’) Conserved domains, motifs and specific amino acids identified via different tools described in the Methods section.
The colours used in the 3D protein models correspond to the colours presented in the protein diagrams.
The specific functions of domains/motifs/amino acids are described in detail in Table 1 (main manuscript).

## Slide 34
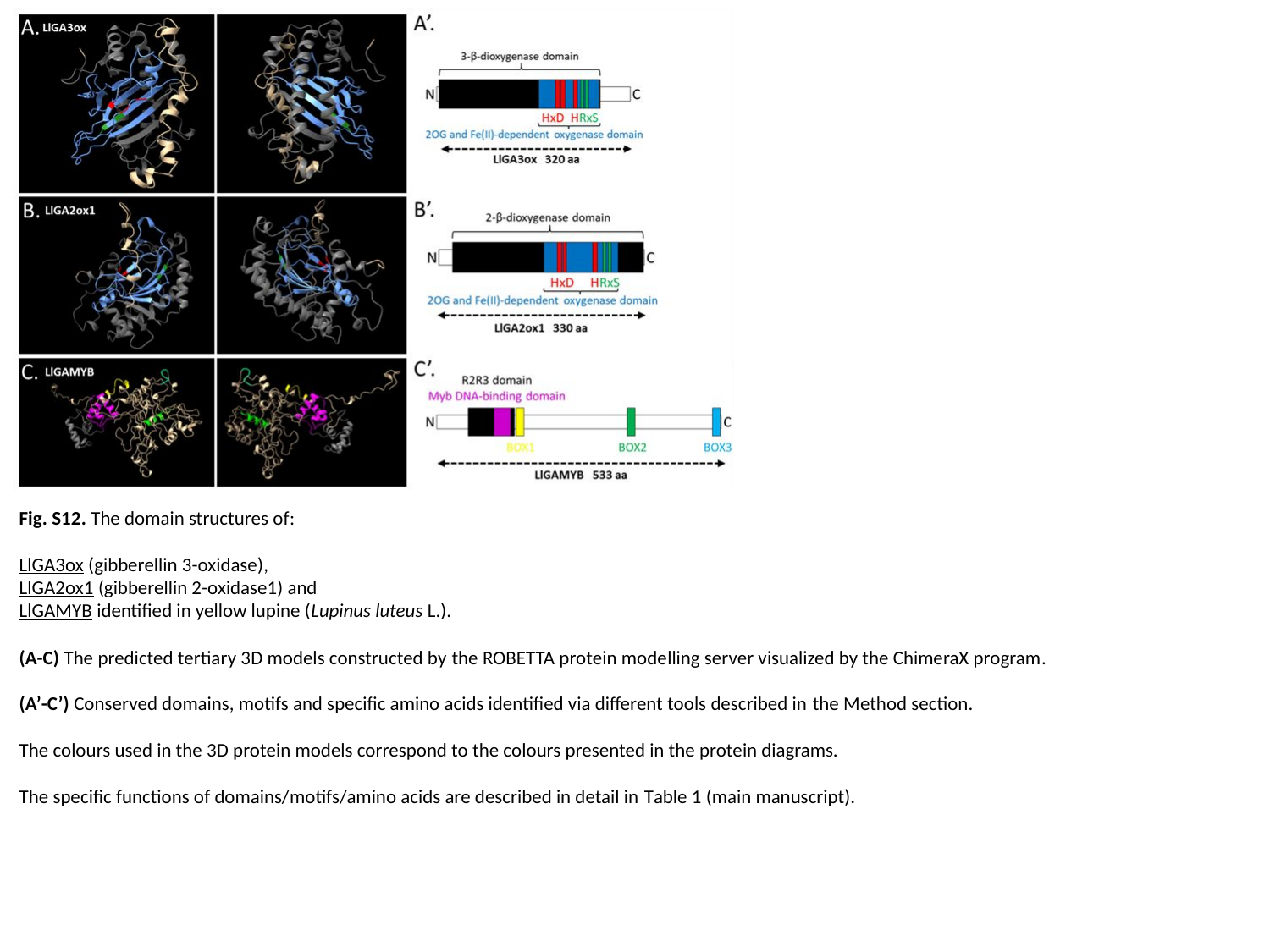

Fig. S12. The domain structures of:
LlGA3ox (gibberellin 3-oxidase),
LlGA2ox1 (gibberellin 2-oxidase1) and
LlGAMYB identified in yellow lupine (Lupinus luteus L.).
(A-C) The predicted tertiary 3D models constructed by the ROBETTA protein modelling server visualized by the ChimeraX program.
(A’-C’) Conserved domains, motifs and specific amino acids identified via different tools described in the Method section.
The colours used in the 3D protein models correspond to the colours presented in the protein diagrams.
The specific functions of domains/motifs/amino acids are described in detail in Table 1 (main manuscript).

## Slide 35
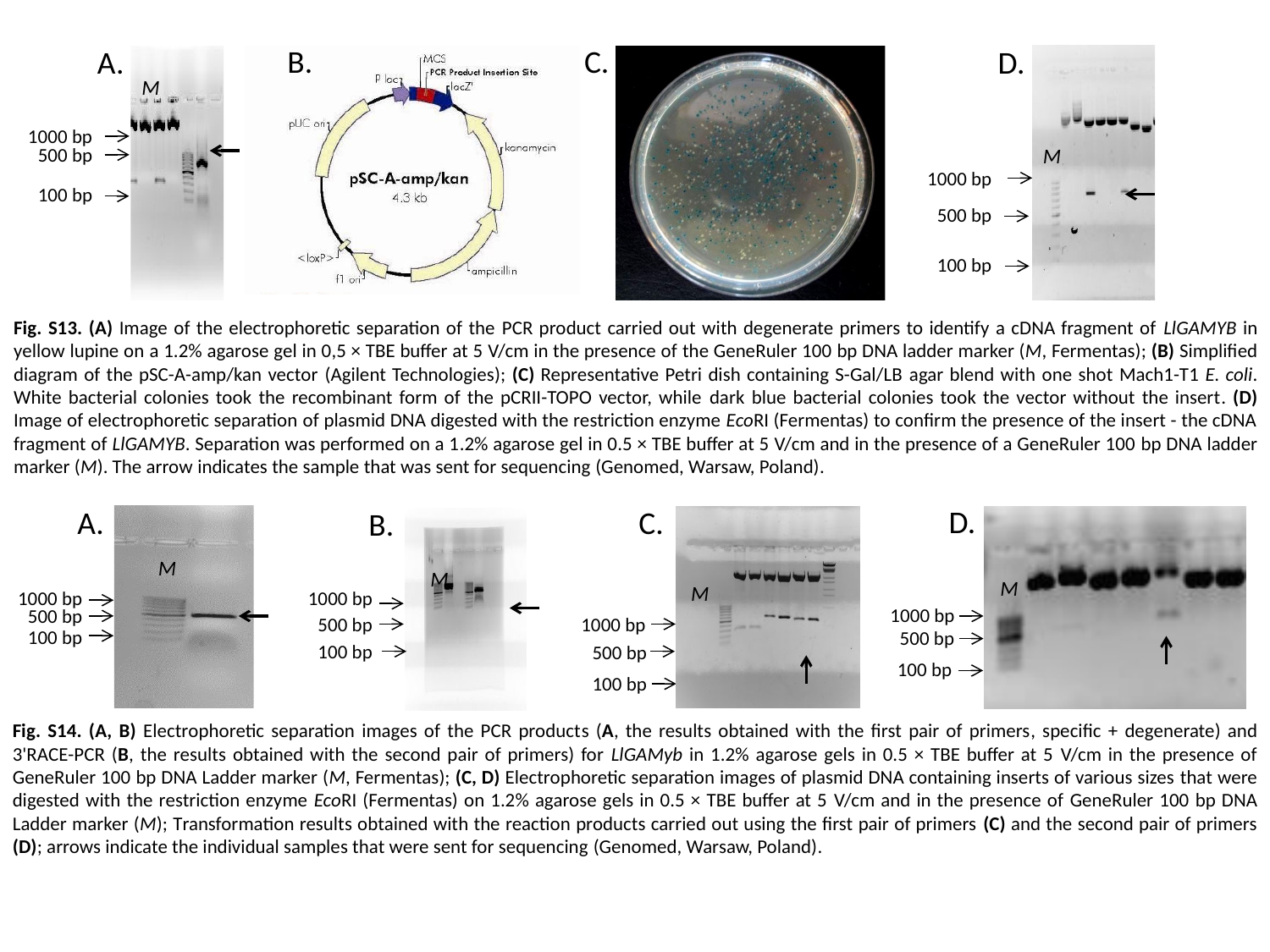

B.
C.
A.
D.
M
1000 bp
500 bp
M
1000 bp
100 bp
500 bp
100 bp
Fig. S13. (A) Image of the electrophoretic separation of the PCR product carried out with degenerate primers to identify a cDNA fragment of LlGAMYB in yellow lupine on a 1.2% agarose gel in 0,5 × TBE buffer at 5 V/cm in the presence of the GeneRuler 100 bp DNA ladder marker (M, Fermentas); (B) Simplified diagram of the pSC-A-amp/kan vector (Agilent Technologies); (C) Representative Petri dish containing S-Gal/LB agar blend with one shot Mach1-T1 E. coli. White bacterial colonies took the recombinant form of the pCRII-TOPO vector, while dark blue bacterial colonies took the vector without the insert. (D) Image of electrophoretic separation of plasmid DNA digested with the restriction enzyme EcoRI (Fermentas) to confirm the presence of the insert - the cDNA fragment of LlGAMYB. Separation was performed on a 1.2% agarose gel in 0.5 × TBE buffer at 5 V/cm and in the presence of a GeneRuler 100 bp DNA ladder marker (M). The arrow indicates the sample that was sent for sequencing (Genomed, Warsaw, Poland).
D.
A.
C.
B.
M
M
M
M
1000 bp
1000 bp
1000 bp
500 bp
1000 bp
500 bp
100 bp
500 bp
100 bp
500 bp
100 bp
100 bp
Fig. S14. (A, B) Electrophoretic separation images of the PCR products (A, the results obtained with the first pair of primers, specific + degenerate) and 3'RACE-PCR (B, the results obtained with the second pair of primers) for LlGAMyb in 1.2% agarose gels in 0.5 × TBE buffer at 5 V/cm in the presence of GeneRuler 100 bp DNA Ladder marker (M, Fermentas); (C, D) Electrophoretic separation images of plasmid DNA containing inserts of various sizes that were digested with the restriction enzyme EcoRI (Fermentas) on 1.2% agarose gels in 0.5 × TBE buffer at 5 V/cm and in the presence of GeneRuler 100 bp DNA Ladder marker (M); Transformation results obtained with the reaction products carried out using the first pair of primers (C) and the second pair of primers (D); arrows indicate the individual samples that were sent for sequencing (Genomed, Warsaw, Poland).

## Slide 36
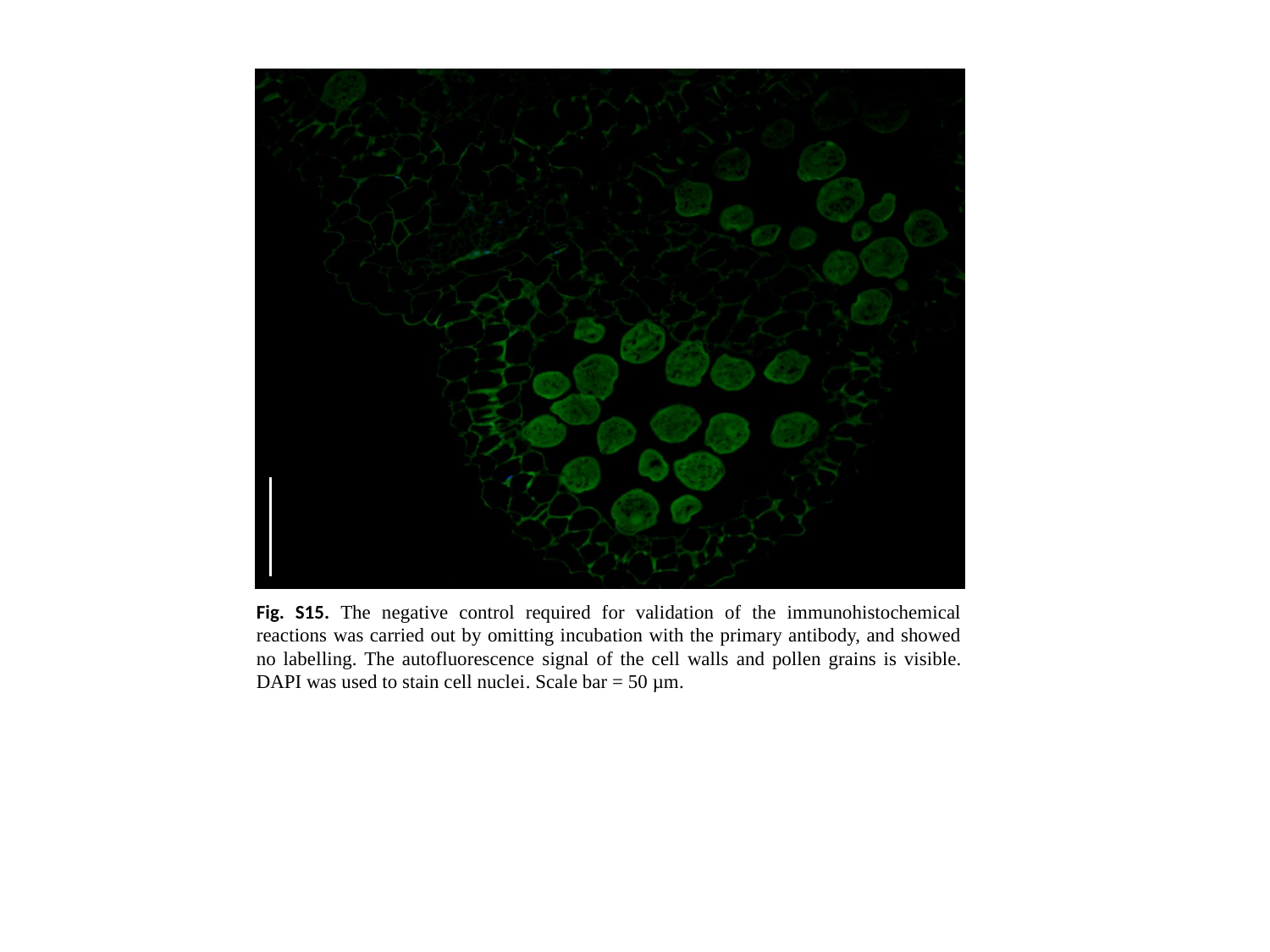

Fig. S15. The negative control required for validation of the immunohistochemical reactions was carried out by omitting incubation with the primary antibody, and showed no labelling. The autofluorescence signal of the cell walls and pollen grains is visible. DAPI was used to stain cell nuclei. Scale bar = 50 µm.

## Slide 37
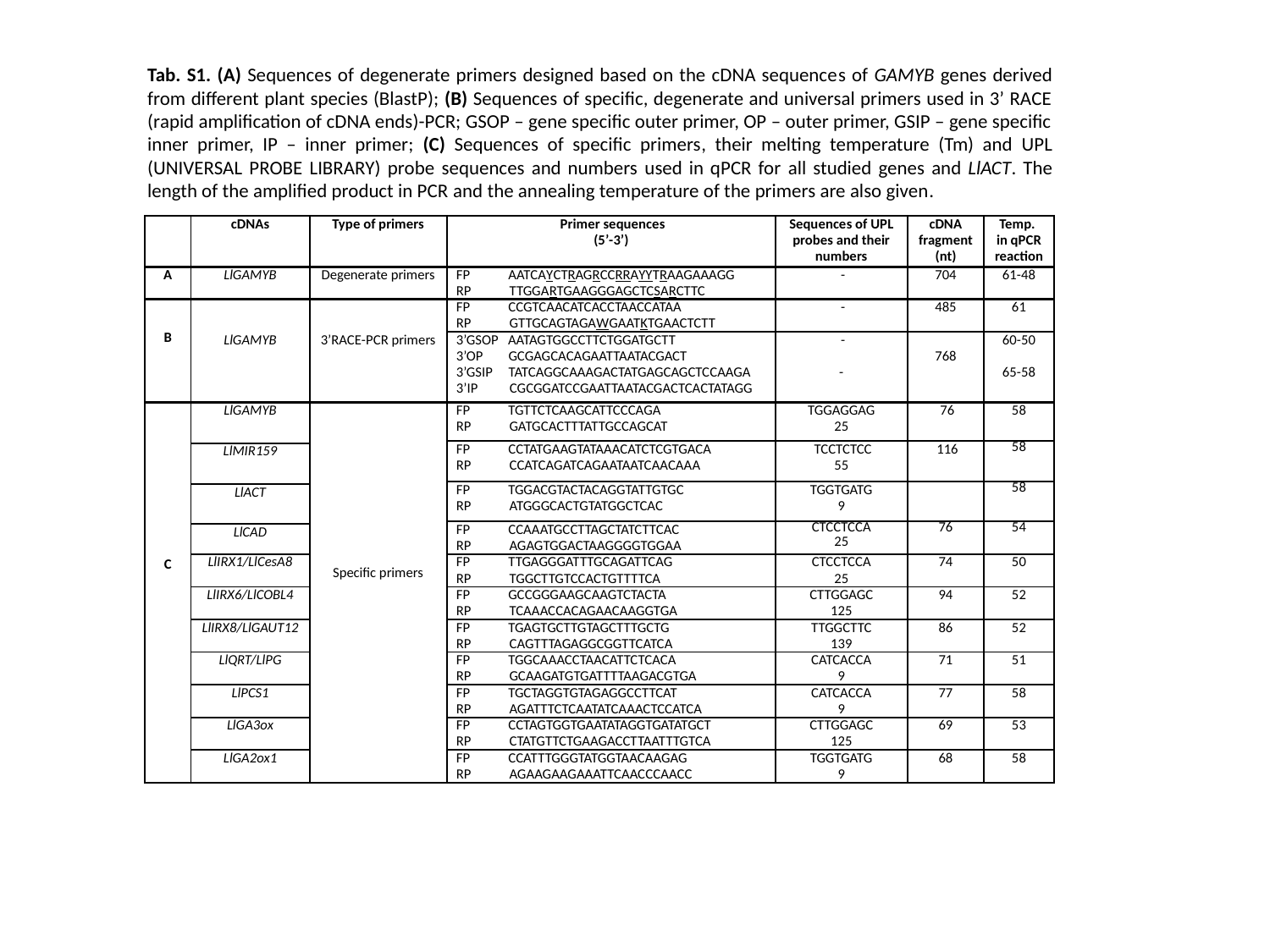

Tab. S1. (A) Sequences of degenerate primers designed based on the cDNA sequences of GAMYB genes derived from different plant species (BlastP); (B) Sequences of specific, degenerate and universal primers used in 3’ RACE (rapid amplification of cDNA ends)-PCR; GSOP – gene specific outer primer, OP – outer primer, GSIP – gene specific inner primer, IP – inner primer; (C) Sequences of specific primers, their melting temperature (Tm) and UPL (UNIVERSAL PROBE LIBRARY) probe sequences and numbers used in qPCR for all studied genes and LlACT. The length of the amplified product in PCR and the annealing temperature of the primers are also given.
| | cDNAs | Type of primers | Primer sequences (5’-3’) | Sequences of UPL probes and their numbers | cDNA fragment (nt) | Temp. in qPCR reaction |
| --- | --- | --- | --- | --- | --- | --- |
| A | LlGAMYB | Degenerate primers | FP AATCAYCTRAGRCCRRAYYTRAAGAAAGG RP TTGGARTGAAGGGAGCTCSARCTTC | - | 704 | 61-48 |
| B | LlGAMYB | 3’RACE-PCR primers | FP CCGTCAACATCACCTAACCATAA RP GTTGCAGTAGAWGAATKTGAACTCTT | - | 485 | 61 |
| | | | 3’GSOP AATAGTGGCCTTCTGGATGCTT 3’OP GCGAGCACAGAATTAATACGACT 3’GSIP TATCAGGCAAAGACTATGAGCAGCTCCAAGA 3’IP CGCGGATCCGAATTAATACGACTCACTATAGG | - - | 768 | 60-50 65-58 |
| C | LlGAMYB | Specific primers | FP TGTTCTCAAGCATTCCCAGA RP GATGCACTTTATTGCCAGCAT | TGGAGGAG 25 | 76 | 58 |
| | | | FP CCTATGAAGTATAAACATCTCGTGACA RP CCATCAGATCAGAATAATCAACAAA | TCCTCTCC 55 | 116 | 58 |
| | LlMIR159 | | | | | |
| | | | FP TGGACGTACTACAGGTATTGTGC RP ATGGGCACTGTATGGCTCAC | TGGTGATG 9 | | 58 |
| | LlACT | | | | | |
| | | | FP CCAAATGCCTTAGCTATCTTCAC RP AGAGTGGACTAAGGGGTGGAA | CTCCTCCA 25 | 76 | 54 |
| | LlCAD | | | | | |
| | LlIRX1/LlCesA8 | | FP TTGAGGGATTTGCAGATTCAG RP TGGCTTGTCCACTGTTTTCA | CTCCTCCA 25 | 74 | 50 |
| | LlIRX6/LlCOBL4 | | FP GCCGGGAAGCAAGTCTACTA RP TCAAACCACAGAACAAGGTGA | CTTGGAGC 125 | 94 | 52 |
| | LlIRX8/LlGAUT12 | | FP TGAGTGCTTGTAGCTTTGCTG RP CAGTTTAGAGGCGGTTCATCA | TTGGCTTC 139 | 86 | 52 |
| | LlQRT/LlPG | | FP TGGCAAACCTAACATTCTCACA RP GCAAGATGTGATTTTAAGACGTGA | CATCACCA 9 | 71 | 51 |
| | LlPCS1 | | FP TGCTAGGTGTAGAGGCCTTCAT RP AGATTTCTCAATATCAAACTCCATCA | CATCACCA 9 | 77 | 58 |
| | LlGA3ox | | FP CCTAGTGGTGAATATAGGTGATATGCT RP CTATGTTCTGAAGACCTTAATTTGTCA | CTTGGAGC 125 | 69 | 53 |
| | LlGA2ox1 | | FP CCATTTGGGTATGGTAACAAGAG RP AGAAGAAGAAATTCAACCCAACC | TGGTGATG 9 | 68 | 58 |
